# Supplementary material for: Quantification of water requirement of some major crops under semi-arid climate in Turkey
Source: PeerJ. 2022 Jul 7;10:e13696. doi: 10.7717/peerj.13696 (PMC9271273; doi:10.7717/peerj.13696)
Supplement: Supplemental Information 5 — The crop coefficients and yield response factor and some other parameters related with pistachio were taken local studies and also from this PhD thesis. [file peerj-10-13696-s005.pdf]

151770

ÇUKUROVA ÜNİVERSİTESİ
FEN BİLİMLERİ ENSTİTÜSÜ

DOKTORA TEZİ

YUSUF AYDIN

ANTEPFISTIĞINDA FARKLI SU VE AZOT DÜZEYLERİNİN VERİM VE PERİYODİSİTE ÜZERİNE ETKİLERİ

TARIMSAL YAPILAR VE SULAMA ANABİLİM DALI

ADANA, 2004

151770

ÇUKUROVA ÜNİVERSİTESİ
FEN BİLİMLERİ ENSTİTÜSÜ

ANTEPFISTIĞINDA FARKLI SU VE AZOT DÜZEYLERİNİN VERİM VE PERİYODİSİTE ÜZERİNE ETKİLERİ

YUSUF AYDIN

DOKTORA TEZİ

TARIMSAL YAPILAR VE SULAMA ANABİLİM DALI

Bu Tez 23.06.2004 Tarihinde Aşağıdaki Jüri Üyeleri Tarafından Oybirliği İle Kabul Edilmiştir.

Prof. Dr. Rıza KANBER	Prof. Dr. Cevat KIRDA	Prof. Dr. Ayzin KÜDEN
DANIŞMAN	ÜYE	ÜYE

Doç. Dr. Sermet ÖNDER	Yrd. Doç. Dr. Mustafa ÜNLÜ
ÜYE	ÜYE

Bu Tez Enstitümüz Tarımsal Yapılar ve Sulama Anabilim Dalında Hazırlanmıştır.
Kod No: 831

T.C. ÇUKUROVA ÜNİVERSİTESİ FEN BİLİMLERİ ENSTİTÜSÜ MÜDÜRLÜĞÜ 1973

Prof. Dr. Fikri AKDENİZ
Enstitü Müdürü

Bu çalışma TÜBİTAK tarafından desteklenmiştir.
Proje No: TÜBİTAK – TOG-TAG TARP-1825

Not: Bu tezde kullanılan özgün ve başka kaynaktan yapılan bildirişlerin, çizelge, şekil ve fotoğrafların kaynak gösterilmeden kullanımı, 5846 sayılı Fikir ve Sanat Eserleri Kanunundaki hükümlere tabidir.

ÖZ

DOKTORA TEZİ

ANTEPFISTIĞINDA FARKLI SU VE AZOT DÜZEYLERİNİN VERİM VE PERİYODİSİTE ÜZERİNE ETKİLERİ

YUSUF AYDIN

ÇUKUROVA ÜNİVERSİTESİ FEN BİLİMLERİ ENSTİTÜSÜ
TARIMSAL YAPILAR VE SULAMA ANABİLİM DALI

Danışman : Prof. Dr. Rıza KANBER
Yıl : 2004, Sayfa : 145

Jüri : Prof. Dr. Rıza KANBER
Prof. Dr. Cevat KIRDA
Prof. Dr. Ayzin KÜDEN
Doç.Dr. Sermet ÖNDER
Yrd. Doç. Dr. Mustafa ÜNLÜ

Bu çalışma 1999-2002 yılları arasında Antepfıstığı Araştırma Enstitüsü deneme alanında yürütülmüştür. Çalışmada farklı sulama aralıkları (SA_1=7 gün ve SA_2= 14 gün), azot dozları (N_g, N_o, N_1,N_2 ve N_3) ile bitki katsayıları (K_{cp1}=0.60 ve K_{cp2}=0.90) ele alınmıştır. Azot konuları, 0, 10, 15, 20 mg/l olarak düzenlenmiştir. N_g konusunda geleneksel olarak Şubat başında her bir ağaca 500/600/400 NPK (saf madde, g) olarak verilmiştir. Diğer gübre dozları fertigasyon tekniği kullanılarak sulama suyu ile birlikte uygulanmıştır. Sulama suyu açık su yüzeyi buharlaşmasına göre hesaplanmıştır. Sulama uygulamalarında arazinin % 30' u ıslatılmıştır.

Deneme yıllarında konulara farklı miktarlarda sulama suyu uygulanmıştır. Sulama suyu, sulama aralıklarına bağlı olarak 311-1164 mm arasında değişmiştir. Bitki su tüketimleri de deneme yılları boyunca farklı değerler almış, en yüksek ve en düşük su tüketimleri sırasıyla 1228-467.6 mm olarak bulunmuştur. En yüksek aylık bitki su tüketimi, $7N_390$ konusunda Temmuz ayında 290 mm ve bu ayda çim kıyas bitki su tüketimi 166 mm olarak hesaplanmıştır. Anılan konuya ilişkin K_c değeri, Temmuz ve Kasım aylarında, 1.51-0.39 olarak saptanmıştır. Buna karşılık olarak elde edilen en yüksek verim değerleri, 2000 ve 2001 yıllarında, 14 günlük sulama aralığı kullanılan konulardan sırasıyla 18.32 ve 12.02 kg/ağaç olarak elde edilmiştir.

Denemede kullanılan Uzun çeşidi, genetik özelliğine bağlı olarak kısmi periyodisite göstermiştir. Sulanan ve azot uygulanan konularda en yüksek periyodisite indeksi, 0.766; geleneksel konuda ise 0.627 olarak saptanmıştır.

Antepfıstığında verim tepki etmeni (K_y) 0.95 olarak hesaplanmıştır.

Anahtar Kelimeler: Fertigasyon, damla sulama, antepfıstığı, evapotranspirasyon, verim tepki etmeni

ABSTRACT
Ph.D. THESIS

THE EFFECT OF DIFFERENT IRRIGATION WATER AND NITROGEN LEVELS ON YIELD AND ALTERNATE BEARING IN PISTACHIO

YUSUF AYDIN

DEPARTMENT OF AGRICULTURAL STRUCTURES AND IRRIGATION
INSTITUTE OF NATUREL AND APPLIED SCIENCES
UNIVERSITY OF ÇUKUROVA

Supervisor : Prof. Dr. Rıza KANBER
Year : 2004, Page : 145

Jury : Prof. Dr. Rıza KANBER
Prof. Dr. Cevat KIRDA
Prof. Dr. Ayzin KÜDEN
Doç. Dr. Sermet ÖNDER
Yrd. Doç. Dr. Mustafa ÜNLÜ

This study was carried out between 1999 and 2002 at Pistachio Research Institute experimental field area. In the study, different irrigation intervals (I_1=7 days, I_2=14 days) and nitrogen concentrations (N_g,N_o,N_1,N_2,N_3) and different crop coefficients (K_{cp1}=0.60, K_{cp2}=0.90) were used. The nitrogen treatments were applied as 0, 10, 15, 20 mg/l. For N_g treatment, fertilizers were given in February under the tree canopy as a pure matter of NPK 500-600-400 gram per tree. The other fertilizer doses were given with irrigation water by using fertigation technology. The irrigation water was calculated according to free open water surface evaporation and wetted percent was assumed as % 30 of allocated full area per tree.

Different amount of irrigation water was applied during the experimental years. The amount of irrigation water was changed between 311-1164 mm according to irrigation interval used. Also the crop evapotranspiration was estimated with 1228-467.6 mm maximum and minimum values respectively. On the $7N_390$ treatment, the highest monthly evapotranspiration was calculated in July 290 mm and the references evapotranspiration was 166 mm at the same month. The maximum and minimum values of K_c for the same treatment in July and November were calculated as 1.51 and 0.39. According to the results, the highest yield values were optained in I_2 with 18.32 and 12.02 kg per tree in 2000 and 2001.

Uzun variety used in the treatment was showed relative periodisity due to its genetic charecterisation. The highest periyodisity index value was estimated as 0.766 for the treatments receiving nitrogen and irrigation water and as 0.627 for traditional treatment. The yield response factor was 0.95 on pistachio for 1999 and 2002.

Keywords : Fertigation, drip irrigation, *Pistacia vera L.*, evapotranspiration, yield response factor

TEŞEKKÜR

Akademik çalışmalarıma başladığım günden bu yana, çalışma konularımın seçimi, yürütülmesi, değerlendirilmesi ve yazım aşamalarında her zaman destek ve katkılarını yanımda gördüğüm, engin bilgilerinden yararlanmaya çalıştığım Sayın Hocam Prof. Dr. Rıza KANBER' e içtenlikle saygı ve teşekkürlerimi sunarım.

Çalışmanın planlanmasından tez yazım ve değerlendirme aşamasına kadar her türlü destek ve katkıyı sağlayan Yrd. Doç. Dr. Mustafa ÜNLÜ' ye;

Tez yazımı ve değerlendirilmesi aşamasında eleştiri ve katkılarıyla bana destek olan Antepfıstığı Araştırma Enstitüsünden Gıda Yüksek Mühendisi Abdullah YAMAN, Ziraat Yüksek Mühendisi Sibel AKTUĞ TAHTACI ve Ziraat Yüksek Mühendisi Serpil KARADAĞ' a;

Arazi çalışmaları boyunca bana yardımcı olan Antepfıstığı Araştırma Enstitüsü Tarım işçilerinden Ahmet BOZKURT, Mustafa KAYA, Hanifi POLAT ve Abdurrahman ÖZASLAN ile diğer emeği geçen bütün tarım işçisi arkadaşlarıma,

Doktora çalışmalarım boyunca her türlü zorluklara katlanarak, sabır ve özveriyle beni destekleyen eşim MİNE ile çocuklarım Uğurcan ve Nursevinç'e,

En içten dileklerimle teşekkür ederim.

Bu çalışmanın, antepfıstığı konusunda çalışan araştırmacılara yararlı olmasını dilerim.

Yusuf AYDIN

Haziran-2004

İÇİNDEKİLER SAYFA

ÖZ I
ABSTRACT II
TEŞEKKÜR III
İÇİNDEKİLER IV
ÇİZELGELER DİZİNİ VII
ŞEKİLLER DİZİNİ X
1.GİRİŞ 1
2. ÖNCEKİ ÇALIŞMALAR 5
2.1. Antepfıstığında Sulama 5
2.2. Antepfıstığında Gübreleme 11
3. MATERYAL ve YÖNTEM 18
3.1. Materyal 18
3.1.1 Araştırma Yeri 18
3.1.2. Toprak Özellikleri 18
3.1.3. İklim Özellikleri 21
3.1.4. Sulama Sistemi 25
3.1.5. Toprak İşleme 26
3.1.6. Bitki Çeşidi 26
3.1.7. Kullanılan Aygıtlar 27
3.2. Yöntem 28
3.2.1. Deneme Deseni ve Konular 28
3.2.2. Sulama ve Bitki Su Tüketimi 28
(i) Sulama 28
(ii) Gerçek Bitki Su Tüketimi 30

SAYFA

(iii) Kıyas Bitki Su Tüketimi 30

3.2.3. Toprak Su Kapsamının Belirlenmesi 31

3.2.4. Deneme Alanı Topraklarının İnfiltrasyon Özellikleri 33

3.2.5. Bitki Büyüme Değişkenlerinin Belirlenmesi 34

3.2.5.1. Ağacın fiziksel gelişimine ilişkin ölçmeler 34

3.2.5.2. Dal sayımları 35

3.2.5.3. Yaprak alan indeksi 35

3.2.5.4. Meyve gözleri (Karagöz sayımları) 36

3.2.5.5. Periyodisite indeksi 36

3.2.5.6. Kök yoğunluğunun belirlenmesi 37

3.2.5.7. Yaprak su potansiyelinin ölçülmesi 38

3.2.6. Toprak ve Bitki Analizleri 39

3.2.7. Bitki Katsayılarının Belirlenmesi (K_c) 39

3.2.8. Hasat 40

3.2.9. Su–Verim İlişkileri 40

3.2.9.1. Verim sonuçları 40

3.2.9.2. Su kullanma randımanları 41

3.2.9.3. Verim fonksiyonları 41

3.2.10. Kalite özellikleri 42

4. ARAŞTIRMA BULGULARI VE TARTIŞMA 43

4.1 Sulama ve Su Tüketimi (ET) 43

4.1.1. Sulama Durumu 43

4.1.2. Bitki Su Tüketimi 46

4.1.3. Bitki Su Tüketiminin Yersel Değişimi 49

4.2. Bitki Katsayıları (Kc) 59

4.3. Gübreleme 60

SAYFA

4.4. Bitki Büyüme Özellikleri.......................... 63
4.4.1. Karagöz Dökülme Oranı ve Verim-Karagöz İlişkisi.......................... 67
4.4.2. Antepfıstığında Periyodisite İndeksinin Belirlenmesi.......................... 71
4.4.3. Antepfıstığı Kök Yoğunluğunun Belirlenmesi.......................... 72
4.4.4. Yaprak Su Potansiyelinin Ölçülmesi.......................... 74
4.5. Verim Sonuçları.......................... 78
4.5.1. Sulama Suyu-Verim İlişkisi.......................... 83
4.5.2. Azot-Verim İlişkisi.......................... 84
4.6. Kalite Analizleri.......................... 86
4.7. Su Kullanma Randımanları.......................... 89
4.8. Verim Fonksiyonları (K_y) 93
5. SONUÇLAR VE ÖNERİLER.......................... 96
5.1. Sonuçlar.......................... 96
5.2. Öneriler.......................... 100
KAYNAKLAR.......................... 102
ÖZGEÇMİŞ.......................... 115
EKLER.......................... 116

ÇİZELGELER DİZİNİ

SAYFA NO

Çizelge 1.1. Dünya Antepfistığı Üreticisi Başlıca Ülkelerin Üretim Miktarları (Ton) 2
Çizelge 1.2. Türkiye Kabuklu ve İç Antepfistığı İhracatı (Ton-Dolar).......... 2
Çizelge 3.1. Deneme Alanı Topraklarının Bazı Fiziksel ve Kimyasal Özellikleri 19
Çizelge 3.2. Deneme Alanı Topraklarınının Profil Özellikleri.......... 19
Çizelge 3.3. Denemenin Yürütüldüğü Yıllara İlişkin Kimi İklimsel Veriler ve Uzun Yıllık Ortalama Değerler (1979-2000).......... 23
Çizelge 3.4. Deneme Alanındaki İki Kuyunun Sulama Suyu Analizi.......... 25
Çizelge 4.1. Konulara Uygulanan Sulama Suyu Miktarları (1999) 43
Çizelge 4.2. Konulara Uygulanan Sulama Suyu Miktarları (2000) 44
Çizelge 4.3. Konulara Uygulanan Sulama Suyu Miktarları (2001) 45
Çizelge 4.4. Konulara Uygulanan Sulama Suyu Miktarları (2002) 45
Çizelge 4.5. Bazı Konuların Mevsimlik Su Tüketimleri.......... 47
Çizelge 4.6. Deneme Konularına Verilen Azot, Fosfor ve Potasyum Miktarları (1999) 61
Çizelge 4.7. Deneme Konularına Verilen Azot, Fosfor ve Potasyum Miktarları (2000) 61
Çizelge 4.8. Deneme Konularına Verilen Azot, Fosfor ve Potasyum Miktarları (2001) 62
Çizelge 4.9. Deneme Konularına Verilen Azot, Fosfor ve Potasyum Miktarları (2002) 62
Çizelge 4.10. Antepfistığında Ölçülen/Kestirilen Bazı Büyüme Özellikleri (2001) 65
Çizelge 4.11. Antepfistığında Ölçülen/Kestirilen Bazı Büyüme Özellikleri (2002) 66
Çizelge 4.12. Uygulama Yıllarında Alınan Verim Değerleri (kg/ağaç) 80
Çizelge 4.13. Antepfistığında Meyve Kalite Özelliklerine İlişkin Sonuçlar.......... 87

SAYFA NO

Ek Çizelge 1. $7N_360$ Konusunda Su Bütçesi Yöntemine Göre Belirlenen Yığışımlı Su Tüketimi Değerleri (1999) 117

Ek Çizelge 2. $7N_390$ Konusunda Su Bütçesi Yöntemine Göre Belirlenen Yığışımlı Su Tüketimi Değerleri (1999) 118

Ek Çizelge 3. $14N_360$ Konusunda Su Bütçesi Yöntemine Göre Belirlenen Yığışımlı Su Tüketimi Değerleri (1999) 119

Ek Çizelge 4. $14N_390$ Konusunda Su Bütçesi Yöntemine Göre Belirlenen Yığışımlı Su Tüketimi Değerleri (1999) 119

Ek Çizelge 5. $7N_360$ Konusunda Su Bütçesi Yöntemine Göre Belirlenen Yığışımlı Su Tüketimi Değerleri (2000) 120

Ek Çizelge 6. $7N_390$ Konusunda Su Bütçesi Yöntemine Göre Belirlenen Yığışımlı Su Tüketimi Değerleri (2000) 121

Ek Çizelge 7. $14N_360$ Konusunda Su Bütçesi Yöntemine Göre Belirlenen Yığışımlı Su Tüketimi Değerleri (2000) 122

Ek Çizelge 8. $14N_390$ Konusunda Su Bütçesi Yöntemine Göre Belirlenen Yığışımlı Su Tüketimi Değerleri (2000) 122

Ek Çizelge 9. $7N_360$ Konusunda Su Bütçesi Yöntemine Göre Belirlenen Yığışımlı Su Tüketimi Değerleri (2001) 123

Ek Çizelge 10. $7N_390$ Konusunda Su Bütçesi Yöntemine Göre Belirlenen Yığışımlı Su Tüketimi Değerleri (2001) 124

Ek Çizelge 11. $14N_360$ Konusunda Su Bütçesi Yöntemine Göre Belirlenen Yığışımlı Su Tüketimi Değerleri (2001) 125

Ek Çizelge 12. $14N_390$ Konusunda Su Bütçesi Yöntemine Göre Belirlenen Yığışımlı Su Tüketimi Değerleri (2001) 125

Ek Çizelge 13. $7N_360$ Konusunda Su Bütçesi Yöntemine Göre Belirlenen Yığışımlı Su Tüketimi Değerleri (2002) 126

Ek Çizelge 14. $7N_390$ Konusunda Su Bütçesi Yöntemine Göre Belirlenen Yığışımlı Su Tüketimi Değerleri (2002) 127

Ek Çizelge 15. $14N_360$ Konusunda Su Bütçesi Yöntemine Göre Belirlenen

SAYFA NO

Yığışımlı Su Tüketimi Değerleri (2002) 128
Ek Çizelge 16. 14$N_3$90 Konusunda Su Bütçesi Yöntemine Göre Belirlenen Yığışımlı Su Tüketimi Değerleri (2002) 129
Ek Çizelge 17. Konuların Yıllara Göre Verim Değerleri (kg/ağaç) 130
Ek Çizelge 18. İstatistik Olarak Önemli Bulunan Yıllara İlişkin % Verim Değerleri.. 131
Ek Çizelge 19. Antepfistığı Verim Değerlerine İlişkin Varyans Analizi (1999) 132
Ek Çizelge 20. Antepfistığı Verim Değerlerine İlişkin Varyans Analizi (2000) 132
Ek Çizelge 21. Antepfistığı Verim Değerlerine İlişkin Varyans Analizi (2001) 133
Ek Çizelge 22. Antepfistığı Verim Değerlerine İlişkin Varyans Analizi (2002) 133
Ek Çizelge 23. Deneme Konularına İlişkin Meyve Kalite Özellikleri................ 134
Ek Çizelge 24. Çıtlama Oranlarının Varyans Analizleri (2000-2002) 135
Ek Çizelge 25. 100 Dane Ağırlıklarının Varyans Analizleri (2000-2002) 136
Ek Çizelge 26. İç Meyve Randımanlarına İlişkin Varyans Analizleri (2000-2002) 137
Ek Çizelge 27. Geleneksel Konu ile Diğer Konuların Meyve Kalite Özellikleri Yönünden Varyans Analizi ile Karşılaştırılması......... 138
Ek Çizelge 28. Deneme Konularının KDO-Verim İlişkisi (1999) 140
Ek Çizelge 29. Deneme Konularının KDO-Verim İlişkisi (2000) 141
Ek Çizelge 30. Deneme Konularının KDO-Verim İlişkisi (2001) 142
Ek Çizelge 31. Deneme Konularının KDO-Verim İlişkisi (2002) 143
Ek Çizelge 32. Oransal ET Açığı ile Oransal Verim Azalması Değerleri............. 143

ŞEKİLLER DİZİNİ

SAYFA NO

Şekil 3.1. Antepfıstığının yetiştiriciliğinin yapıldığı önemli İl'ler ve Gaziantep'in Güneydoğu Anadolu Proje alanındaki yeri.................... 18
Şekil 3.2. Deneme bahçesinin toprak profili.. 20
Şekil 3.3. Deneme bahçesi topraklarının jeomorfolojik özelliklerinin görünümü. 20
Şekil 3.4. Açık su yüzeyi buharlaşması (Class A Pan) ile yağış ilişkisi. 22
Şekil 3.5. Sulama sistemi denetim birimi (a); yan boru ve lateraller (b).. 25
Şekil 3.6. Antepfıstığı bahçesinde toprak işleme.. 26
Şekil 3.7. Antepfıstığı ağaçları (a) ve uzun çeşidinin meyveleri (b) 27
Şekil 3.8. Deneme bahçesinde blokların ve parsellerin şematik gösterimi........... 29
Şekil 3.9. Farklı toprak katmanlarında toprak su içeriğini belirlemek için oluşturulan kareler ağı.. 32
Şekil 3.10. Antepfıstığı deneme alanından elde edilen nötronmetre kalibrasyon eğrisi.. 32
Şekil 3.11. Deneme alanı topraklarının infiltrasyon tavrı. 33
Şekil 3.12. Deneme ağaçlarında kullanılan kimi büyüme değişkenlerinin şematik gösterimi... 34
Şekil 3.13. Yaprak alanı ile yaprak indeksi arasındaki ilişki................................ 35
Şekil 3.14. Denemede ele alınan su ve azot değişkenlerine göre oluşturulan kök örnekleme planı.. 37
Şekil 3.15. Denemede kullanılan yaprak su potansiyeli (YSP) ölçüm aleti.......... 38
Şekil 4.1. Sık sulanan ($7N_390$) konusuna ilişkin antepfıstığı su tüketimi ile çim kıyas su tüketimi değerlerinin zamansal değişimleri........................ 48
Şekil 4.2. Izgara köşelerindeki 100 cm' lik toprak katmanından $7N_390$ (a) ve "Geleneksel" (b) konularından evapotranspirasyon yoluyla oluşan toprak su kayıpları.. 52
Şekil 4.3. Izgara köşelerindeki 0-20 cm' lik toprak katmanından $7N_390$ (a) ve "Geleneksel" (b) konularından evapotranspirasyon

SAYFA NO

yoluyla oluşan toprak su kayıpları (b).. 53

Şekil 4.4. Izgara köşelerindeki 20-40 cm' lik toprak katmanından 7N$_3$90 (a) ve "Geleneksel" (b) konularından evapotranspirasyon yoluyla oluşan toprak su kayıpları 54

Şekil 4.5. Izgara köşelerindeki 40-60 cm' lik toprak katmanından 7N$_3$90 (a) ve "Geleneksel" (b) konularından evapotranspirasyon yoluyla oluşan toprak su kayıpları 55

Şekil 4.6. Izgara köşelerindeki 60-80 cm' lik toprak katmanından 7N$_3$90 (a) ve "Geleneksel" (b) konularından evapotranspirasyon yoluyla oluşan toprak su kayıpları 56

Şekil 4.7. Izgara köşelerindeki 80-100 cm' lik toprak katmanından 7N$_3$90 (a) ve "geleneksel" (b) konularından evapotranspirasyon yoluyla oluşan toprak su kayıpları 57

Şekil 4.8. Deneme alanı topraklarının 100 cm' lik kök derinliğinde farklı toprak katmanlarından 7N$_3$90 ve "Geleneksel" konularında meydana gelen ET kayıpları.. 58

Şekil 4.9. Antepfıstığında aylık ortalama K_c değerleri.. 59

Şekil 4.10. Deneme konularına ilişkin verim – KDO ilişkisi............................ 67

Şekil 4.11. Deneme konularına ilişkin KDO' nun yıllara göre değişimi.............. 68

Şekil 4.12. Deneme konularına ilişkin periyodisite indeksi değerleri............... 72

Şekil 4.13. Antepfıstığında farklı derinliklerdeki kök yoğunluğu.................... 73

Şekil 4.14 Sulama dönemi başında test konularına ilişkin YSP değerlerinin günlük değişimi.. 74

Şekil 4.15. Sulama dönemi içerisinde sulamadan önce (a) ve sulamadan sonra (b) YSP' de meydana gelen değişme................. 75

Şekil 4.16. Konuların sulama dönemi sonundaki YSP ' değerlerinin günlük değişimi 77

Şekil 4.17. Deneme yıllarında konulara ilişkin yığışımlı verim (a) farklı su, (b) farklı azot düzeyleri.. 82

Şekil 4.18. Antepfistığında sulama suyu verim ilişkisi (2000 ve 2002) 84
Şekil 4.19. Antepfistığında var ve yok yıllarında azot-verim ilişkisi (1999-2002) 85
Şekil 4.20. Kimi deneme konularına ilişkin toplam su kullanma randımanları.. 90
Şekil 4.21. Kimi konularda sulama suyu kullanma randımanlarının deneme yıllarında karşılaştırılması.. 91
Şekil 4.22. Konulara ilişkin sulama suyu kullanma oranlarının deneme yıllarında değişmesi... 93
Şekil 4.23.Antepfistığı için oransal su tüketimi azalışı oransal verim eksilişi arasındaki ilişkiler 94
Şekil 4.24. Antepfistığında periyodisite yıllarına (a) ve verim yıllarına (b) ilişkin verim tepki etmeni (K_y).. 95
Ek Şekil 1. Deneme konularının karagöz dökülme oranları (1999 ve 2000)........ 144
Ek Şekil 2. Deneme konularının karagöz dökülme oranları (2001 ve 2002)........ 145

1. GİRİŞ

Antepfıstığı yetiştiriciliği, Anadolu, İran ve Kuzey Suriye'de yüzyıllardır bilinmektedir. Tarihte, antepfıstığını Roma Kralı Vitellis'in I. Yüzyılda Suriye' den İtalya'ya getirdiği sanılmaktadır (Caruso, 2001; Özbek, 1978; Parfit, 1995). Daha sonra buradan Akdeniz adalarına, yaygın olarak İspanya, Güney Fransa ve Kuzey Afrika'ya taşınmıştır. Antepfıstığı yetiştiriciliğinin yaygın olarak yapıldığı yerlerden birisi, Ortadoğu bölgesidir (Özbek, 1978).

Antepfıstığı türlerinden olan *P. vera* L, botanik olarak *Anacardiaceae* familyasından olup, meyve ağacı ve süs bitkisi olarak değer bulan 11 türünün varlığı bilinmektedir (Özbek, 1978, Caruso, 2001; Hendricks ve Ferguson, 1995; Kaşka, 1998a). Antepfıstığı, Kuzey ve Güney Yarım Küreler'inin 30-45° paralelleri arasındaki uygun mikroklimalarda yetişmektedir (Ak ve ark., 1999). Özellikle, orta kısımları hariç Güneydoğu Anadolu bölgesi, İran' ın yaklaşık olarak tamamı ve Suriye'nin Güney kısımları çok zengin fıstık türlerine sahiptir (Kaşka, 1998 a,b). Antepfıstığının iki gen merkezi vardır: (1) Orta Asya gen Merkezi (Hindistan'ın Kuzeyi, Afganistan, Tacikistan, Pakistan) (2) Yakın Doğu Gen Merkezi (Anadolu, Kafkasya, İran ve Türkmenistan) (Ulusaraç 1993).

Türkiye, Kuzey Yarım Kürede ve Yakındoğu gen merkezi içerisinde bulunmaktadır. Bu anlamda, Güneydoğu Anadolu Bölgesi önemli bir konuma sahiptir. Antepfıstığının gen merkezlerinden olmasının yanı sıra, kendine özgü ekolojik yapısı bu bölgeyi, antepfıstığı yetiştiriciliğinde ön plana çıkarmıştır. Antepfıstığı anaç çeşitlerinden ***buttum***, daha çok Güneydoğu ve Doğu Anadolu'nun bazı yörelerinde; ***atlantik sakızı*** ise Akdeniz, İç Anadolu ve Ege bölgelerinde yaygın olarak bulunmaktadır (Atlı ve ark., 1998).

Dünya antepfıstığı üretiminde beş önemli üretici ülke vardır. Bunlar sırasıyla İran, ABD, Türkiye, Suriye ve Çin' dir (Çizelge 1.1). Ülkemiz, antepfıstığı üreticisi olan ülkeler arasında halen üçüncü sırada bulunmaktadır.

Çizelge 1.1. Dünya Antepfıstığı Üreticisi Başlıca Ülkelerin Üretim Miktarları (ton) FAO, 2003 (www.fao.org)

Yıllar	İran	ABD	Türkiye	Suriye	Çin	Yunanistan	Afganistan	İtalya	Tunus	Dünya
1993	229 332	68 950	50 000	13 700	24 000	4 786	2 200	1 799	900	397 080
1994	195 000	58 500	40 000	14 925	25 000	5 573	2 300	240	900	342 781
1995	239 000	67 130	36 000	14 500	25 000	5 400	2 400	2 200	900	393 107
1996	282 000	47 630	60 000	18 000	28 000	6 700	2 500	100	1 000	428 702
1997	111 916	81 650	70 000	29 428	30 000	3 600	2 600	5 000	1 150	336 251
1998	313 957	85 280	35 000	35 684	32 000	5 000	4 000	100	1 200	512 221
1999	200 000	49 900	40 000	33 567	32 000	5 000	2 800	100	1 200	292 391
2000	200 000	94 170	75 000	33 000	32 000	6 500	2 800	4 500	1 200	449 170
2001	112 432	73 030	30 000	37 436	26 000	7 500	0	2 500	1 100	294 425
2002	300 000	136 078	40 000	52 840	26 000	8 500	3 000	2 500	800	571 150

İran, antepfıstığı üretiminde olduğu gibi, dışsatımında da % 50 payla birinci sırada yer almaktadır (FAO, 2003 Web sayfası; GAİB, 2003). Ülkemiz ise dünya antepfıstığı dış satımının ancak % 3' nü karşılamaktadır (Çizelge 1.2) (Anonim, 2002). Türkiye'nin toplam antepfıstığı dışsatımının küçük bir bölümü (%3'ü) Güneydoğu Anadolu Bölgesinden karşılanmaktadır.

Çizelge 1.2. Türkiye Kabuklu ve İç Antepfıstığı İhracatı (ton-dolar) (GAİB, 2003. Sezon aralığı 1 Ağustos-31 Temmuz)

Yıllar	Kabuklu Antepfıstığı		İç Antepfıstığı		
	Miktar (ton)	Değer ($ 000)	Miktar (ton)	Değer ($ 000)	Toplam ($ 000)
1996/1997	2 190	8 220	366	2 508	10 728
1997/1998	3 621	14 004	822	4 337	18 341
1998/1999	1 360	5 730	144	1 252	6 982
1999/2000	959	4 949	64	631	5 580
2000/2001	1 894	6 703	1 342	5 995	12 698
2001/2002	3 518	10 163	2 012	9 253	19 416
2002/2003	1 126	4 498	318	2 800	7 298
TOPLAM	14 668	54 267	5 068	26 776	81 043

Çizelgelerden görüldüğü gibi, hem Dünya hem de Türkiye'de antepfıstığı üretimi yıldan yıla değişme göstermektedir. Söz konusu verim dalgalanması, temelde

üretici ülkelerin doğrudan yetiştiricilik koşulları ile ilişkilidir. Türkiye' de ise dalgalanmanın asıl nedeni, ilkel yetiştiricilik koşullarıyla birlikte periyodisitedir. Genel anlamda periyodisite, antepfıstığı ağaçlarının bir yıl ürün vererek ertesi yıl hiç ürün vermemesi veya öncekinden daha az ürün vermesi şeklinde tanımlanmaktadır.

Türkiye, antepfıstığının gen merkezi ve daha fazla ağaç sayısına sahip olmasına karşın, elde edilen düşük verim, üretimin kuru koşullarda yapılmasından ve periyodisite eğilimi fazla olan çeşitlerin kullanılmasından kaynaklanmaktadır (Tekin,1993). Ülkemizde kurulu bahçelerde daha çok mutlak periyodisite gösteren, yerli çeşitler kullanıldığından verim dalgalanması daha da şiddetli olmaktadır.

Antepfıstığı üretiminin yoğun olarak yapıldığı Türkiye'de, son istatistiklere göre, 56 il'de yetiştiricilik yapılmasına karşın, üretimin % 94'ünü Güneydoğu Anadolu Bölgesi karşılamaktadır (Tekin ve ark., 2001). Anılan bölgedeki antepfıstığı bahçelerinin kurulduğu alanlar, coğrafik konumları gereği engebeli ve yüzlek toprak derinliklerine sahip olduklarından; bu durum üreticileri kuru koşullarda yetiştiriciliğe zorlamaktadır. Değinilen durumun doğal sonucu olarak, antepfıstığı verimi oldukça düşüktür ve istenen düzeyde artmamıştır. Türkiye'de ağaç başına ortalama verim, yalnızca 1.4 kg iken; sulama ve fertigasyon uygulamaları gibi tekniklerin kullanıldığı ABD'de, üretime 1960'lı yıllardan sonra başlanmış olmasına karşın, 16-18 kg arasındadır (Tekin ve ark., 1990; Ak ve ark., 1999). Bu hızlı gelişme, üretimin, suya tepkileri önceden belirlenen uygun anaçlarla ve sulu koşullarda yapılmasına, nitelikli ve verimli çeşit-anaç bileşiminin kullanılmasına bağlanmaktadır.

Antepfıstığı kuraklığa dayanıklı ve gelişmesi çok yavaş olduğundan, Türkiye'de üreticiler bitkiyi kurak, taşlık, kayalık ve yüksek kireç içeren topraklarda yetiştirmektedirler (Kaşka, 2001). Antepfıstığı her türlü toprakta yetişebilmesine karşın, daha çok derin, kireçli, kumlu-tınlı toprakları yeğlemektedir (Crane, 1977). Goldhamer (1995), iyi bir yetiştiricilik için derin, verimli, iyi drene olabilen toprakları salık vermiştir. Uygun özellikler taşımayan topraklar ve kültürel işlemler, üretimde önemli düşmelere neden olmaktadır

Türkiye'de antepfıstığı veriminde görülen dalgalanma ve düşüklük, yukarıda da değinildiği gibi, periyodisite ve döllenme biyolojisi ile ilgili sorunlardan da

kaynaklanmaktadır. Ancak sulama ve gübreleme gibi üretim teknikleri kullanıldığında, periyodisite tamamen ortadan kaldırılmasa bile, şiddeti azaltılabilmektedir (Kanber ve ark., 1990 ve 1993). Bu durumun, sulama ile meyve iriliği, çıtlama, yaprak büyüklüğü, sürgün sayısı ve uzunluğunun artmasından, boş meyve oranının azalmasından kaynaklandığı sanılmaktadır (Kanber ve ark., 1990).

Antepfistığında döllenme biyolojisi ile iklim etmenleri arasında çok yakın bir ilişki vardır. Sıcaklık, yağış, sis ve rüzgar antepfistığında döllenme noksanlığına neden olarak; boş meyve oluşumunu artırmaktadır. Çiçeklenme zamanında aşırı sıcaklıklar silkmeye, düşük sıcaklıklar ise salkımların zararlanmasına neden olmaktadır (Atlı ve Açar, 2001). Benzer şekilde yağmur ve sis, çiçek tozu hareketini yavaşlatarak, döllenmeyi olumsuz etkilemektedir.

Gerek periyodisitenin azaltılması, gerekse yüksek verim için yaşamsal önemde olan antepfistığında sulama ve gübreleme konusunda, Türkiye'de yapılan çalışma sayısı yeterli düzeyde değildir, hatta çok azdır. Çalışmaların hemen tümünde, sulamanın antepfistığı için kaçınılmaz bir zorunluluk olduğu ortaya çıkmıştır (Bilgen, 1973 ve 1982; Kuru, 1992; Kanber ve ark., 1990 ve 2000). Ancak sulama ve gübrelemenin periyodisite üzerindeki etkisi tam olarak açıklanamamıştır (Ayfer, 1990). Kimi araştırmada, sulamanın etkisi açık olarak ortaya çıkarken (Kanber ve ark. 1990), diğerlerinde ise değinilen etki belirsizliğini korumaktadır (Bilgen, 1973 ve 1982). Açıklanan konu, gelecekte antepfistığı ekiliş ve sulamalarının yaygınlaşacağı beklenilen GAP sulanır alanları için çok büyük önem taşımaktadır.

Çalışmada; Antepfistığı–su ilişkisini incelemek, en uygun sulama / fertigasyon programlarını belirlemek amaçlanmıştır. Ayrıca, damla sulama sistemleri aracılığıyla yapılan fertigasyon tekniğinin antepfistığında uygulanma olanaklarını araştırmak hedeflenmiştir. Bu amaçla, Gaziantep yöresinde antepfistığında farklı su ve azot düzeyi kullanılarak verim ve periyodisite ilişkileri irdelenmeye çalışılmıştır. Öte yandan, yetiştiricilere büyük olanaklar sağlayacağı düşünülen fertigasyon tekniği kullanılarak, gübreleme programlarının eldesi ve kullanıma sunulması, bu çalışmanın bir diğer amacını oluşturmaktadır.

2. ÖNCEKİ ÇALIŞMALAR

2.1 Antepfıstığında Sulama

Antepfıstığı ağaçları, kuraklığa dayanıklı olduğundan çok az miktarda su ile yaşamını ve verimliliğini devam ettirebilir. Ancak, kuraklığa dayanıklı olması, optimum bitki gelişimi için çok az bir suyun yeterli olduğu anlamına gelmez (Goldhamer, 1995). Nitekim ABD' de antepfıstığı sulanan alanlarda, pamuk ve turunçgillerle yan yana yetiştirilmektedir (Schwankl, 1995).

Goldhamer ve ark. (1985), antepfıstığının şiddetli su stresine karşı gösterdiği fizyolojik tepkiyi belirlemişlerdir. Çalışmada, A sınıfı buharlaşma kabından meydana gelen buharlaşmanın % 0-25-50-70 ve 100'ü kadar sulama suyu uygulandığında ürün niteliğinde önemli oranda düşmeler olduğunu saptamışlardır. Buna ek olarak, aynı araştırmacılar, yağmurlama sistemiyle sulanan antepfıstığı için sulama programlarında kullanılmak üzere bir bitki-pan katsayısı geliştirmişlerdir.

Ülkemizde sulama konusunda ilk çalışma, 1973 yılında Antepfıstığı Araştırma Enstitüsünde yapılmış (Bilgen, 1973 ve 1982; Kuru, 1992) ve halen devam etmektedir (Kanber ve ark., 2000). Antepfıstığı yetişen topraklarda, 90-180 cm derinliklerdeki yararlı suyun % 50'si tüketildiğinde sulama yapılmasıyla sürgün uzunluğunda ve 100 dane ağırlığında artışlar meydana gelmiştir (Bilgen, 1982). Benzer olarak, Monastra ve ark. (1997), farklı su miktarlarının ağacın vegetatif gelişmesi, olgunlaşması ve verimi üzerine etkilerini belirlemek amacıyla yaptıkları çalışmada, sulamanın vegetatif gelişmeyi artırdığı ve verimi olumlu biçimde etkilediğini belirlemişlerdir. Buna ek olarak, gövde gelişimi, yaprak alan indeksi (LAI) ve sürgünlerdeki meyve gözü sayısı ve gözlerin olgunlaşması üzerine olumlu etki yaptığı ortaya çıkmıştır. Fakat aşırı sulama, çiçek başına meyve tutumunu olumsuz etkilemiştir.

Kanber ve ark. (1990), Türkiye' de GAP bölgesinde 1974–1985 yılları arasında antepfıstığında sulama ilişkilerini incelemişlerdir. Çalışmada, yüzey sulama yöntemlerinden tava sulama kullanılmıştır. Özellikle verim yılında yapılan sulama

uygulamaları, meyve verimini artırmıştır. Sulama aralığının 20 gün olduğu durumda verim yılında ağaç başına, 57.1 kg ürün alınırken, aynı dönemde sulamanın yapılmadığı kontrol konusunda ağaç başına 34.5 kg meyve alınmıştır. Periyodisite yılında sulanmayan konuda 17.3 kg/ağaç ürün alınırken, sulanan konuda ağaç başına 40.3 kg gibi daha yüksek verim alınmıştır. Sulama, meyve kalitesi üzerinde etkili olmamıştır. Normal verim yılında mevsimlik bitki su tüketimi 803 mm olmuştur. En yüksek aylık bitki su tüketimi 205 mm ile Ağustos ayında gerçekleşirken günlük su tüketimi 6.6 mm olmuştur. Kaliforniya' da ise antepfistığının mevsimlik su tüketimi, 1017 mm olarak bulunmuştur (Goldhamer ve ark., 1985). Kanber ve ark. (2000)' nın yaptıkları bir başka çalışmada, sulanan ve sulanmayan antepfistığı ağaçlarında fotosentez hızları ölçülmüş, sulanan ağaçlarda fotosentez hızı % 32 daha yüksek bulunmuştur. Sulanan ağaç daha fazla karbonhidrat biriktirerek meyve gözleri dökümünün engellenmesine olumlu etki yapmıştır.

Bilgel ve ark. (1999) tarafından Harran Ovası koşullarında antepfistığının (Siirt çeşidi) su tüketimi ve sulama programının belirlenmesine yönelik yaptıkları çalışmada; antepfistığı ağacının 45 gün aralıklarla 3 kez sulanması gerektiğini, sulamaların Haziran ayı başından Eylül ayı başına değin olan aralıkta verilmesi gerektiğini belirtmişlerdir. Araştırmacılar, antepfistığı mevsimlik sulama suyu gereksinimini 450 mm, su tüketimini ise 600 mm olarak bulmuşlardır.

Kaliforniya koşullarında antepfistığı anaçlarının tuza dayanımları konusunda yapılan çalışmada, 8 dS/m tuz içeren sulama suyu, verim ve ağaç gelişimini olumsuz biçimde etkilememiştir. Ardışık 3 sulama döneminde, 1997 yılında 12 dS/m tuzluluk düzeyindeki sulama suyu ile yapılan uygulamalarda, ağaç gelişiminde UCB1 anacında %19 düzeyinde gerileme gözlenirken, Atlantica anacında bu oran %14 olarak bulunmuştur (Ferguson ve ark., 2001). PG1 ve PGII anaçlarında herhangi bir gelişme geriliği görülmemiştir. Araştırmada, 1998 yılında kullanılan 4 anaç çeşidinde gelişme, sırasıyla % 49, % 19, % 18 ve % 14 oranlarında gerilemiştir.

Metheney ve ark. (1998), verim çağındaki antepfistığı ağaçlarında, drenaj sularının potansiyel kullanılabilirliğini araştırmışlardır. Elektriksel iletkenliğin 8 dS/m'ye kadar sulama sularının, ağaç gelişiminde, bir dönem kullanılabileceğini; bunun bitki gelişiminde ve verimde herhangi bir azalmaya neden olmayacağını

belirtmişlerdir. Bununla beraber, aynı düzeydeki sulama suyunun ardışık iki sezon kullanılmasıyla ağaç gelişimi ve verim üzerinde olumsuz etki yapacağını açıklamışlardır.

Sera koşullarında yapılan bir başka çalışmada, antepfıstığında sulama suyu tuz düzeyleri, çeşitlere bağlı olarak, farklı etkiler göstermiştir (Sepeaskhah ve Maftoun, 1981). Buna göre, kullanılabilir kapasitenin %50 düzeylerinde ve iyi nitelikli sulama suyu uygulamasıyla yüksek verim alındığı, çeşitlere bağlı olarak 12-15 dS/m tuz düzeylerinde büyümenin durduğu saptanmıştır. Sulama aralığının 7 gün olduğu ve 4.5 dS/m tuzluluk düzeyine sahip sulama suyunun kullanılmasıyla, bitki büyümesinde ortalama % 38'lik bir gerileme gözlenmiştir. Bununla beraber, araştırmacılar, yüksek verim alabilmek için sulama uygulamalarında, yararlanılabilir toprak su içeriğinin en fazla % 50'ye kadar düşmesine izin verilmesi gerektiğini açıklamışlardır. Sulamanın; sürgün uzunluğu, yaprak alanı, meyve iriliği ve ağırlığını etkilediğini belirtmektedirler.

Sepeaskhah ve Maftoun (1982), sulama aralığı ve sulama suyu tuzluluk düzeylerinin, antepfıstığının kimyasal yapısı üzerine etkilerini belirlemek amacıyla sera koşullarında yaptıkları çalışmada, bazı bitki besin maddeleri ve suyun topraktan alımları ve dağılımlarının olumsuz yönde etkilendiğini belirlemişlerdir. Sulama suyu tuzluluk düzeyinin artmasıyla, Cl ve Na iyonları birikiminde artışlar olduğu; sık sulamaların (3 gün) iyon birikimini daha fazla artırdığını saptamışlardır.

Antepfıstığı yetiştiriciliğinde karşılaşılan önemli sorunlardan olan verim düşüklüğünün nedenlerini, son yıllarda bazı araştırmacılar, su stresine bağlamaktadırlar. Su stresinin verimi yaklaşık olarak, yarı yarıya azalttığı, meyve niteliğinde bozulmalara neden olduğu Kanber ve ark. (1993) tarafından rapor edilmiştir. Benzer olarak, Ak ve Kaşka (1992), periyodisiteyi mutlak ve oransal olmak üzere iki sınıfa ayırarak, Kırmızı çeşidinin mutlak periyodisite, Ohadi ile Siirt çeşitlerinin ise oransal periyodisite gösterdiklerini belirtmişlerdir. Sulama ve gübreleme uygulamalarının periyodisitenin önlenmesinde etkili yöntemler olduğunu, toprakta yeteri kadar yararlanılabilir su olması koşulunda antepfıstığının kuvvetli sürgün verdiğini, daha geniş yaprak alanı oluşturduğunu, bunun da karbonhidrat birikimini artırarak meyve veriminin artmasına yol açacağını savlamışlardır.

Araştırmacılar, büyüme düzenleyicilerinin periyodisite üzerinde etkili olmadığını açıklamışlardır. Ancak, Lovatt ve Ferguson (1997), periyodisiteye neden olan göz dökülmesini engellemek için, % 0.25'lik azotu 6 mg Benzyladenin ile birlikte yapraktan uygulayarak, meyve gözü tutumunu iki yıl içerisinde 2-3 katına çıkardıklarını açıklamışlardır.

Geurts (1982), antepfıstığı üretiminde düzensiz verimin yetiştiricilikte karşılaşılan önemli bir sorun olduğunu, verimde gözlenen kararsızlığın uzun süreli su stresinin yanı sıra yetiştiricilikte geleneksel yöntemlerin kullanılması, yetersiz tozlanma, gübreleme ve sulamanın yapılmaması ile ilkel kültürel yöntemler olduğunu açıklamıştır. Antepfıstığının ikinci önemli sorunu olan periyodisitenin, uygun yetiştiricilik teknikleriyle birlikte sulama uygulamalarıyla en aza indirilebileceği Kanber ve ark. (1990) tarafından belirtilmiştir. Bununla beraber, bazı araştırmacılar antepfıstığında görülen periyodisitenin, sulamanın yapılmaması ve kültürel önlemlerin yetersizliğinin yanı sıra, bitki dokularında azot birikiminin azlığından kaynaklandığına bağlamaktadırlar (Ak ve Kaşka,1992).

Sykes (1975), Türkiye' de antepfıstığı ekilişlerinin genişlemesinin, yüksek ve düşük hava sıcaklıkları ile düşük yıllık yağış gibi iki temel öge tarafından sınırlandığını yazmıştır. Yıllık yağışın 400 mm' nin altında olduğu kurak yıllarda antepfıstığının, uzun süren su eksikliği nedeniyle yapraklarını döktüğü gözlenmiştir. Belirli bir yılda meydana gelen yaprak dökülmesi, ardıl yıllarda meyve gözü (karagöz) gelişimini engellemekte ve bu şekilde verimin düşmesine neden olmaktadır.

Spiegel-Roy ve ark. (1977), antepfıstığının düşük toprak su koşullarına tepkisini belirlemek amacıyla yaptıkları çalışmada, verim çağındaki antepfıstığı bahçelerinde toprak su içeriğinin solma noktasının altında olduğu kurak yıllarda kök hareketinin 4-5 hafta tam olarak durduğu durumlarda bile verimde herhangi bir azalma olmadığını açıklamışlardır. Bu durum, sulamanın antepfıstığı meyve verimini artırdığı anlamına gelir. Firuzeh ve Ludders (1978), antepfıstığının genç döneminde (5–7 yaş) tuza oldukça dayanıklı, fakat don ve su eksikliğine karşı çok duyarlı olduğunu belirtmişlerdir. Kesin olmamakla birlikte, uzun dönemli su eksikliğine uyum göstermiş, çok yaşlı (30-40 yaş) antepfıstığı ağaçlarının sulama

uygulamalarından yararlanabileceğini, kök sistemi gelişmiş ağaçların daha derin toprak katmanlarındaki suyu kullanabileceğini belirtmişlerdir.

De Palma ve Novello, (1997), Güney İtalya'da, *P. integerrima* üzerine aşılı Larnaka cv. çeşidine farklı sulama suyu miktarlarının etkileri üzerinde çalışmışlardır. Bitki Su Tüketiminin (ETc) % 0-25-50-75'i kadar sulama suyu (0-1500-3000-4500 m^3 ha^{-1} $yıl^{-1}$) damla sulama yöntemi ile uygulanmıştır. Fizyolojik incelemelere, net CO_2 özümlemesi, stomatal iletkenlik, transpirasyon, verim miktarı ve olgun yapraklarda su kullanım randımanı, ölçülerek başlanmıştır. Ayrıca, sürgün gelişimi, yaprak sayısı ve sürgün başına yaprak alanı, gövde ve yaprak su potansiyeli ölçümleri yapılmıştır. Fotosentez, su kullanım randımanı ve gövde su potansiyeli, 3000 m^3 ha^{-1} sulamaya dek artmaya devam etmiş, en yüksek sulama düzeyinde azalmışlardır.

Goldhamer ve ark. (1997), sulama yönteminin ve sulama suyunun *Verticillium* ile yüksek oranda bulaşık antepfıstığı bahçelerinde, kök dağılımı üzerine etkilerini incelemişlerdir. İnceleme sonunda, damla sistemiyle sulanan toprağın ilk 15 cm' lik katmanında hacimsel toprak su içeriği ortalama % 3.7 olarak bulunmasına karşın, düşük hacimli yağmurlama sulamada (LVS) % 10.6 olduğu saptanmıştır. Gömülü damla sulama sisteminde ilk 30 cm' de toprak su dağılımı, serili damla sistemi ile aynı özellik göstermiştir. Toprağın 30 cm derinliğine gömülü damla sulamada toprak su dağılımı, serili damlanınki ile aynı bulunmuştur. Damla sistemi ile sulanan konularda en yüksek toprak su düzeyi, düşük hacimli yağmurlama (LVS), 30 cm'ye gömülü damla ve serili damla yöntemlerine kıyasla, 60 cm' ye gömülü damla sisteminden elde edilmiştir. Toprak profilinde 40 cm'nin üzerinde kök bulunmazken, düşük hacimli yağmurlama sisteminde apaçık olarak görülmektedir. Kuru olan üst toprak profilinde kök geometrisi, net olarak toprak altı sulamadan etkilenmektedir.

Sulama suyunun kısıtlı olması durumlarında, sudan artırım sağlamak için "Denetimli Eksik Sulama" (regulated deficit irrigation) yöntemleri kullanılabilir. Goldhamer ve Beede (1993), verim çağındaki antepfıstığında kısıntılı sulama koşullarının etkisini belirlemek amacıyla yaptığı çalışmada, bitkinin dış kabuğunun

büyümeye başladığı dönem ile meyve içinin hızlı gelişmeye başladığı dönemler arasında su stresine dirençli olduğunu saptamışlardır. Bu dönemde sulama suyundan % 50 kısıntı yapılabileceği ancak, hasada yakın dönemde su stresinden kaçınmak gerektiğini açıklamışlardır. Bu dönemde uygulanacak su stresinin, doğal çıtlaklığı ve meyvenin hasat edilebilirliğini azalttığı gibi kopma direncini artırdığını belirtmişlerdir. Buradan, fidan dönemindeki antepfıstığı ağaçlarında ve yaprak gelişimi dönemlerinde su stresinden kaçınılması gerektiği söylenebilir.

Freeman ve Ferguson, (1995) ile Ak ve ark. (1999), Ağustos ortasından Eylül başına kadar yapılan yetersiz sulamanın, meyve çıtlama oranını önemli oranda azaltırken ve boş meyve oluşumunu artıracağını, bu nedenle, antepfıstığı ağacının belirtilen dönem içerisinde, su azlığından kaçınılması gerektiğini açıklamışlardır.

Antepfıstığının sulanmasında hemen her yöntem kullanılabilir. Düzgün olmayan engebeli arazi koşullarında, yağmurlama ve damla gibi, basınçlı sulama sistemleri kullanılabilir. Daha düz arazi koşullarında ise basınçlı sistemlerle birlikte diğer yöntemler de, toprak yapısı, su kaynağının durumu, su iletim ve uygulama maliyeti gibi öğeler göz önüne alınarak dikkate alınabilir. Ancak, hangi sistem olursa olsun, sulama yapılırken suyun gövdeye kesinlikle değmemesi gerekir (Maranto ve Crane, 1982).

Antepfıstığı sulamasında damla sulamanın kullanılabileceğini belirten Phene ve ark. (1985), kısıtlı sulama koşullarının, verimi önemli ölçüde etkilediğini belirtmişlerdir. Sulama suyunda yapılacak % 25'lik bir kısıntı, verim üzerinde herhangi bir olumsuz etki oluşturmazken, % 50 ve % 75'lik su kısıntısının, verim ve meyve niteliğinde önemli oranda düşmelere neden olduğunu açıklamışlardır.

Hendricks ve Ferguson, (1995) antepfıstığı üretiminde sulamanın, özellikle sulama suyu miktarı, zamanı ve uygulama yönteminin çok önemli olduğunu; genç antepfıstığı ağaçlarının gelişiminde, toprak kökenli ve havalanmadan kaynaklanan hastalıkların ortaya çıkmasında, verim ve kalite üzerinde büyük etkisinin olduğunu yazmışlardır.

İran' da antepfıstığı sulamasında, genellikle karık veya göllendirmeli karık yöntemleri kullanılmaktadır. Yetişkin ağaçlarda sulama suyunun, ağaç gövdesine

değmesi, toprak seddeleri yapılarak engellenir. Bu uygulamanın, ağacın *gomosis disse* hastalığı ile bulaşmasına engel olduğu kaydedilmiştir (Sheibani, 1994).

İran' da, antepfıstığı bahçesi kurulduktan sonra, ilk 3 yıl içerisinde sulamalar genellikle, 7-15 gün aralıklarla uygulanmaktadır. Gelişmenin 4. ve 5. yıllarında 20 güne çıkarılır ve bu durum ağaç büyüyünceye kadar devam eder. Sulama aralığı 30-45 güne kadar ulaşabilmektedir (Sepaskhah ve Maftoun, 1981).

Mc Donald ve ark. (1992), farklı sulama sistemlerinin ve su uygulama sıklığının, genç antepfıstığı bahçelerinde kök dağılımı ve olası bulaşıcı hastalıklar üzerine etkilerini incelemişlerdir. Mini yağmurlama, damla sistemine göre kök yoğunluğu ve mikrosclerotial populasyonu daha fazla artırmıştır. Mikrosclerotial populasyondaki farklılıkların, toprak yüzeyinin ıslanma alanı ve yabancı ot gelişimindeki ayrımlardan ileri geldiğini belirlemişlerdir.

Michialidies ve Morgan (1991), kabul edilebilir bir sulama işletim tekniğinin antepfıstığı bahçelerinde hastalıkların denetiminde etkili rol oynayacağını açıklamışlardır. Iran - Kerman bölgesinde yaklaşık 210 000 hektarlık alanda antepfıstığı yetiştiriciliğinin yapıldığını belirten Sheibani (1994), yüzey sulama yöntemleri ile yılda 700-1400 mm, damla sulama ile 250 mm sulama suyu uygulandığını belirtmiştir.

2.2 Antepfıstığında Gübreleme

Antepfıstığında verim düşüklüğü ve periyodisite şiddetinin azaltılmasında en önemli gelişim etmenlerinin, su ve gübreleme olduğu bilinmektedir. Bunun için yeni sulama ve gübreleme tekniklerine gereksinim vardır. Bu amaçla gübrelerin sulama suyu ile birlikte ve ona karıştırılarak uygulanması tekniği giderek yaygınlık kazanmaktadır. Tarımsal kimyasalların kullanılması durumunda *kemigasyon*, sulama suyuna gübrelerin karıştırılarak kullanılması tekniğine de *fertigasyon* denilmektedir (Hamdy, 1995; Papadopoulos,1995). Bu yöntemle bitki kök bölgesi toprağında su ve besin maddesi düzeylerini optimum düzeyde tutmak olanaklı hale gelmiş, gübrelerden kaynaklanan kök bölgesi tuzluluğu denetim altına alınabildiği gibi tuza

duyarlı bitkilerin yetiştirilmesi sağlanmıştır. Su ve gübrenin birlikte uygulanması olarak bilinen fertigasyon tekniği, son yıllarda, bilimsel araştırmalarda çalışma konusu olmaya başlamıştır (Kırda ve ark.,1997).

Brown (1998), antepfıstığında besin noksanlıklarının belirlenmesi üzerine yaptıkları çalışmada, normal büyüme ve verimlilik için temel olarak 14 elemente gerek duyduğunu ve bunların makro elementler (N, P, K, Ca, Mg, S) ile mikro elementler (Fe, Mn, Cl, B, Cu, Zn, Ni, Mo) olduğunu yazmıştır. Araştırmacı antepfıstığı yapraklarında besin kapsamlarının sınır değerleri (CV-verimin % 95 ve simptonların görülmediği kritik değer) ile buna karşılık önerilen değerleri vermiştir.

Güneydoğu Anadolu Bölgesinin antepfıstığı yetiştirilen alanlarında, antepfıstığı besin elementleri kapsamlarının belirlenmesi için 3 ayrı yörede yapılan çalışmalarda, toprağın pH değerlerinin 8.1-8.7 arasında; organik madde, yararlı fosfor yönünden önemli oranda, potasyumca kısmen noksanlık olduğu, Tekin ve ark. (1990) tarafından belirtilmiştir. Benzer bir başka çalışmada, gerek antepfıstığı bahçe toprağının ve gerekse ağacının makro besin elementleri, azot ve fosfor yönünden yetersizliği belirtilirken (Aydeniz,1990); bitki besin maddelerinin eksikliği yanında, alımını güçleştiren sığ toprak profili, yüksek kil ve kireç, düşük organik madde içeriği, alkalin reaksiyon ve yetersiz nem düzeyinin, mevcut bitki besininden ya da kullanılan gübreden yeterince yararlanamadığı, aynı araştırmacı tarafından açıklanmıştır.

Muntubani ve ark. (1997), 25 yaşlarındaki Kerman çeşidi antepfıstığında yaptıkları çalışmada, periyodisite yılındaki meyvesiz ağaçların yapraklarının meyveli ağaçlara göre, tam çiçeklenmeden 30 gün sonra yaklaşık iki kat daha fazla geliştiğini; bu durumun, meyve oluşumunun etkisi sonucunda meydana geldiğini belirtmektedirler. Araştırmacılar, verim yılındaki ağaçlarda yıllık sürgünlerin, toplam yaprak alanının, meyvesiz ağaçtan daha büyük olduğunu; bunun meyveli ağaçların sürgünlerinde oluşan 8 yaprağa karşın, meyvesiz ağaçların sürgünlerinde 6 adet yaprak oluşmasından ileri geldiğini açıklamışlardır. Ayrıca, verim yılındaki ağaçların meyve gözlerinin, meyvesiz ağaçlara oranla, daha fazla döküldüğünü kaydetmişlerdir.

Ak ve ark. (1992), sürgünlerde azot birikiminin azalması ile sulama ve gübrelemenin yapılmamasının, periyodisitenin şiddetini arttıracağını açıklamışlardır. Meyveli ağaçlarda, çiçeklenmeden 103 gün sonra, 0.69 mg' ın üzerinde N içeren meyve gözlerinin dökülmediklerini, bu değerin altına düştüğünde ise döküldüklerini belirten Porlingis (1974), meyvesiz ağaçların meyve gözlerinin 0.73 mg N içerdiklerini ve dökülmediklerini açıklamıştır. Weinbaum ve Muraoka (1989), verim çağındaki antepfıstığı yapraklarından % 1.8 den daha az N içerenlerin, zamanından önce sararıp döküldüklerini; böyle bir ağacın yaprakları tarafından 151.3 g N tüketildiğini yazmışlardır. Aynı araştırmacılar, 25 kg meyve veren bir ağacın, yalnızca meyveleri tarafından 953.9 g/ağaç N tüketildiğini açıklamışlardır. Bu nedenle bazı araştırmacılar, antepfıstığında görülen meyve ve salkım silkmesi sonucu ortaya çıkan verim düşüklüğü ve periyodisitenin, doğrudan gübrelemeden kaynaklandığını savunmaktadırlar. Bunlara göre, antepfıstığında verim ve kaliteyi artırmak, periyodisiteyi azaltmak için yapılması gereken ilk iş; yeterli ve dengeli bir gübreleme yapmaktır (Aydeniz., 1990). Ancak, uygun gübreleme için, elde uygun bir programın olmaması, verilecek gübrelerin sulama mevsimi dışında bir defada uygulanması, periyodisitenin şiddetinin azaltılmasında beklenen etkiyi sağlayamamıştır (Kanber ve ark., 1990).

Rosecrance ve ark. (1995), verim yılındaki antepfıstığı ağacının, meyve içi dolum döneminde (Mayıs sonu-Eylül başı), periyodisite yılına göre, % 35 daha fazla N alımı olduğunu saptamışlardır. Azot alımının, hasat ve yaprak dökümü arasındaki dönemde dikkate alınmayacak kadar küçük, meyve içi dolum döneminde daha yüksek düzeyde olduğunu bildirmişlerdir. Bununla beraber, verim yılındaki ağaçlarda, yeniden ürün için daha fazla azot alımına ve karbonhidrata gereksinim olduğunu, ağacın uzun ömürlü dokularında karbonhidrat ve azot birikimleri azaldığını ve Ocak ayında yapılan azotlu gübrelemede topraktan azot kaldırılmasının daha düşük düzeyde gerçekleştiğini belirtmişlerdir.

Bir başka çalışmada Rosecrance ve ark. (1997), meyve yılındaki antepfıstığı ağacının toprak üstü organlarının (yaprak, meyve, yıllık sürgün), ağacın toplam azotunun % 60' ını içerdiğini açıklamışlardır. Bunun karşıtı olarak, meyvesiz yılda meyvenin olgunlaşma döneminde, ağacın toplam azot miktarının % 30' undan daha

az kısmının tek yıllık organlarda bulunduğunu belirlemişlerdir. Araştırmacılar, meyveli ve meyvesiz yıllarda antepfıstığı ağacının sırasıyla 167 ve 153 kg/ha/yıl azot soğurduğunu saptamışlardır.

Antepfıstığının fosfor ve potasyum noksanlık belirtileri ve giderilmeleri üzerinde çalışan Aswort ve ark. (1985), potasyum noksanlığında seyrek, küçük yapılı, erken dökülen, sarı renkli yaprakların oluştuğunu; bu yapraklardaki potasyum düzeyinin % 0.8' den az olduğunu, 3 kg/ağaç KCl verildiğinde yaprakların sağlıklı duruma gelip verimin arttığını ve yaprakların K düzeyinin % 1.1 –2.2 arasında bulunduğunu belirtmişlerdir. Aynı araştırmacılar, fosfor noksanlığında yaprakların normal büyüklükte olmalarına karşın, önce ucundan başlayan daha sonra tüm yaprağı kaplayan sararmaların olduğunu ve bunların daha sonra döküldüğünü açıklamışlardır. Her ağaca 1.2 kg % 76' lık P_2O_5 eriyiğinin sulama suyu ile verilmesi durumunda, yapraklardaki fosfor düzeyinin % 0.06 dan % 0.15' e kadar çıkabildiğini gözlemlemişlerdir.

Zeng ve ark. (1999), antepfıstığında potasyumlu gübreleme ve potasyum eksikliğinin tanımlanması ile ilgili ölçütlerin belirlenmesi üzerine yaptıkları çalışmada, potasyum gübrelemesinin, yapraklarda potasyum derişimini, verim ve kaliteyi arttırdığını; potasyum için % 95 maksimum verim düzeyinde kritik değerin % 1.67 ile % 2.02 arasında olması gerektiğini rapor etmişlerdir.

Potasyum gübrelemesi, yapraktaki potasyum düzeyini, verim ve meyve niteliğini önemli ölçüde etkiler. Meyveli yılda antepfıstığı ağacı, meyvesiz yıla göre, yaklaşık iki kat daha fazla K soğurur. Meyveli yılda 218 kg/ha K tüketirken, bu oran meyvesiz yılda 96 kg/ha' a düşer. Rosecrance ve ark. (1995)' na göre; meyvesiz yılda, meyveli yıla oranla antepfıstığı ağacı tarafından % 112 oranında, daha fazla K kaldırılmaktadır. Meyveli ve meyvesiz yılda, K alımının % 90' ı meyve içi dolum döneminde gerçekleşir. Hasat sonrası ve ilkbahar sürgünleri döneminde K alımı düşüktür. K' un önemli bir bölümü, meyvelerle ve yaprakların dökülmesiyle ağaçtan uzaklaşır. Gerçekte, tam gelişmiş olgun bir ağaçta K' un yarıdan fazlası, tek yıllık organlarla ağaçtan kaldırılır (Zeng ve ark., 1997).

Steduto (1984), fertigasyon uygulamalarının yalnızca makro element uygulamaları için değil, aynı zamanda mikro elementler (Zn, Cu, Co, Mn, ve Fe) için

de ayrı bir öneme sahip olduğunu belirtmektedir. Özellikle mikro besin elementleri uygulamasında karşılaşılan bazı sorunların çözümünde fertigasyon uygulamalarının etkili bir yöntem olduğunu, mikro besin elementlerinin şellat formunda uygulanmasıyla değinilen sorunların başarılı bir şekilde çözüldüğünü, özellikle Fe ve Zn elementlerinin, damla sulama suyu ile birlikte Kaliforniya' da limon ve avokado üzerine başarılı bir şekilde uygulandığını yazmıştır. Bu bağlamda mikro besin elementlerinden olan demir, bakır ve çinko, antepfıstığı için ayrı bir öneme sahiptir. Bunlardan demir ve çinko klorofil oluşumu için gerekli iken, bakır bu oluşuma katkıda bulunur. Antepfıstığı yetiştirilen toprakların yüksek düzeylerde kireç içerdiği ve alkali özellik gösterdiği, pH' nın 9.3'e kadar çıktığı düşünüldüğünde, demirin çözünürlüğü aynı oranda azalacağından, bitkiler tarafından alımı güçleşmektedir. Demir ve bakır noksanlığında bitkilerde klorofil oluşumu yetersiz kaldığından, kloroz hastalığı görülür. Kloroz hastalığında, bitkilerin yaprakları açık sarı renk alır. Yaprak damarları koyu yeşil kaldığı halde damarlar arası açık renklidir. Bakır noksanlığına, tarıma yeni açılan ve fazla organik madde içeren topraklarda ve ayrıca kumlu ve çakıllı olan mineral topraklarda rastlanılmaktadır. Bakır noksanlığının olduğu bitkilerde, gelişme geriliği görülür. Topraklarda bakırın fazla olması, demir alımını güçleştirdiğinden bitkilere zehir etkisi yapmaktadır. Çinko noksanlığı, daha çok üst toprak katmanı taşınmış veya organik maddece fakir topraklarda görülür. Çinko noksanlığı, bitkilerin yapraklarında orta damar yeşil kaldığı halde, damarlar arası beyaz renk almasıyla tanınır. Yapraklar, yeşil ve sarı renkte olmayıp, daha çok açık ten renginde veya soluk kahverengi bir renk alır (Ergene, 1982).

Toprakta fazla kirece bağlı olarak, antepfıstığı ağacında demir noksanlığı konusunda çalışan Sepaskhah ve ark. (1985), 2 mg/l DPTA çözeltisinin yaprağa uygulanmasının yeterli ve yapraklarında 83 mg/l Fe bulunan ağaçların verimli olduğunu bildirmişlerdir.

Antepfıstığında noksanlığı kısmen görülen Zn ile ilgili olarak, Uriu ve Pearson (1987) tarafından yapılan bir çalışmada, 2-3 mg/l ve daha az Zn içeren yapraklarda noksanlık belirtisi olarak; geç uyanma, boğum araları ve yaprakların küçük kalması ve kırmızı renkte olmaları gösterilmiştir. Buna karşın, 7 mg/l' in üzerinde Zn içeren yapraklarda sıralanan belirtilerin ortaya çıkmadığı kaydedilmiştir.

Çinko noksanlığı görülen ağaçlarda meyvelerin küçük kaldığı ve verimin düşük olduğu; bu ağaçlara % 36'lık $ZnSO_4$'tan 2-2.5 kg/ağaç veya Zn EDTA şelatından 1-1.5 kg/ağaç verilmesiyle noksanlığın giderildiği ve verimin arttığı saptanmıştır.

Zeng ve Brown (1999a) tarafından, yetişkin antepfıstığı ağacında ve *Pistacia vera* L. tohumlarında ^{68}Zn uygulanarak, Zn' nin dağılımı ve taşınımı incelenmiştir. Araştırma sonunda, yapraklar tarafından soğrulan Zn' nin yaklaşık % 5.4' ünün, uygulama yapılan yapraklardan taşındığı rapor edilmiştir. Taşınan Zn, bitkinin diğer organlarında değişik miktarlarda saptanmıştır. Yetişkin antepfıstığı ağaçlarında Zn taşınımı, akropetal ve basipetal yapraklarda, ama daha çok basipetal yapraklarda meydana gelmektedir. Bununla beraber uygulamadan 10 gün sonra, yapraklarda taşınımın önemli olmadığı anlaşılmıştır. Uygulamadan 20 gün sonra diğer bitki dokularına taşınan Zn miktarı daha fazla bulunmuştur. Uygulama gelişmesini tamamlamış taze yapraklara yapıldığında bu oran, % 6.5 olurken, olgun yapraklarda % 2.1 düzeyinde kalmıştır. Zn' nin antepfıstığı içerisinde hareketinin sınırlı olması, elementin yaprak dokuları tarafından tutulma kapasitesine bağlanmıştır.

Zeng ve Brown (1999b) bir başka çalışmalarında, ceviz ve antepfıstığında Zn' nun alımı ve taşınım düzeyini belirlemek için ^{68}Zn kullanmışlardır. Uygulamadan hemen sonra gelişmesini tamamlamış, olgun ceviz ve antepfıstığı yaprakları, uygulanan toplam Zn' nun yaklaşık yarısı kadarını (% 3.5 ve % 6.5) içlerinde tutmuşlardır. Uygulama yapılan antepfıstığı yapraklarının yaşı, Zn alım kapasitesini etkilemiştir. Tam olgunlaşmamış antepfıstığı yaprakları, olgun ceviz yapraklarından daha fazla Zn soğurmuşlardır.

Picchioni ve ark. (1991), üç antepfıstığı anacının, kireç içeren kumlu-tınlı toprakta ve sera koşullarında bor uygulamasına (0-15 mg/L) gösterdiği tepkiyi araştırmışlardır. Uygulamadan 10.5 ay sonra anaçlarda kök+gövde ağırlığı, yaprak kuru ağırlığı, bitki başına yaprak alanı ve sayısı, bor uygulamasına paralel olarak doğrusal bir azalma göstermiştir. Anaç çeşitleri arasında bor'a karşı duyarlılık bakımından, herhangi farklılık olmadığını belirlemişlerdir.

Antepfıstığı yetiştiriciliğinin yoğun olarak yapıldığı Şanlıurfa yöresinde, bitkinin besin kapsamlarının belirlenmesi ve mikro element eksikliği ile bazı toprak özellikleri arasındaki ilişkiyi araştıran Kızılgöz ve ark. (1999), bitki örneklerinde

yaptıkları analizlerde N, K, Mn, Fe ve Zn' eksikliğinin istatistiksel olarak önemli olduğunu ($P \leq 0.05$) yazmışlardır.

Lovatt (2001), Haziran ve Temmuz aylarında azotlu gübrelerin sprey şeklinde uygulanmasıyla karagöz dökümünün engellendiğini belirtmişlerdir. Benzer olarak; mikro besin elementlerinden Fe ve Zn' -nun yapraklara sprey şeklinde verilebileceğini belirten Tekin (1992), meyve veren ağaçlarda 800 g saf azot, 600 g P_2O_5, 400 g K_2O ve 60 kg çiftlik gübresi ve 3 kez yaprak gübresi uygulamasının meyve kalite özelliklerinden çıtlama oranı, iç meyve randımanı, sürgün uzunluğu, meyve gözlerinin dökülmemesi verim ve verimlilik üzerinde olumlu etki yaptığını, meyvenin protein ve yağ içerikleri üzerinde etkili olmadığını kaydetmiştir. Belirtilen bu miktarlar yaprakların N, P, K, Fe ve Mn içeriklerini de artırmıştır.

Sistemik sıvı gübrelerin antepfistığında verim ve kalite üzerine etkilerini belirlemek amacıyla Bilgen ve ark. (1981) tarafından yapılan bir çalışmada, değişik sıvı gübrelerin, sürgün uzunluklarını ve yaprakların N, P, K, Fe, Mn ve Zn içeriklerini arttırdığı saptanmıştır. Aynı araştırmacılar bu uygulamaların verim ve meyve niteliği üzerine etkilerinden söz etmemelerine karşın, antepfistığında Fe düzeyi ile 100 dane ağırlığı arasında yakın bir ilişkinin olduğu; fakat doğal çıtlama yüzdesi ve randımanı arasında herhangi bir ilişkinin olmadığını belirten Tekin ve ark. (1997), ağaç başına 6 kg demir sülfat uygulamasının doğal çıtlama oranını arttırdığını açıklamışlardır.

3. MATERYAL ve YÖNTEM

3.1. Materyal

3.1.1 Araştırma Yeri

Araştırma, Antepfistığı yetiştiriciliğinin yoğun olarak yapıldığı Güneydoğu Anadolu Bölgesinde bulunan Gaziantep kentinde yapılmıştır (Şekil 3.1). Deneme bahçesi Antepfistığı Araştırma Enstitüsüne bağlı ve merkeze 26 km uzaklıkta, havaalanı güvenlik bölgesi içerisinde bulunan, yaklaşık 3 hektarlık alanda yürütülmüştür. Deneme alanının denizden yüksekliği 705 m olup, 38° 28' doğu enlemi, 36° 57' kuzey boylamındadır.

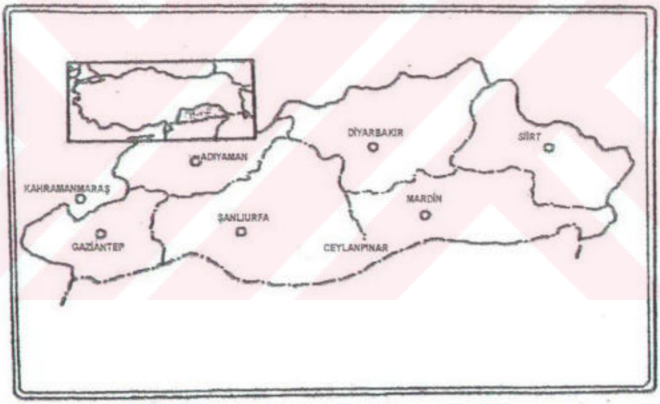

Şekil 3.1. Antepfistığının yetiştiriciliğinin yapıldığı önemli İl'ler ve Gaziantep'in Güneydoğu Anadolu Proje alanındaki yeri

3.1.2 Toprak Özellikleri

Deneme alanı toprakları, Gaziantep-Birecik havzasında ve Karacaveran toprak serisinde yer almaktadır. Kalkerik vertisol topraklardır. Deneme alanı topraklarının sulamaya ilişkin kimi özellikleri, Peters ve Calvin (1965) tarafından

verilen sistematik örnekleme yöntemiyle, bahçeyi temsil eden yerlerden bozulmuş ve bozulmamış toprak örnekleri alınarak belirlenmiştir. Örnekler, 150 cm'lik toprak derinliğinin, 30 cm'lik katmanlarından alınmıştır. Bozulmamış toprak örneklerinde tarla kapasitesi (TK), hacim kütlesi (ρ) değerleri saptanmıştır. Toprak bünyesi, kireç ($CaCO_3$), kil, silt ve kum içerikleri; pH ve solma noktası değerleri ise bozulmuş toprak örnekleri kullanılarak elde edilmiştir. Değinilen analizlerin tümü, Richards (1954) ve Tüzüner (1990) tarafından verilen yöntemler kullanılarak yapılmış ve sonuçlar Çizelge 3.1' de verilmiştir.

Çizelge 3.1. Deneme Alanı Topraklarının Bazı Fiziksel ve Kimyasal Özellikleri

Top.Der. cm	Bünye Sınıfı	TK %	SN %	ρ g/cm^3	pH	Tuz İçeri ği. %	Kireç %	Kil %	Kum %	Silt %
0-30	Kil	37.71	21.13	1.33	7.34	0.116	17.23	73.32	4.13	22.54
30-60	Kil	37.69	21.08	1.15	7.43	0.109	17.24	71.58	2.81	26.27
60-90	Kil	38.05	21.22	1.33	7.56	0.098	18.31	76.21	3.19	20.59
90-120	Kil	37.30	21.26	1.29	7.58	0.095	19.92	77.32	2.93	19.76
120-150	Kil	34.78	21.02	1.39	7.68	0.195	23.75	75.93	4.03	20.27

Deneme alanı topraklarının profil özelliklerini belirlemek amacıyla iki adet profil açılmış, buradan elde edilen sonuçlar Çizelge 3.2 ile Şekil 3.2 - 3.3' de gösterilmiştir.

Çizelge 3.2. Deneme Alanı Topraklarının Profil Özellikleri

Horizon	Derinlik,cm	Tanımlama
Ap	0-10	Kırmızımsı Kahverengi (5YR4/8 kuru) kırmızımsı kahverengi (5YR4/8 ıslak); orta taneli yapıda killi; yüksek oranda kireç içerir; ana kök bölgesinde orta düzeyde taşlı; hafiften orta düzeye doğru taşlı, sınırları dalgalı.
Ad	10-40	Kırmızımsı Kahverengi (5YR4/8 kuru) kırmızımsı kahverengi (5YR4/8 ıslak); killi, sert ve yoğun pulluk tabakası, orta düzeyde ana kökler; sınırları dalgalı
A3ss	40-72	Koyu kırmızımsı kahverengi (5YR3/6 kuru ve ıslak); kuvvetli-orta prizmatik killi; kuru iken sert, ıslak iken kırılgan; yüksek oranda kireç içerir, hafif taşlı; sık ve belirgin parlak kaygan yüzeyli; nadiren ana kökler; sınırları dalgalı
Bk1ss	72-104	Koyu kırmızımsı kahverengi (2.5YR3/4 ıslak); orta sert killi, kuru iken sert, ıslak iken kırılgan; yüksek oranda kireç içerir, hafif taşlı; sık ve belirgin parlak kaygan yüzeyli; nadiren ana kökler; sınırları dalgalı
Bk2ss	104-141	Koyu kırmızımsı kahverengi (2.5YR3/4 ıslak); kuvvetli-orta prizmatik killi; kuru iken sert, ıslak iken kırılgan; yüksek oranda kireç içerir, hafif taşlı,; sık ve belirgin parlak kaygan yüzeyli; nadiren ana kökler; sınırları dalgalı
Ckm	141-170	Donuk turunç (5YR7/4 ıslak); taşlaşmış Kaliş

Çizelge 1' den görüldüğü gibi, deneme alanı toprakları, kırmızı kahverengi toprakların özelliklerini göstermektedir. Profili oluşturan Ap horizonu, 10 cm kalınlıkta olup, granüler yapıda ve orta düzeyde organik madde içermektedir. Yüksek düzeyde kil içeren topraklardır. Yıllık yağışın 250-400 mm olduğu bölgelerde oluşan topraklar olduğundan, B horizonuna doğru kireç ($CaCO_3$) birikimi artmaktadır (Şekil 3.2).

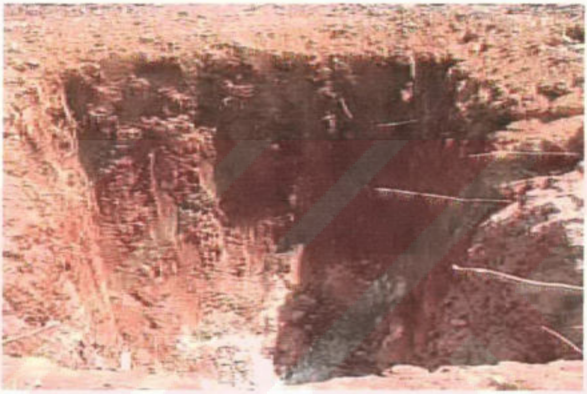

Şekil 3.2. Deneme bahçesinin toprak profili

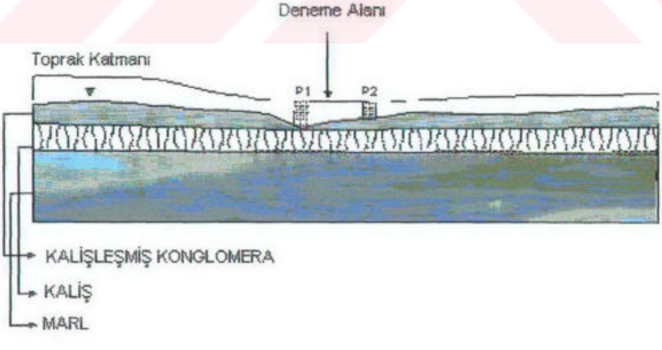

Şekil 3.3. Deneme bahçesi topraklarının jeomorfolojik özelliklerinin görünümü

Şekil 3.3' te deneme alanı topraklarının jeomorfolojik özelliklerinin görünümü verilmiştir. Deneme alanı toprak profili değişik katmanlardan oluşmuştur. Profilin en üst katmanında kalişleşmiş konglomera yer almıştır. Bunun altında kaliş ve marl katmanları bulunmaktadır. Kalişler porozlu, kurak alanlarda bitkinin daha iyi gelişmesini sağlayan gözenekli bir yapıya sahip olup, yüksek oranda su tutabilmektedirler.

3.1.3 İklim Özellikleri

Denemenin yapıldığı Gaziantep yöresi, Akdeniz ile çöl iklimlerinin arasında tipik geçit iklimi özellikleri gösterir.

Denemenin yürütüldüğü yıllara ilişkin kimi iklimsel veriler ve ortalama değerleri Çizelge 3.3' de verilmiştir. Çizelgenin incelenmesinden de anlaşılacağı gibi, denemenin yürütüldüğü 1999 - 2002 yılları ile uzun yıllık iklimsel verilerden, bölgede uzun yıllık ortalama sıcaklık 14.9 °C, en sıcak ay 27.7 °C ile Temmuz, en soğuk ay ise 3.4 °C ile Ocak' tır. Uzun yıllık ortalama yağış 544.3 mm olmasına karşın, yağışların yıl içerisindeki dağılımı türdeş değildir. Yağışların yaklaşık % 90'lık kısmı kış aylarında düşmektedir. Rüzgarlar, baskın olarak güneybatı-kuzeybatı yönünden, en kuvvetli rüzgarlar ise, kuzey-kuzeybatı yönünden esmektedir. Uzun yıllık ortalama oransal nem % 61.5 olarak gerçekleşmiştir. Oransal nem değerleri, sıcaklıkların arttığı yaz aylarında daha düşük, kış aylarında diğer aylara oranla daha yüksek değerler almaktadır.

Gaziantep yöresinde sulama, tarımsal üretim için Şekil 3.4. de görüldüğü gibi yaşamsal önemdedir. Açık su yüzeyi buharlaşması ile aylık yağış değerlerinin yıllık dağılımını gösteren şekilden anlaşılacağı gibi, Mayıs-Ekim ayları arası dönemde aylık yağış miktarları bitki su gereksinimini karşılayamamaktadır. Değinilen dönemde bitkilerin kesinlikle sulanması gerekmektedir. Bu dönemde Gaziantep bölgesinde yetişen bütün bitkiler, sulama suyuna gereksinim duymaktadırlar. Buna göre bitkiler sulanmadıklarında, üründe önemli düşmeler veya hiç verim almama gibi sorunlarla karşılaşılmaktadır.

Deneme yıllarına ilişkin iklim verileri, denemenin yürütüldüğü Havaalanı Meydan Meteoroloji Müdürlüğünden, uzun yıllık ortalama değerler ise Gaziantep Meteoroloji İstasyon Müdürlüğünden alınmıştır.

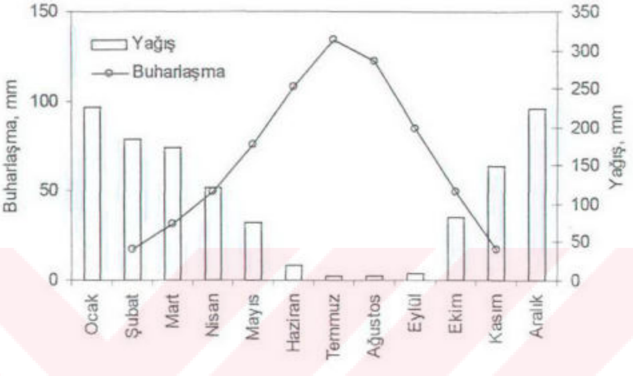

Şekil 3.4. Açık su yüzeyi buharlaşması (Class A Pan) ile yağış ilişkisi

Çizelge 3.3. Denemenin Yürütüldüğü Yıllara İlişkin Kimi İklimsel Veriler ve Uzun Yıllık Ortalama Değerler (1979-2000)

Yıllar	İklim Öğeleri	A Y L A R											
		Ocak	Şubat	Mart	Nisan	Mayıs	Haziran	Temmuz	Ağustos	Eylül	Ekim	Kasım	Aralık
Uzun Yıllık	Maksimum Sıcaklık, °C	13.8	15.4	20.6	27.3	32.4	36.4	39.4	38.9	35.9	30.7	22.4	21.3
	Minimum Sıcaklık, °C	-5.4	-6.1	-2.9	2.1	6.4	15.9	16.3	16.2	15.2	5.1	-0.9	-3.9
	Ortalama Sıcaklık, °C	3.4	4.2	7.8	13.3	18.3	23.9	27.7	27.3	22.9	16.1	9.2	5.0
	Ortalama Oransal Nem, %	75.2	71.7	67.5	64.0	57.6	49.2	46.4	48.8	49.8	61.2	71.8	74.6
	Buharlaşma (CAP), mm	-	-	-	116.3	176.4	251.9	314.1	285.8	197.4	114.7	40.8	0.4
	Yağış, mm (45 yıllık)	96.6	78.6	74.2	52.2	31.9	8.0	1.9	1.6	4.2	35.3	64.1	95.7
	Hava Basıncı, mm	919.8	917.6	916.2	915.7	915.2	912.6	910.3	911.6	915.7	919.2	919.9	920.3
	Ortalama Rüzgar Hızı, m/s	1.9	2.0	2.2	2.2	2.1	2.9	3.1	2.5	1.9	1.4	1.4	1.7
1999	Maksimum Sıcaklık, °C	7.1	11.2	15.4	21.5	28.8	31.8	36.6	36.9	32.2	26.3	18.2	13.7
	Minimum Sıcaklık, °C	-2.0	-4.0	0.0	3.5	10.2	14.2	17.7	16.6	11.0	6.8	-2.4	-1.0
	Ortalama Sıcaklık, °C	5.8	6.2	9.2	14.6	21.1	23.2	28.8	28.3	23.2	18.1	10.5	7.2
	Oransal Nem, %	77.3	76.9	66.6	62.2	57.0	59.3	55.7	55.4	56.8	61.6	62.7	71.2
	Yağış, mm	35.1	77.3	64.9	33.2	5.5	6.6	0.2	0.0	1.0	19.1	3.6	73.5
	Güneşlenme Şid. MJ/m^2 gün	5.5	8.4	11.8	16.5	20.7	20.9	21.9	19.0	15.9	11.6	8.5	6.2
	Buharlaşma (CAP), mm					153.0[a]	305.2	360.0	285.2	15.7[b]			
2000	Maksimum Sıcaklık, °C	17.6	13.2	24.2	27.4	31.2	37.6	44.0	40.4	35.8	31.6	25.2	15.5
	Minimum Sıcaklık, °C	-6.3	-5.0	-5.2	3.0	8.0	14.4	17.0	16.2	10.5	5.2	2.0	2.6
	Ortalama Sıcaklık, °C	2.4	4.0	7.6	15.1	19.3	25.7	31.2	28.2	23.2	15.9	11.2	5.1
	Oransal Nem, %	73.3	66.4	58.2	68.3	58.6	47.7	48.2	59.4	60.6	66.2	61.1	77.3
	Yağış, mm	164.0	77.9	27.1	43.6	7.6	0.0	0.0	0.0	9.0	34.0	48.2	94.8
	Güneşlenme Şid. MJ/m^2 gün	5.7	8.2	12.4	13.1	19.2	22.5	22.5	18.8	16.5	11.3	8.5	4.7
	Buharlaşma (CAP), mm					160.0[c]	324.3	389.6	324.3	237.4	28.7[d]		

[a] 14-31.05.1999 tarihleri arasında ölçülen toplam buharlaşma miktarı 153 mm; [b] 1-2.09.1999 tarihleri arasında ölçülen toplam buharlaşma miktarı 15.7 mm; [c] 9-31.05.2000 tarihleri arasında ölçülen toplam buharlaşma miktarı 160 mm; [d] 1-6.10.2000 tarihleri arasında ölçülen toplam buharlaşma miktarı 28.7 mm' dir.
(CAP; Class A Pan buharlaşma kabı

Çizelge 3.3' ün devamı.

Yıllar	İklim Öğeleri	AYLAR											
		Ocak	Şubat	Mart	Nisan	Mayıs	Haziran	Temmuz	Ağustos	Eylül	Ekim	Kasım	Aralık
2001	Maksimum Sıcaklık, °C	19.0	19.0	26.6	28.8	33.5	39.5	42.6	40.0	36.0	31.6	22.7	11.2
	Minimum Sıcaklık, °C	-2.0	-5.5	3.8	4.4	6.0	14.2	19.0	17.2	12.4	5.5	-7.0	-4.5
	Ortalama Sıcaklık, °C	6.1	5.8	12.7	14.5	17.4	25.5	29.4	28.2	26.5	17.0	8.3	5.2
	Oransal Nem, %	74.2	73.3	67.3	62.7	61.2	36.5	30.0	45.9	61.3	61.3	71.1	85.3
	Yağış, mm	29.4	131.7	81.1	62.7	40.9	0.0	0.0	0.0	1.3	21.0	69.4	202.0
	Güneşlenme Şid. MJ/m^2 gün	5.54	7.95	11.8	15.4	17.7	20.8	19.8	19.1	15.3	11.8	7.6	3.2
	Buharlaşma, (CAP), mm	-	-	-	-	102.6[a]	290.4	346.1	274.8	244.3[b]	-	-	-
2002	Maksimum Sıcaklık, °C	15.6	18.2	24.2	23.7	30.8	36.8	38.7	38.6	35.0	32.5	23.8	13.5
	Minimum Sıcaklık, °C	-4.6	-2.0	0.5	3.4	8.8	13.0	16.0	17.8	12.7	5.3	1.9	1.0
	Ortalama Sıcaklık, °C	2.2	7.4	9.9	11.9	17.6	24.6	28.4	26.5	23.1	18.2	11.8	6.0
	Oransal Nem, %	68.0	62.9	64.9	70.8	55.9	49.6	51.0	53.1	59.7	59.8	71.9	82.0
	Yağış, mm	53.9	50.4	92.5	45.0	33.8	1.4	0.1	25.5	0.4	20.8	25.4	48.7
	Güneşlenme Şid. MJ/m^2 gün	6.6	8.9	11.0	13.2	19.0	20.8	20.1	19.9	15.6	10.8	7.9	
	Buharlaşma (CAP), mm	-	-	-	-	153.9	260.9	301.7	260	153.0	70.4	-	-

[a]19-31.05.2001 tarihleri arasında ölçülen toplam buharlaşma miktarı 102.6 mm; [b] 1-18.09.2001 tarihleri arasında ölçülen toplam buharlaşma miktarı 15.7 mm olarak ölçülmüştür.
(CAP; Class A Pan buharlaşma kabı

3.1.4 Sulama Sistemi

Sulama suyu, deneme alanı içerisinde bulunan ve yaklaşık 250 m derinliğinde iki adet kuyudan sağlanmıştır. Sulama suyu elektriksel iletkenliği (EC) 0.0 - 0.25 dS m^{-1} ve SAR değeri ise 0-10 arasında (C_1S_1 sınıfı) değişmiştir (Çizelge 3.4).

Çizelge 3.4. Deneme Alanındaki İki Kuyunun Sulama Suyu Analizi

Kaynak	EC dS/m	pH	Katyonlar (me/l) Ca	Na	Mg	K	Anyonlar (me/l) CO_3	HCO_3	Cl	SO_4	SAR	Sınıf
Kuyu	0.117	6.5	3.16	2.0	0.4	0.03	0.0	4.22	0.76	0.61	0.25	C_1S_1

Sulama suyu; kontrol birimi (enjeksiyon pompası ekipmanları, filtreler, akış ve basınç ölçerler) ile boru şebekesinden oluşan damla sulama sistemi ile uygulanmıştır (Şekil 3.5). Her ağaç sırası, iki lateral kullanılarak sulanmıştır.

Kuyulardan alınan sulama suyu, iki adet tank'da toplanmış buradan pompa aracılığıyla vortex filtrelerden geçirilerek, fertigasyon sistemindeki farklı azot derişimleriyle karıştırılmıştır. Farklı azot miktarları, sulama suyu ile birlikte ağaçlara uygulanmıştır.

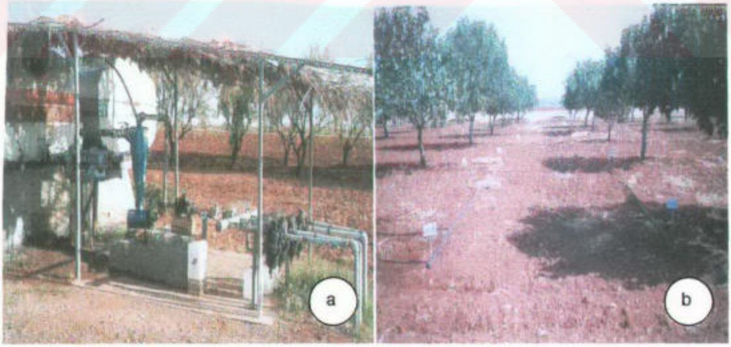

Şekil 3.5. Sulama sistemi denetim birimi (a); yan boru ve lateraller (b).

3.1.5 Toprak İşleme

Deneme alanı toprakları, ilkbaharda ağaçlara su yürüme döneminde, sulama dönemi içerisinde ve hasattan sonra olmak üzere, ilkbahar yağışlarıyla toprağa giren yağmur suyunun korunumu, bahçenin sulamaya hazırlanması ve yabancı ot denetimi için Nisan ayından başlamak üzere 4-5 kez kültüvatörle işlenmiştir. Buna ek olarak, lateraller arasında kalan bölgede çok fazla büyüyen yabancı otlar, hemen her sulamadan sonra çapalanarak yok edilmişlerdir (Şekil 3.6).

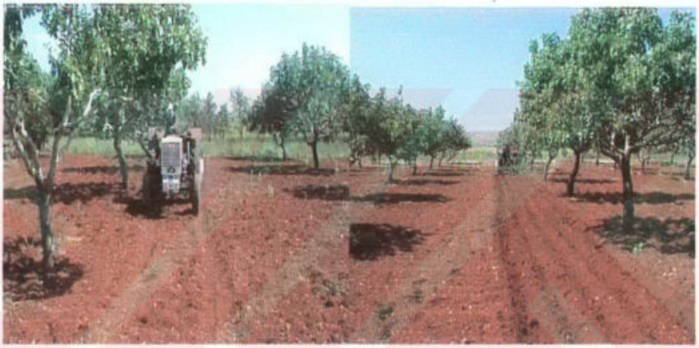

Şekil 3.6. Antepfıstığı bahçesinde toprak işleme

3.1.6 Bitki Çeşidi

Denemede, Gaziantep, Şanlıurfa, Adıyaman ve Kahramanmaraş yörelerinde yaygın biçimde yetiştirilen *Pistacia. vera* L. anacı üzerine aşılı *Uzun* çeşidi kullanılmıştır (Şekil 3.7). Bu çeşidin soğuklama gereksinimi, 600 saat ile Halebi (650 saat) ve Kırmızı (700 saat) çeşitlerinden daha azdır (Küden ve ark. 1992; 1995). Siirt ve Ohadi çeşitlerinden 15-20 gün daha erkenci olması nedeniyle, denizden yüksek yerlerde yetiştirilmesi önerilmektedir (TKB, 1993). Deneme ağaçları, 30 yaşlarında ve 10x10 m aralıkla dikilmiştir.

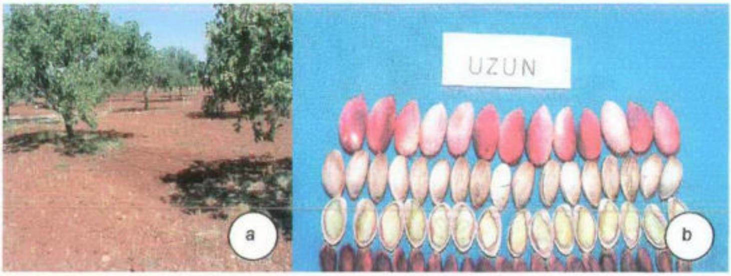

Şekil 3.7. Antepfıstığı ağaçları (a) ve Uzun çeşidinin meyveleri (b)

3.1.7 Kullanılan Aygıtlar

Çalışmada toprak infiltrasyon özelliklerini belirlemek amacıyla çift silindir infiltrometre, toprak su kapsamını belirlemek amacıyla Nötronmetre kullanılmıştır. Sulama suyunun bahçeye uygulanması amacıyla, kuyulardan alınan suyun depolandığı 2 adet 40+15 m^3 hacminde tank, fertigasyon birimi, yaprak alanlarının ölçümünde " optik alan ölçer" kullanılmıştır. Ayrıca, toprak örneklerinde su kapsamını gravimetrik yöntemle belirlemek için gerekli laboratuvar gereçleri kullanılmıştır.

3.2 Yöntem

3.2.1 Deneme Deseni ve Konular

Denemede, farklı sulama ve gübreleme programları ele alınmıştır. İki sulama aralığı (7 ve 14 gün); iki bitki pan katsayısı (K_{cp1}= 0.60 ve K_{cp2}= 0.90); ve dört farklı azot derişimleri (N_g= Geleneksel gübreleme, N_o = 0, N_1= 10 mg/l, N_2=15 mg/l ve N_3= 20 mg/l) kullanılmıştır. N_g olarak simgelenen geleneksel gübreleme konusunda Şubat ayında ağaç başına, saf madde olarak, 500 g N, 600 g P_2O_5 ve 400 g K_2O ağaçların taç izdüşümleri altına açılan bantlara verilmiştir. Fosfor ve potas gübreleri sırasıyla 10 mg/l ve 15 mg/l olarak, 14 gün aralıklarla sulanan konulara eşit miktarlarda uygulanmıştır. Gübreler, sulama uygulamaları başlatıldıktan sonra ve toplam sulama zamanının yarı süresinde fertigasyon sistemine gübre enjektörleri aracılığıyla uygulanmıştır.

Deneme, faktöriyel tesadüf bloklarında, bölünen-bölünmüş parseller (split-split) deneme deseninde, iki yinelemeli olarak düzenlenmiştir. Bunun için yaklaşık 3 hektar (ha) büyüklüğündeki deneme bahçesi, iki bloka ayrılmış ve sulama konuları, şansa bağlı olarak dağıtılmıştır. Her bir konu, 813 m^2 lik alanda 8-10 ağaç içeren bir sıradan oluşmaktadır (Şekil 3.8).

3.2.2 Sulama ve Bitki Su Tüketimi

(i) Sulama

Sulama suyu, deneme bahçesi içerisine yerleştirilen Class A Pan' dan ölçülen açık su yüzeyi buharlaşma değerleri kullanılarak, aşağıdaki eşitlikle hesaplanmıştır (Kanber ve ark. 1999).

$$I = K_{cp} \times E_o \times C \quad (1)$$

Burada; I, sulama suyu miktarı,mm; K_{cp}, bitki-pan katsayısı; E_o, bir haftalık sulama aralığında oluşan toplam buharlaşma, mm; C, ıslatma yüzdesi (denemede % 30 olarak alınmıştır).

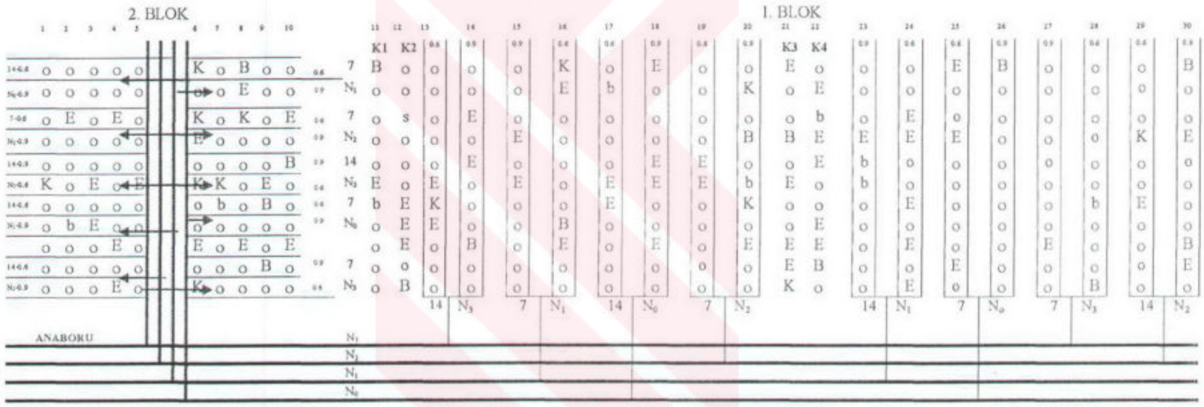

Şekil 3.8 Deneme bahçesinde blokların ve parsellerin şematik gösterimi (K, küçük ağaç; B, boş; b, buttum anacı; s, siirt ağacı; E, erkek; o, meyveli ağacı; $K_{1\text{-}2\text{-}3\text{-}4}$ ise geleneksel konuları temsil etmektedir.)

(ii) Gerçek Bitki Su Tüketimi

Bitkilerin gerçek su tüketimi (ET_c), su bütçesi tekniği kullanılarak aşağıdaki eşitlikle hesaplanmıştır (Kanber ve ark., 1996 dan Cooper ve ark. 1987).

$$ET_c = I + P + C_p - D_p \pm R_f \pm \Delta S \qquad (2)$$

Eşitlikte; ET_c, gerçek bitki su tüketimi; I, sulama suyu miktarı ; P, yağış; C_p, kılcal yükselişle kök bölgesine giren su miktarı; R_f, yüzey akış kaybı; D_p, drenaj veya derine sızma kaybı; ΔS, kök bölgesi toprak su kapsamındaki değişme. Öğelerin tümü mm boyutundadır.

Eşitlikteki öğelerden sulama suyu, yağış ve kök bölgesindeki toprak su kapsamı sulama uygulamalarına ve yağış olaylarına bağlı olarak deneme bahçesinde ölçülmüştür. Yüzey akış ve derine süzülme kayıpları kullanılan sulama yönteminin ve uygulanan kısıntılı sulama tekniği nedeniyle sıfır sayılmıştır (Kanber ve ark., 1996). Deneme bahçesinde, taban suyu olmadığından kılcal yükselişle bitki kök bölgesine giren su miktarının olmadığı kabul edilmiştir. Bu durumda eşitlik;

$$ET_c = I + P \pm \Delta S \qquad (2a)$$

şeklinde kullanılmıştır.

(iii) Kıyas bitki su tüketimi

Kıyas bitki su tüketimi (ET_o), Penman-Monteith eşitliği (Eşitlik 3) kullanılarak kestirilmiştir. Bu amaçla Steduto ve Snyder (1998) tarafından verilen yaklaşımlardan yararlanılmıştır.

$$ET_o = \Delta \frac{\Delta}{\Delta + \gamma}(Rn - G)\frac{1}{\lambda} + \frac{\gamma}{\Delta + \gamma^{*}}(e_s - e)\frac{900}{Tm + 273}(u_2) \qquad (3)$$

Eşitlikte; Δ, günlük ortalama sıcaklıkta buhar basıncı - sıcaklık eğrisinin eğimi (mb/ °C; γ, psikrometrik katsayı; $\gamma = (1+0.34\ u_2)$; γ^*, düzeltilmiş psikrometrik katsayı; R_n, net radyasyon (kJ/ m^2); e_s , günlük ortalama sıcaklıkta doygun buhar basıncı (mb); e, günlük ortalama sıcaklıkta gerçek buhar basıncı (mb); Tm, ortalama sıcaklık (°C); u_2, yerden 2 metre yükseklikteki rüzgar hızı (m/ sn); G, toprak ısı akısı kJ/m^2; λ; buharlaşma gizli ısısı (kJ / kg)

3.2.3 Toprak Su Kapsamının Belirlenmesi

Denemede toprak su içeriği, sulama uygulamalarından bir gün önce ve bir gün sonra ölçülmüştür. Aynı şekilde, toprak su düzeyleri, gelişme dönemi başında (gözlerin patlaması) ve sonunda (yaprak döküm zamanı) belirlenmiştir. Ölçümler, 120 cm'lik profilin, sırasıyla 20 ve 30 cm'lik toprak katmanlarında nötron saçılma ve gravimetrik yöntemlerle yapılmıştır. Bu amaçla örnekler, 120 cm toprak derinliğinde, 30 cm'lik katmalardan, Hollanda tipi "Hand Auger" aleti ile alınarak (Böhm, 1979), kapalı şekilde Antepfıstığı Araştırma Enstitüsü laboratuvarına getirilmiştir. Toprak örneklerinin yaş ağırlıkları belirlendikten sonra, James (1988) ve Tüzüner (1990) tarafından verilen esaslar kullanılarak sabit ağırlığa ulaşıncaya değin etüvde kurutulmuş ve toprak su kapsamları belirlenmiştir. Buna ek olarak, denemeden seçilen iki konuda, $7N_390$ (sulanan) ve "geleneksel" (sulanmayan) , bir ağaca ayrılan alanda evapotranspirasyon yoluyla oluşan su kayıplarının ve su içeriklerinin belirlenmesi için ölçümler yapılmıştır. Bu amaçla, seçilen her bir konu için 10x10 m^2' alanında, 2 x 2 m' lik kareler ağı oluşturulmuştur (Kırda 1999). Izgara sisteminin köşelerine 5 cm çapında 120 cm derinliğinde plastik tüpler yerleştirilmiştir (Şekil 3.9).

Denemede sulama öncesi ve sulama sonrası toprak su kapsamı değişimini izlemek için kare köşelerinde, nötronmetre ölçümleri yapılmıştır (IAEA, 2002). Nötron okumaları, 100 cm derinliğindeki toprağın, 20 cm' lik katmanlarında yapılmıştır. Elde olunan nötron sayımları, Kanber (1997) ve IAEA (2002) tarafından verilen yöntemler kullanılarak, kalibrasyon eğrisinden hacimsel su içeriğine dönüştürülmüştür. Kalibrasyon eğrisi, IAEA (2002)' de verilen esaslardan

C. KIRDA, Prof. Dr., 1999. Sözlü Görüşme. CÜ. Ziraat Fakültesi. Tar. Yap. ve Sul. Böl.

yararlanılarak oluşturulmuştur. Bu amaçla deneme alanını temsil eden ıslak ve kuru koşullardaki, farklı toprak derinliklerinden, hem aynı zamanda nötron okumaları yapılmış, hem de gravimetrik toprak örnekleri alınmıştır (Şekil 3.10).

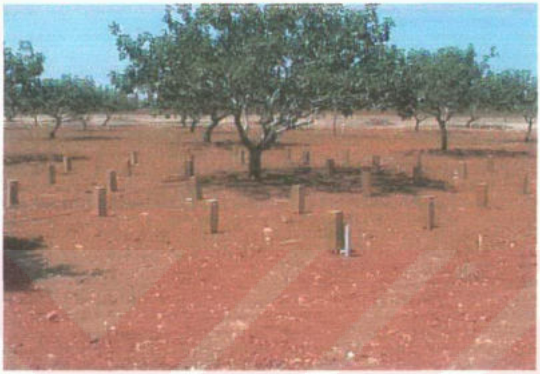

Şekil 3.9. Farklı toprak katmanlarında toprak su içeriğini belirlemek için oluşturulan kareler ağı

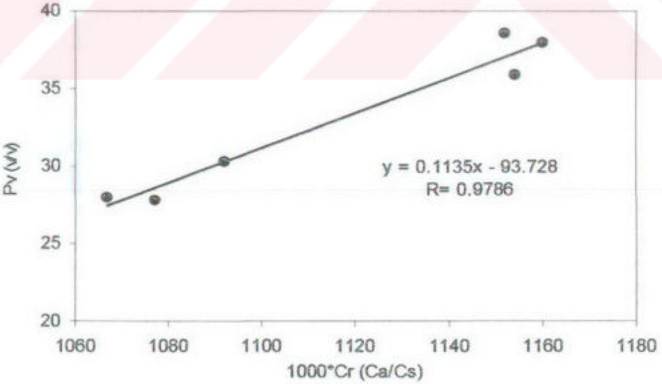

Şekil 3.10. Antepfistığı deneme alanından elde edilen nötronmetre kalibrasyon eğrisi

3.2.4 Deneme Alanı Topraklarının İnfiltrasyon Özellikleri

Deneme alanındaki infiltrasyon testleri, çift silindir infiltrometre ile Criddle ve ark. (1956) tarafından verilen yöntem kullanılarak yapılmıştır. Ölçümlerden elde olunan değerler, Kostiakov eşitliğinde ($Z = a \times T^b$) kullanılarak deneme bahçesi topraklarına ilişkin infiltrasyon katsayıları hesaplanmıştır (Kanber, 1997).

Hesaplamalardan elde olunan sonuçlar, Eşitlik 4, 5 ve Şekil 3.11'de gösterilmiştir.

$$I = 23.2 \times T^{-0.41} \qquad (4)$$

$$Z = 0.38 \times T^{0.60} \qquad (5)$$

Eşitliklerde; I, infiltrasyon hızı (cm/sa), Z, yığışımlı infiltrasyon derinliği, cm; 23.2, 0.41, 0.38 ve 0.60 toprağın hidrolik özelliklerine bağlı infiltrasyon katsayılarıdır.

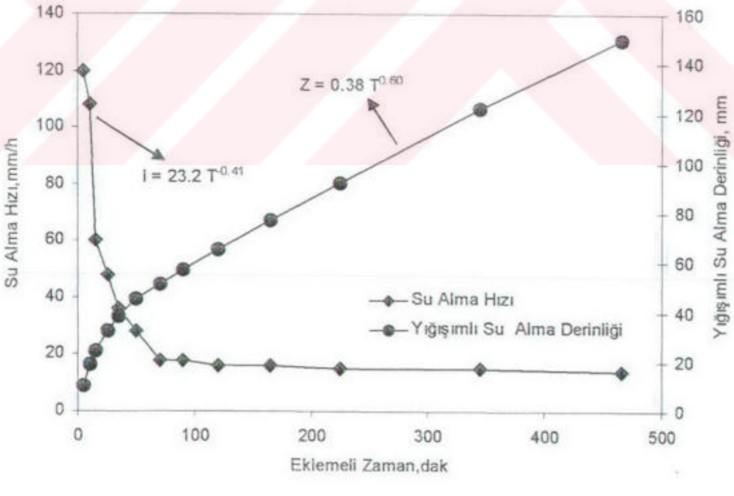

Şekil 3.11. Deneme alanı topraklarının infiltrasyon tavrı

3.2.5 Bitki Büyüme Değişkenlerinin Belirlenmesi

Deneme ağaçlarında kimi bitki büyüme değişkenleri belirlenmiştir. Bu amaçla, deneme konularından şansa bağlı olarak seçilen 3'er ağaç kullanılmıştır. Ağaç yüksekliği, gövde yüksekliği, ağaç taç çapı, taç yüksekliği ve genişliği, ana ve yan dal sayıları, ağaçtaki toplam yaprak sayısı ve birim alana düşen yaprak alanı (LAI), karagöz sayımları, bitki kök yoğunluğu ve yaprak su potansiyeli (YSP) gibi gözlem ve ölçmeler, seçilmiş ağaçlarda yapılmıştır (Şekil 3.12).

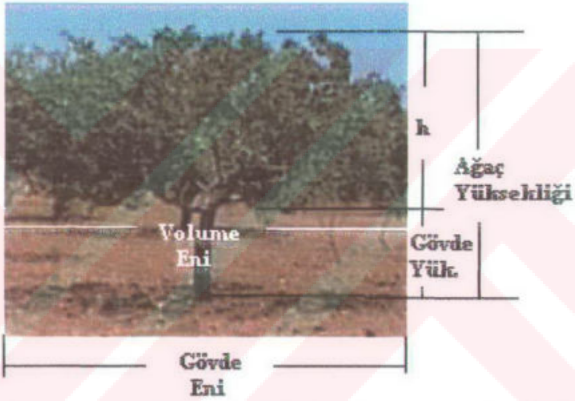

Şekil 3.12. Deneme ağaçlarında kullanılan kimi büyüme değişkenlerinin şematik gösterimi

3.2.5.1 Ağacın fiziksel gelişimine ilişkin ölçmeler

Ağaç yüksekliği ve gövde yüksekliği değerleri, ölçülerek elde edilmiştir. Buradan, ağaç taç yüksekliği; ağaç yüksekliği ile gövde yüksekliği arasındaki farktan hesaplanmıştır. Ağaç taç hacmi, taç yarım küre varsayılarak hesaplanmıştır. Bu hesaplamada taç yüksekliği, kürenin yarıçapı olarak alınmıştır. Ağacın gölge alanı, tacın ortalama çapı kullanılarak elde edilmiştir.

3.2.5.2 Dal sayımları

Denemede işaretlenen ağaçlarda, ana dallar ile bunlar üzerinde seçilen yaklaşık aynı büyüklükteki yan dallar sayılmıştır. Ağaç üzerindeki toplam yaprak sayısını saptayabilmek için, seçilen yan dallar üzerindeki yapraklar sayılmıştır. Toplam yaprak sayısı, yan dallar üzerindeki yaprak sayısı ile ağaçtaki toplam yan dal sayısının çarpımından elde edilmiştir. Birim alandaki yaprak alanı, o ağaçtan alınan yaprak örneklerinin ortalama alanı ile toplam yaprak sayısının çarpılmasıyla bulunmuştur.

3.2.5.3 Yaprak alan indeksi

Antepfistığı ağacının toplam yaprak alanını kestirmek için, kolayca ölçülebilen bazı yaprak değişkenleri ile yaprak alanı arasındaki ilişki incelenmiştir. Bu amaçla, deneme konularında işaretlenen 3' er ağacın dört yöneyinden toplam 20 yaprak örneği alınarak, optik yaprak alan ölçer yardımıyla alanları belirlenmiştir.

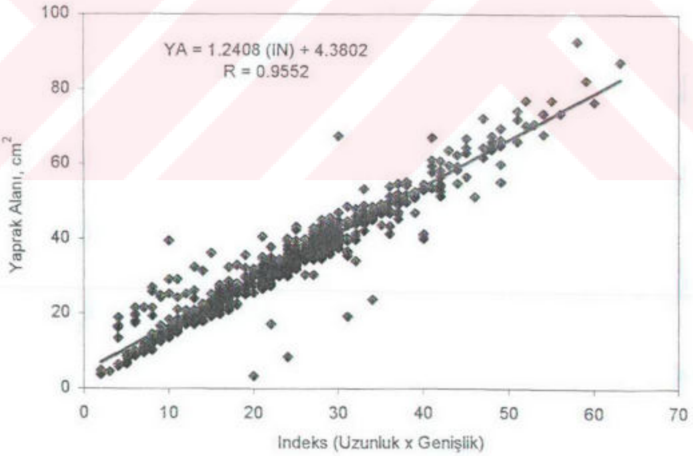

Şekil 3.13. Yaprak alanı ile yaprak indeksi arasındaki ilişki

Aynı örneklerde yaprak uzunluğu ve genişliği ölçülmüş, yaprak alanları ile değinilen ölçütler arasında istatistiksel yöntemlerle ilişkiler araştırılmıştır. Gerçek yaprak alanı değerleri ile yaprak uzunluğu ve yaprak genişliğinin çarpımından elde edilen yaprak indeksi arasında $P \leq 0.01$ düzeyinde önemli ve YA= 1.2408 İN+4.3802 eşitliği ile gösterilen yakın bir ilişki saptanmıştır.

Mevsim içerisinde toplam yaprak sayısının kestirildiği ölçümler sırasında alınan yaprak örneklerinin indeks değerleri saptanmış; anılan değerler Şekil 3.13' de kullanılarak ortalama yaprak alanı belirlenmiştir. Kestirilen yaprak alanı, yaprak sayısı ile çarpılarak bir ağaca düşen toplam yaprak alanı hesaplanmıştır. Yukarıda değinildiği gibi, toplam yaprak alanı kestirildikten sonra, ağaç alanına oranlanarak yaprak alan indeksi (LAI) saptanmıştır (Ünlü, 2000).

3.2.5.4 Meyve gözleri (karagöz) sayımları

Kimi bitki büyüme değişkenlerinin belirlenmesi için seçilen ağaçların her birinde, verimi etkileyen meyve gözleri (karagöz) sayımı yapılmıştır. Bu amaçla ağacın işaretlenen 4 yöneyinden birer yan dal üzerindeki bütün meyve gözleri, Haziran başı - derim sonu olmak üzere, iki dönemde (Tekin, 1992) sayılmıştır. Her iki dönemdeki sayımlar, aynı yan dallar üzerinde yapılmıştır. Birinci ve ikinci sayım sonuçları konulara ilişkin karagöz dökülme oranlarının hesaplanmasında kullanılmıştır.

3.2.5.5 Periyodisite İndeksi

Çalışmada, su ve azot uygulamalarının periyodisite indeksi üzerindeki etkisinin anlaşılması için Wood (1989) tarafından verilen eşitlikten yararlanılmıştır.

$$I = \frac{1}{n-1}\left(\frac{a_2 - a_1}{a_2 + a_1} + \frac{a_3 - a_2}{a_3 + a_2} + ... + \frac{a_{n-1} - a_n}{a_{n-1} + a_n}\right) \qquad (6)$$

Burada, a; yıllara göre değişen verim değeri, n; verim değerlerinin alındığı yıl sayısı. Eşitlikte, deneme boyunca konuların ağaç başına kilogram olarak, kuru kırmızı kabuklu yıllık ortalama verim değerleri kullanılmıştır. Periyodisite indeksi değerinin I = 0 olması, yıllık periyodisitenin olmadığını, I=1 olması ise mutlak periyodisite olduğunu göstermektedir.

Eşitliğin hesaplanmasında Ferguson ve ark. (1995) tarafından önerilen yaklaşım kullanılmıştır.

3.2.5.6 Kök yoğunluğunun belirlenmesi

Ağacın sulama ve gübreleme proğramları ile ilişkili olan kök hacmi, seçilen konularda, çiçeklenme döneminden sonra ölçülmüştür. Bu amaçla su ve azotun farklı miktarlarda kullanıldığı deneme konularında, çok su-sıfır azot ($7N_{0}60$), az su - çok azot ($14N_{3}60$) ve hiç su ve azot uygulaması yapılmayan geleneksel konularından, köşeleri üçken oluşturacak şekilde 3 ağaç seçilmiştir (Şekil 3.14).

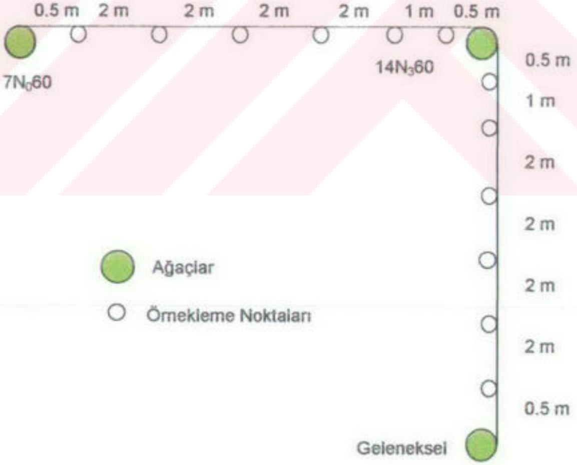

Şekil 3.14. Denemede ele alınan su ve azot değişkenlerine göre oluşturulan kök örnekleme planı

Böylece, çok su verilen ağaçtan hiç sulama yapılmayan ağaca ve hiç azot verilmeyen ağaçtan çok azot verilen ağaca doğru kök gelişimi irdelenmeye çalışılmıştır.

Seçilen konular, birbirine bitişik konulardır. Değinilen ağaçlar arasında, içerisinde kök bulunduran bozulmamış toprak örnekleri, 1090 cm^3 hacime sahip silindirle (6.8 cm çap ve 30 cm yükseklik), 0.5-2 m uzaklıktaki noktalardan ve 120 cm' lik profilin 30cm' lik katmanlarından alınmıştır. Alınan kök örnekler, 0.01 N' lik NaOH çözeltisinde 24 saat bekletildikten sonra yıkanmış ve 30 - 40 ve 50 µm açıklığa sahip farklı elek sistemlerinde ayıklanmıştır. Elde edilen kök örnekleri, 24 saat süreyle, 65 °C de etüvde kurutulduktan sonra ağırlıkları belirlenmiştir (Böhm, 1979).

3.2.5.7 Yaprak su potansiyelinin ölçülmesi

Denemede yaprak su potansiyeli, basınç odacığı tekniği kullanılarak ölçülmüştür (Baştuğ ve Kanber, 1989). Antepfıstığı yaprak su potansiyeli ölçümleri çok su-çok azot ($7N_360$ - $7N_390$), az su- çok azot ($14N_360$ - $14N_390$) ve geleneksel konularında, 3000 Series Plant Water Status Console Pressure Chamber (Soil Moisture Equipment Corp. Coleta CA USA)" aleti[a] ile yapılmıştır (Şekil 3.15).

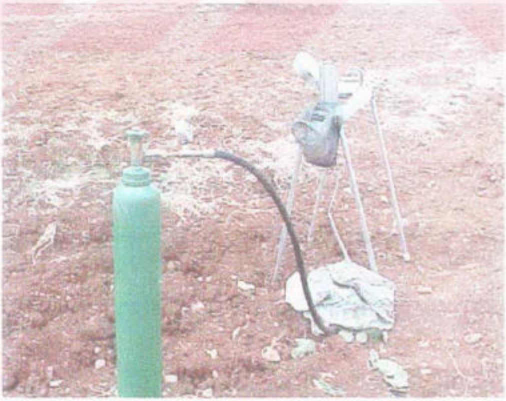

Şekil 3.15. Denemede kullanılan yaprak su potansiyeli (YSP) ölçüm aleti

[a]Reklam amaçlı olmayıp, teknik bilgi olarak verilmiştir.

Ölçümler sulamadan bir gün önce ve bir gün sonra olmak üzere, 2' şer saat aralıkla, gün doğumu ile batımı arasındaki sürede yapılmıştır. Benzer olarak, erken yapılan günlük yaprak su potansiyeli ölçümleri, sulamadan bir gün önce ve bir gün sonra gün doğuşu (şafaktan en fazla sabah saat 8:00 e kadar), öğle vakti güneş tam tepede iken ve gün batımı zamanlarında alınmıştır. Her ölçümde, üzerinde iki veya üç ana dal bulunan, yaprakları tam gelişmiş bir ağaç tacının, dört yöneyinden örnekler alınmıştır. En son yaprak su potansiyeli ölçümü, sulama uygulamaları sona erdikten sonra, derim zamanında yapılmıştır.

3.2.6 Toprak ve Bitki Analizleri

Deneme konularında sulama mevsimi başında , ortasında ve derim zamanı olmak üzere, 3 dönemde, 120 cm' lik toprak profilinin 30 cm' lik katmanlarından toprak örnekleri alınarak, azot kapsamları belirlenmiştir. Böylece sulama mevsimi içerisinde topraktaki azot değişimleri incelenmeye çalışılmıştır. Toprakta, yaprak ve bitki örneklerinde toplam azot, modifiye edilmiş Kjheldahl yöntemine göre belirlenmiş ve sonuçlar % olarak ifade edilmiştir (Kaçar,1972).

3.2.7 Bitki Katsayılarının Belirlenmesi (K_c)

Bitki katsayılarının kestiriminde (K_c) Koumanov ve ark. (1997) tarafından verilen yaklaşım kullanılmıştır. Verilen bu eşitlik, sulama suyu miktarının hesaplanmasında kullanılan ıslatma % sine göre değiştirilmiştir.

$$K_c = \frac{ET_c}{ET_o} x K_r \qquad (7)$$

Burada ; ET_c, antepfistığı su tüketimi (en fazla sulanan konu); K_c, bitki katsayısı; K_r, bitkinin tacı tarafından örtülen toprak alanının, bitki için ayrılan toplam yüzey alanına oranı; ET_o, Penman-Monteith yöntemiyle kestirilen, çim kıyas bitki su

tüketimi. Konu alanının tamamı için, gerçek bitki su tüketimi 0.30 ile çarpılarak hesaplanmış ve K_c hesaplaması için Eşitlik 7' de ET_c' nin yerine koyulmuştur.

Bu hesaplamalar, toprak yüzeyinin herhangi bir örtü bitkisi ile kaplanmamış veya yabancı otların gelişmesine izin verilmeyen koşulların geçerli olduğu varsayılarak yapılmıştır.

3.2.8 Hasat

Antepfistığı ağaçları, denemenin yürütüldüğü dört yıl boyunca, sırasıyla 5, 17, 7 ve 11 Eylül tarihlerinde sulama dönemi bittikten sonra, hasat edilmiştir. Her bir konudaki antepfistığı ağaçları, toplam meyve miktarının belirlenmesi amacıyla elle hasat edilmiştir. Fizyolojik olgunluğa ulaşmış, kırmızı kabuklu (beng) meyveler, ağaçlar silkelenerek; geriye kalan meyveler ise elle toplanmış ve salkımlarından ayrılmışlardır. Verim ve kalite özelliklerinin belirlenmesi amacıyla her bir konudan ağaç başına 1-2 kg meyve örnekleri alınmıştır. Konu edilen örneklerin bir kısmı, meyvenin su içeriğini belirlemek için kullanılmıştır. Bu amaçla örnekler, etüvde 65 °C de sabit ağırlığa ulaşıncaya kadar kurutulmuşlardır.

3.2.9 Su-Verim İlişkileri

Denemede farklı su ve azot düzeylerinin, antepfistığında kuru kırmızı kabuklu meyve verimi üzerine etkilerini saptamak amacıyla varyans analizleri, su kullanma randımanları ve verim fonksiyonlarından yararlanılmıştır.

3.2.9.1 Verim Sonuçları

Deneme konularından su ve azotun farklı düzeyleri ile sulanmayan geleneksel konudan elde edilen ortalama verim miktarları Ek Çizelge 17' de verilmiştir. Konu verimlerinin istatistiksel olarak değerlendirilmesinde Yurtsever (1984)' den

yararlanılmıştır. Ayrıca, verimler ile sulama suyu, su tüketimi ve uygulanan azot miktarları arasındaki ilişkiler irdelenmiştir.

3.2.9.2 Su kullanma randımanları

Denemede sulama ve gübreleme programlarının karşılaştırılmasında, su kullanma randımanları (WUE) dikkate alınmış; anılan değişkenin hesaplanmasında Kanber ve ark. (1996b) tarafından önerilen eşitliklerden yararlanılmıştır. Bu amaçla toplam su kullanma randımanı Eşitlik 8, sulama suyu kullanma randımanı ise eşitlik 9 aracılığıyla hesaplanmıştır (Kanber ve ark.,1993).

$$TWUE = 100\ (E_y / ET) \tag{8}$$

E_y, ekonomik verim, kg/ağaç; ET, bitki su tüketimi, m^3

$$IWUE = 100\ (E_y / IR) \tag{9}$$

IR, sulama suyu, m^3

Sulama suyunun ET içerisindeki kullanım oranı (IR_c) ise, aşağıdaki Eşitlik 10 ile belirlenmiştir.

$$IR_c = 100\ (IR / ET) \tag{10}$$

3.2.9.3 Verim Fonksiyonları

Antepfistığında meyve verimi ile su tüketimi miktarları arasındaki ilişkiler araştırılmıştır. Bu amaçla su-üretim fonksiyonlarının eldesine gidilmiştir. Oransal su tüketimi azalışları ile oransal verim düşüşleri arasındaki ilişkiler belirlenmeye

çalışılmış, bunun için Doorenbos ve Kassam (1979) tarafından verilen eşitlik kullanılmıştır.

$$(1 - Y / Y_m) = K_y (1 - ET / ET_m) \qquad (11)$$

Eşitlikte; Y ve Y_m, gerçek ve maksimum verimleri; ET ve ET_m, gerçek ve maksimum su tüketimleri; K_y ise verim tepki etmenini göstermektedir. Eşitlik 11' in eldesinde, Köksal ve ark. (2001) tarafından önerilen yaklaşımlardan yararlanılmıştır.

3.2.10 Kalite Özellikleri

Hasat sonrası, her bir konudan alınan meyve örnekleri oda sıcaklığında % 2-3 su düzeylerine düşünceye dek kurutulmuştur. Elde edilen kuru meyveler kullanılarak, fis (boş meyve),100 dane ağırlığı, çıtlama oranı, yaş ve kuru meyve ağırlığı ile iç meyve randımanı gibi kimi kalite özellikleri saptanmıştır. Konulara ilişkin değerler üzerinde Yurtsever (1984)' e göre, istatistiksel analizler yapılmıştır.

4. ARAŞTIRMA BULGULARI VE TARTIŞMA

4.1 Sulama ve Su Tüketimi

4.1.1 Sulama Durumu

Denemeye ilişkin sulama tarihleri, sulama suyu ve açık su yüzeyi buharlaşma miktarları Çizelge 4.1-4' de verilmiştir.

Çizelge 4.1. Konulara Uygulanan Sulama Suyu Miktarları (1999)

Sulama Tarihleri	Dönemsel Buharlaşma Miktarı[a] (CAP) mm	Uygulanan Sulama Suyu Miktarı (mm)			
		If_1=7 Gün		If_2=14 Gün	
		Kcp_1=0.6	Kcp_2=0.9	Kcp_1=0.6	Kcp_2=0.9
13.05.99 (ilk sulama)	19.6	3.5	5.3	3.5	5.3
20.05.99	46.0	8.3	12.4		
27.05.99	70.0	12.6	18.9	12.6	18.9
03.06.99	36.5	6.6	9.9		
10.06.99	74.6	13.4	20.1	13.4	20.1
17.06.99	54.6	9.8	14.7		
24.06.99	83.5	15.0	22.5	15.0	22.5
01.07.99	74.7	13.4	20.2		
08.07.99	83.4	15.0	22.5	15.0	22.5
15.07.99	81.8	14.7	22.1		
22.07.99	80.9	14.6	21.8	14.6	21.8
29.07.99	82.6	14.9	22.3		
05.08.99	74.8	13.5	20.2	13.5	20.2
12.08.99	63.5	11.4	17.1		
19.08.99	62.6	11.3	16.9	11.3	16.9
26.08.99	62.6	11.3	16.9		
02.09.99	58.3	10.5	15.7	10.5	15.7
TOPLAM	1110.0	199.8	299.7	109.4	164.1

[a] Ardışık iki sulama aralığında Class A Pan' dan meydana gelen buharlaşma miktarı

Sulama uygulamaları her yıl farklı tarihlerde başlamıştır. Denemenin yürütüldüğü yıllarda sulama mevsimleri iklim koşulları nedeniyle farklı dönemleri kapsamıştır. Çizelgelerden görüleceği gibi, sulama mevsimi Mayıs ayı ortası ile Haziran ayı ilk haftası arasındaki tarihlerde başlatılmış, Eylül ayının ilk yarısında sona erdirilmiştir. Sulama mevsimlerinde oluşan açık su yüzeyi buharlaşma miktarları da, 924-1294 mm arasında değişmiştir. Konulara uygulanan sulama

sayıları da deneme yıllarında farklılıklar göstermiş, sulama aralıklarına göre 18-15 (7 gün) ve 8-10 (14 gün) arasında değişmiştir. Konulara uygulanan toplam sulama suyu miktarları, bitki-pan katsayılarına, buharlaşma miktarlarına ve sulama aralıklarına bağlı olarak değişmiştir. En fazla sulama suyu, 7-90 konusuna verilmiştir. Anılan konuya 2000 yılında 349.4 mm ile en yüksek sulama suyu uygulanmıştır

Çizelge 4.2. Konulara Uygulanan Sulama Suyu Miktarları (2000)

Sulama Tarihleri	Dönemsel Buharlaşma Miktarı[a] (CAP) mm	Uygulanan Sulama Suyu Miktarı (mm)			
		If_1=7 Gün		If_2=14 Gün	
		Kcp_1=0.6	Kcp_2=0.9	Kcp_1=0.6	Kcp_2=0.9
08.05.2000	37.6	6.8	10.2	6.8	10.2
15.05.2000	44.3	8.0	12.0		
22.05.2000	46.1	8.3	12.4	8.3	12.4
12.06.2000	89.0	16.0	24.0	16.0	24.0
19.06.2000	87.0	15.7	23.5		
26.06.2000	72.2	13.0	19.5	13.0	19.5
03.07.2000	76.5	13.8	20.7		
10.07.2000	100.0	18.0	27.0	18.0	27.0
17.07.2000	95.7	17.2	25.8		
24.07.2000	77.4	13.9	20.9	13.9	20.9
31.07.2000	82.6	14.9	22.3		
07.08.2000	82.6	14.9	22.3	14.9	22.3
14.08.2000	74.8	13.5	20.2		
21.08.2000	74.8	13.5	20.2	13.5	20.2
28.08.2000	62.6	11.3	16.9		
04.09.2000	64.3	11.6	17.4	11.6	17.4
11.09.2000	61.7	11.1	16.7		
18.09.2000	64.8	11.7	17.5	11.7	17.5
TOPLAM	1294.0	232.9	349.4	127.6	191.4

[a] Ardışık iki sulama aralığında Class A Pan' dan meydana gelen buharlaşma miktarı

Deneme yıllarında en yüksek su artırımı, 7-90 konusuna göre, 14-60 konusunda 1999 ve 2000 yıllarında % 63.5, 2001 ve 2002 yıllarında ise % 64.2 ve % 62.9 ile elde edilmiştir. En düşük su artırımı ise, 7-90 konusuna göre, 7-60 konusunda 1999 ve 2000 yıllarında % 33.3; 2001 ve 2002 yıllarında ise, % 31.9 ile % 31.0 olarak hesaplanmıştır.

Damla lateralleri ile ıslatılan alan, sulamalardan sonra sulamanın etkinliğini belirlemek amacıyla ölçülmüştür. Sulamalardan sonra toprak yüzeyinde ıslatılan alanın % 30' dan fazla olduğu belirlenmiştir.

Çizelge 4.3. Konulara Uygulanan Sulama Suyu Miktarları (2001)

Sulama Tarihleri	Dönemsel Buharlaşma Miktarı[a] (CAP) mm	Uygulanan Sulama Suyu Miktarı (mm)			
		If_1=7 Gün		If_2=14 Gün	
		Kcp_1=0.6	Kcp_2=0.9	Kcp_1=0.6	Kcp_2=0.9
29.05.2001	39.0	11.7	11.7	11.7	11.7
05.06.2001	65.2	11.7	17.6		
12.06.2001	61.7	11.1	16.7	11.1	16.7
19.06.2001	65.2	11.7	17.6		
26.06.2001	74.8	13.5	20.2	13.5	20.2
03.07.2001	73.0	13.1	19.7		
10.07.2001	74.8	13.5	20.2	13.5	20.2
17.07.2001	80.0	14.4	21.6		
24.07.2001	83.5	15.0	22.5	15.0	22.5
31.07.2001	76.5	13.8	20.7		
07.08.2001	63.5	11.4	17.1	11.4	17.1
14.08.2001	66.1	11.9	17.8		
21.08.2001	57.4	10.3	15.5	10.3	15.5
28.08.2001	62.6	11.3	16.9		
04.09.2001	60.0	10.8	16.2	10.8	16.2
TOPLAM	1003.3	185.3	272.1	97.3	140.1

[a] Ardışık iki sulama aralığında Class A Pan' dan meydana gelen buharlaşma miktarı

Çizelge 4.4. Konulara Uygulanan Sulama Suyu Miktarları (2002)

Sulama Tarihleri	Dönemsel Buharlaşma Miktarı[a] (CAP) mm	Uygulanan Sulama Suyu Miktarı (mm)			
		If_1=7 Gün		If_2=14 Gün	
		Kcp_1=0.6	Kcp_2=0.9	Kcp_1=0.6	Kcp_2=0.9
04.06.2002	58.2	17.5	17.5	17.5	17.5
11.06.2002	53.9	9.7	14.6		
18.06.2002	60.9	11.0	16.4	11.0	16.4
25.06.2002	69.6	12.5	18.8		
02.07.2002	69.6	12.5	18.8	12.5	18.8
09.07.2002	73.0	13.1	19.7		
16.07.2002	57.4	10.3	15.5	10.3	15.5
23.07.2002	65.2	11.7	17.6		
30.07.2002	73.0	13.1	19.7	13.1	19.7
06.08.2002	69.6	12.5	18.8		
13.08.2002	60.9	11.0	16.4	11.0	16.4
20.08.2002	60.9	11.0	16.4		
27.08.2002	52.2	9.4	14.1	9.4	14.1
03.09.2002	52.2	9.4	14.1		
10.09.2002	47.0	8.5	12.7	8.5	12.7
TOPLAM	923.6	173.2	251.1	93.2	131.1

[a] Ardışık iki sulama aralığında Class A Pan' dan meydana gelen buharlaşma miktarı

4.1.2 Bitki Su Tüketimi

Denemede en yüksek azot dozunun uygulandığı konulardan, sulama suyunun çok ve az verildiği uygulamalarla geleneksel konuya ilişkin mevsimlik su tüketimleri, uygulanan sulama suyu, bitki kök bölgesindeki toprak suyu değişimi ve yağış miktarları göz önüne alınarak hesaplanmış, elde edilen sonuçlar Çizelge 4.5' de gösterilmiştir. Hesaplamada gelişme mevsimi boyunca düşen yağışın tümü, miktar ve sıklık nedeniyle etkili kabul edilmiştir (Kanber ve ark. 1986' dan Beyce ve ark. 1972).

Deneme konularının bitki su tüketimleri, yıllara ve konulara göre farklılıklar göstermiştir. Konulardan elde edilen en yüksek ve en düşük bitki su tüketimi, 7 günlük sulama aralığında 0.90 katsayısının kullanıldığı konu ($7N_390$) ile 14 günlük sulama aralığında 0.60 bitki pan katsayısının kullanıldığı ($14N_360$) konularda ölçülmüştür. Buna göre, 1999 yılında 1076 mm ile 467.6 mm arasında, 2000 yılında 1405-615 mm, 2001 yılında 1152-572 mm ve 2002 yılında ise 1228-701 mm arasında değişme göstermiştir. Herhangi bir sulama uygulamasının yapılmadığı "Geleneksel" konusunda bitki su tüketimi değerleri, deneme yıllarında sırasıyla 91.5 mm, 198 mm, 239 mm ve 376 mm olarak hesaplanmıştır. Konular arasında bitki su tüketimi yönünden önemli farklılıklar vardır. Sık sulanan ve çok su alan $7N_390$ konusuna göre, $7N_360$ konusu ile $14N_360$ konularında meydana gelen bitki su tüketimi azalışı, 1999 yılında % 29.9 ile % 56.6 arasında değişirken, bu oranlar diğer deneme yıllarında (2000-2001-2002) sırasıyla % 34.0 – 56.2, % 27.8-50.3 ve % 26.6-42.9 olarak bulunmuştur (Çizelge 4.5).

Sulama yapılan konularda ortalama mevsimlik bitki su tüketimi, deneme yılları ortalaması olarak 414.6 mm olarak hesaplanmıştır. Kanber ve ark. (1993), antepfistığı mevsimlik su tüketimini 803 mm, Bilgel ve ark. (1999), 600 mm, Goldhamer ve ark. (1985) ise 1018 mm olarak bulmuşlardır. Bulgular arasındaki bu farklılıklar, deneme yerinin iklim ve toprak özellikleri ile kullanılan sulama yönteminden kaynaklandığı düşünülebilir. Şener (1993)'ün bildirdiğine göre Cooper ve ark. (1987), yağışın az, toprak kalınlığının fazla olduğu kurak ve yarı kurak bölgelerde sulama yapılmaksızın bitki su tüketiminin, hemen hemen yağışa eşit

Çizelge 4.5 Bazı Konuların Mevsimlik Su Tüketimi

Konular	Toprak Suyu (mm)				Yağış (mm)				Sulama Suyu (mm)				ET (mm)			
	1999	2000	2001	2002	1999	2000	2001	2002	1999	2000	2001	2002	1999	2000	2001	2002
$7N_360$	77.9	29.0	130.0	87.0	10.7	122	85	237	666	776.0	617.0	577.0	754.3	927.0	832.0	901.0
$7N_390$	67.0	119.0	161.0	154.0	10.7	122	85	237	999	1164.0	906.0	837.0	1076.7	1405.0	1152.0	1228.0
$14N_360$	92.9	68.3	163.0	153.0	10.7	122	85	237	364	425.0	324.0	311.0	467.6	615.3	572.0	701.0
$14N_390$	85.0	87.0	164.0	160.0	10.7	122	85	237	547	638.0	467.0	437.0	642.7	847.0	716.0	834.0
Geleneksel	80.8	75.7	154.2	139.0	10.7	122	85	237	0	0.0	0.0	0.0	91.5	197.7	239.2	376.0

olduğunu belirtmişlerdir. Sunulan çalışmadaki geleneksel konuya ilişkin bitki su tüketimlerindeki görülen farklılıklar, yağış rejimindeki dalgalanmadan kaynaklanmaktadır. Kanber (1984)' ün bildirdiğine göre, Jensen ve Tekinel (1973), açık su yüzeyi buharlaşmasının, yüksek sıcaklığa, rüzgar hızına ve atmosferin buharlaşma açığına bağlı olarak arttığını belirtmişlerdir. Deneme yılları arasında, bitki su tüketimlerinde meydana gelen farklılıklar, sulama dönemi içerisinde değinilen iklimsel etmenlerin yüksek olduğu dönemlerde buharlaşma miktarını da artırmıştır. Deneme konularına sulama dönemi içerisinde uygulanan sulama suyu miktarları ve dolayısıyla bitki su tüketiminde meydana gelen farklılıkların, açık su yüzeyi buharlaşmasından kaynaklandığı söylenebilir.

Yığışımlı su tüketimi eğrilerinden yararlanılarak, aylık ET değerleri elde edilmiştir. Aylık ortalama antepfıstığı su kullanımında meydana gelen değişmeler (ET_c), denemenin yürütüldüğü 4 yıl boyunca, aynı azot düzeyinde (N_3), sık sulama ile fazla su uygulanan ($7N_390$) konusu ile Penman-Monteith yaklaşımı kullanılarak hesaplanan çim kıyas bitki su tüketimi (ET_o) konuları için hesaplamalar yapılmış ve Şekil 4.1 de gösterilmiştir.

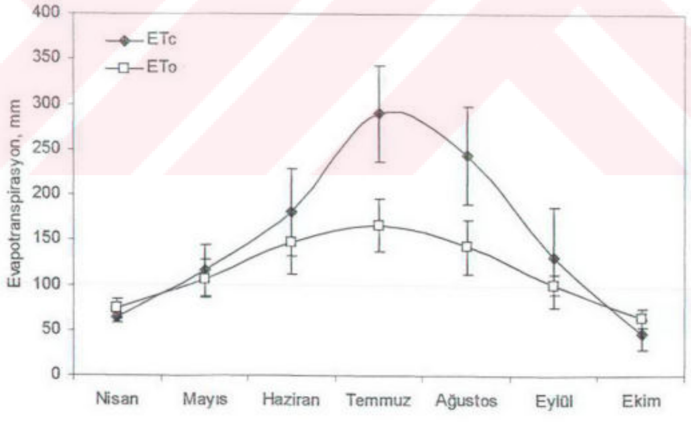

Şekil 4.1. Sık sulanan ($7N_390$) konusuna ilişkin antepfıstığı su tüketimi ile çim kıyas su tüketimi değerlerinin zamansal değişimleri (veriler, 4 yıllık ortalama değerleri, düşey hata çubukları ise ± standart sapmayı göstermektedir.)

Ortalama en yüksek aylık bitki su tüketimi, 7 günlük sulama aralığında 0.90 bitki pan katsayısının kullanıldığı konuda, 290.0 mm ile Temmuz ayında elde edilmiştir. Bu ayda, çim kıyas bitki su tüketimi (ET_o) ise 166.4 mm olarak hesaplanmıştır. Bu değer, diğer aylık değerlere oranla daha yüksektir. Büyüme dönemi başında ve hasat döneminde bitki su tüketimi, sulama sezonundakinden daha düşük bulunmuştur. $I_7$90 konusu için ölçülen ET_c' de oluşan değişmeye benzer olarak, ET_o' da benzer değişme bulunmuştur. Sulama mevsimi boyunca bitki su tüketimi ile çim kıyas bitki su tüketimi (ET_o-PM) arasındaki paralellik aynen devam etmiştir (Şekil 4.1). Kanber ve ark. (1993), yaptıkları çalışmada, antepfıstığında en yüksek aylık bitki su tüketimini, Ağustos ayında 205 olarak bulmuşlardır. Sunulan araştırma bulguları ile aradaki farklılık, USDA-SCS. (1967), Jensen ve ark. (1990) ve Kanber (1997) tarafından da belirtildiği gibi, araştırma alanlarının konum farklılıklarından, sulama programlarından kaynaklanmış olabilir.

4.1.3 Bitki Su Tüketiminin Yersel Değişimi

Sık sulanan $7N_3$90 konusu ile "Geleneksel" konularında, bir ağaç için kabul edilen etki alanından (12x12 =144 m^2) evapotranspirasyon yoluyla oluşan su kayıpları saptanmıştır. Anılan kayıpların yatay boyuttaki değişimleri Şekil 4.2-7' de; düşey boyuttaki yüzde değerleri Şekil 4.8' de gösterilmiştir. Su kayıpları, yıl içerisinde yapılan yalnızca 5 sulama için hesaplanmıştır. Diğer taraftan, sulanmayan konular için bitki su tüketimi (ET) değerleri, bütün bir sulama mevsimi boyunca toplam olarak alınmıştır.

Anılan şekillerde görüldüğü gibi, sulanan konularda oluşan bitki su tüketimi (ET), sulanmayan konulara göre, daha yüksek bulunmuştur. Genel olarak, sulanan konulara ilişkin bütün şekiller, her iki laterale yakın alanlarda bitki su tüketiminin daha yüksek, fakat, ağacın yakınında ve D, E, F hatlarının sonlarına doğru çok düşük olduğunu göstermektedir. Buradan, bitki su tüketiminin yüksek olduğu noktalarda kök yoğunluğunun fazla olduğu, buna karşın, C ile D noktalarında ise, kök yoğunluğunun çok düşük olduğu söylenebilir.

Sulama yapılan konularda, damla sulama sisteminde, bitki su tüketimi yoluyla 100 cm' lik toprak derinliğinden oluşan su kaybı, A hattı boyunca oldukça yüksek bulunmuş, ağaca doğru yaklaştıkça su kaybı miktarı azalmıştır. Damla sulama sistemi yerleştirilmiş olmasına karşın, ağaç gövdesi altında ölçülen bitki su tüketimi, daha düşüktür. Doğrudan damla sulama sistemi ile uygulanan en yüksek bitki su kullanımı, sulanan ağaçtan uzak noktalarda ölçülmüştür. İlk 20 cm' lik toprak katmanında meydana gelen toplam su kaybı, diğer kök katmanlarından meydana gelen su kaybından daha yüksektir. Bu katmanda, ağaca ayrılan alandaki diğer noktalara kıyasla, sulama sistemi altına yerleştirilen noktalarda en yüksek bitki su tüketimi hesaplanmıştır.

Diğer toprak katmanlarında farklı su kayıp desenleri vardır. Bunlardan 20-40 cm ve 40-60 cm' lik katmanlarda, A ve B hatlarının sulama sisteminin dışında kalan, ve ağaca uzak olan alanlarında su kaybı daha yüksektir. Geriye kalan diğer katmanlarda, sulama sistemi altındaki ağaçtan uzak yerlerde daha yüksek su kullanımı ölçülmüştür.

Sulanmayan geleneksel konunun toplam profilde ve ayrı ayrı bütün katmanlarında bitki su tüketimi, ızgara köşelerinde hatların başlangıç ve bitiş yerlerinde, genel olarak çok yüksektir. Bu durum, 0-20 cm'lik katmanda en düşük, 20-40 cm'lik katmanda en yüksektir. Ağaca yakın yerlerdeki bütün katmanlarda bitki su tüketimi, çok düşüktür. En düşük bitki su tüketimi değeri, ağaca yakın yerlerdeki 0-20 cm'lik katmanda sıfır olarak ölçülmüştür. "Geleneksel" konusunda, F hattında sulanan konuların aksine bitki su tüketimi, en düşük düzeydedir. Hatların başlarında ve sonlarındaki değinilen kare köşeleri, sulanan her iki komşu konudan etkilenmiş olabilir. Böylece, sulanan konularda da gözlendiği gibi, bitki su tüketiminin, kök yoğunluğunun çok yüksek olduğu ağaç tacı dışındaki bölgelerde meydana geldiği söylenebilir.

Bitki su tüketimi yoluyla oluşan su kayıplarında, en yüksek bitki su kullanımı, ağaca ayrılan alanın köşe noktalarında ölçülmüştür. Genel olarak bitki su tüketimi, ağaç tacına doğru düşmüştür. Sulanan ve "Geleneksel" konunun her ikisinden elde edilen sonuçlar, antepfıstığı köklerinin ağaç tacı altında etkin

olmadığını göstermiştir. Ele alınan konulara ilişkin toplam ET miktarlarının her bir toprak katmanından kaldırılan % değerleri birbirinden farklıdır (Şekil 4.8).

Olsson ve Rose (1988) antepfistığında, kök etkinliğinin, sulama programları ve yağıştan etkilendiğini saptamışlardır. Temel olarak, bitki su tüketiminin yüksek olduğu toprak katmanlarında, kök etkinliği yüksek bulunmuştur. Sunulan çalışmada sulanan konularda ağaç kökleri toprak yüzeyine yakın yerlerde "Geleneksel" konudan daha etkindir. Farklı katmanlardan kaldırılan toprak suyunun dağılım deseni, sulanan konularda % 27.2 den % 13.7' ye kadar değişmiştir. 0-20 cm'lik ilk katmanda en yüksek su kaybı % 27.2 olarak ölçülürken,"Geleneksel " konusunda aynı toprak katmanında bu oran, % 16.2 olarak ölçülmüştür. Bu konuda antepfistığı kullandığı suyu, kök bölgesinin altında daha derinden almıştır. Toprak profilinin ilk 20-60 cm'lik katmanında bitki su tüketimi yoluyla oluşan toprak su kaybı, % 63 olarak bulunmuştur. Tüketimin büyük bölümünün bu katmanda meydana gelmesi, toprak profilinin aynı derinliklerinde bulunan kaliş katmanından ileri gelmiş olabilir. Bilindiği gibi, sözkonusu katman yüksek su tutma kapasitesine sahiptir (Akça ve ark, 2003)

Lewin ve ark. (1972) ile Kanber ve ark. (1992) tarafından gösterildiği gibi, sulama ile toprağın üst katmanlarından oluşan su kayıpları artmıştır. Diğer taraftan, Goldhamer ve ark. (1989), damla sulama konularında en yüksek toprak su düzeyi, 60 cm'de elde edilmiş, gömülü damla sisteminde 40 cm'nin üzerinde köklerin görülmediğini belirtmişlerdir. Bu sonuçlar göz önüne alınarak, "Geleneksel" konuda görüldüğü gibi sulama, doğal su dağılım desenini etkilemektedir.

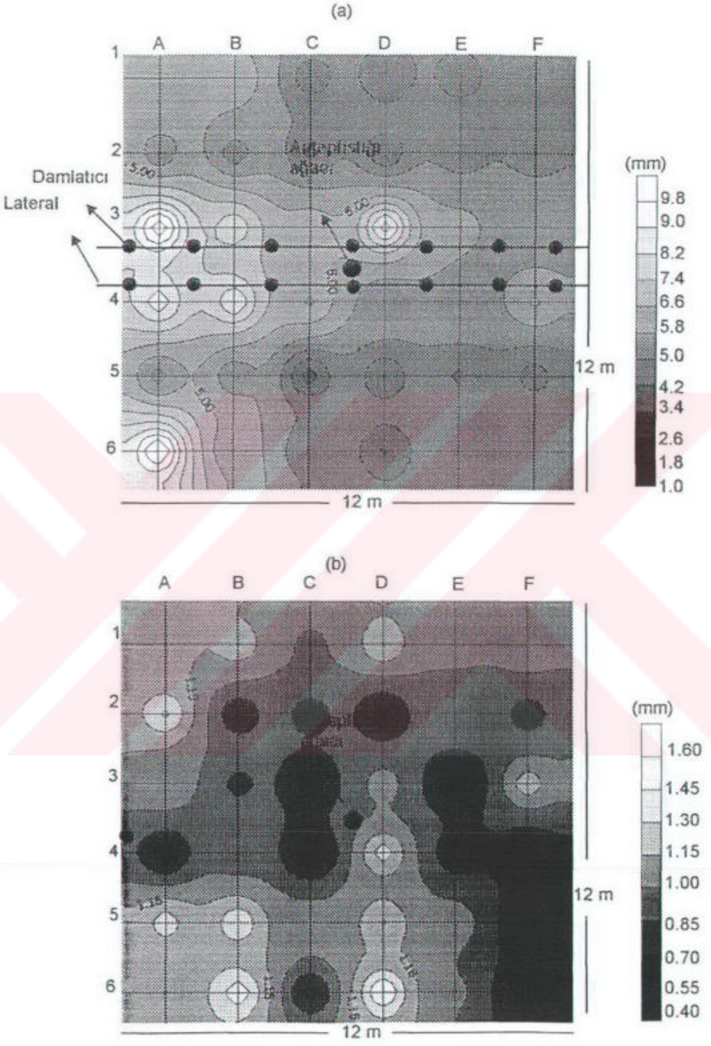

Şekil 4.2. Izgara köşelerindeki 100 cm' lik toprak katmanından 7N$_3$90 (a) ve "geleneksel" (b) konularından evapotranspirasyon yoluyla oluşan toprak su kayıpları

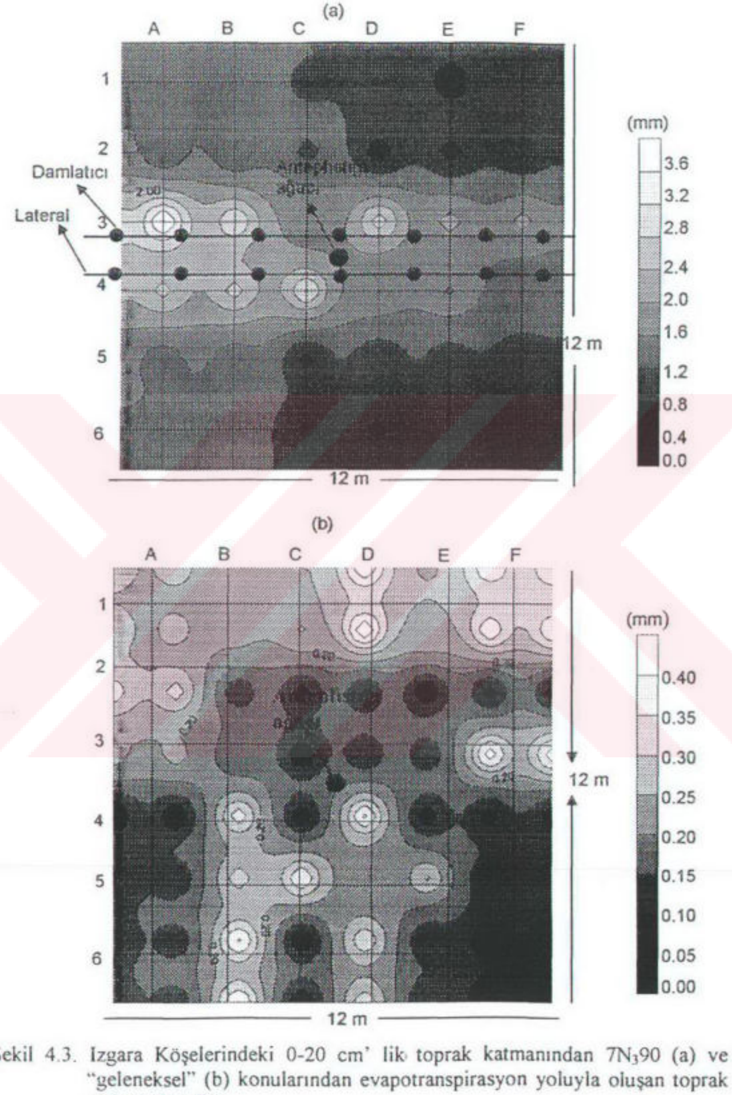

Şekil 4.3. Izgara Köşelerindeki 0-20 cm' lik toprak katmanından $7N_390$ (a) ve "geleneksel" (b) konularından evapotranspirasyon yoluyla oluşan toprak su kayıpları (b)

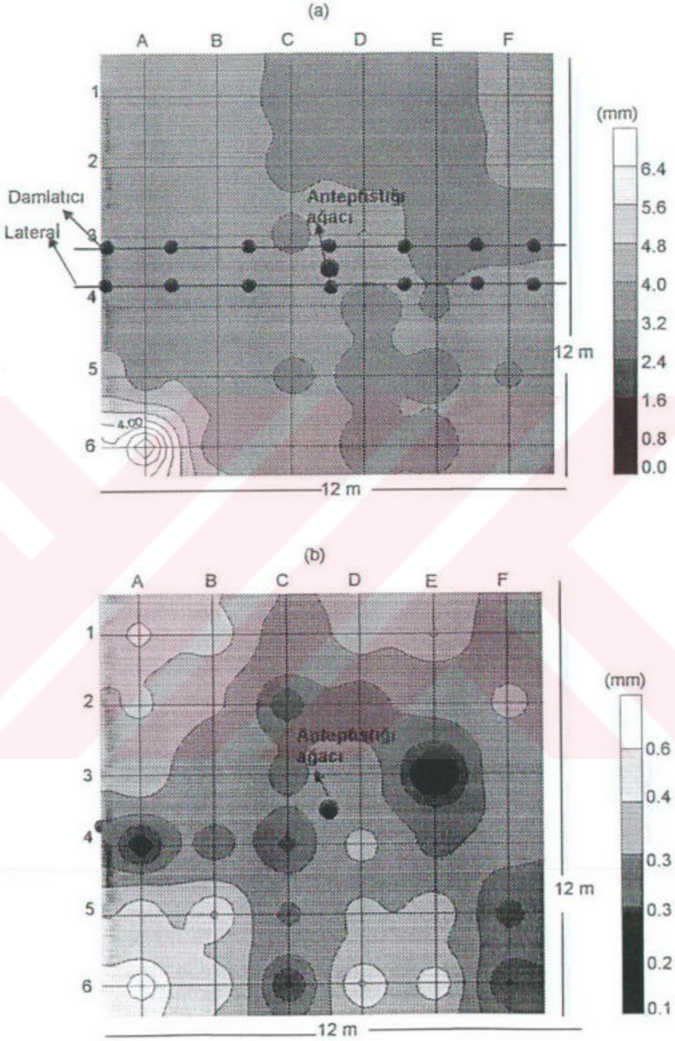

Şekil 4.4. Izgara köşelerindeki 20-40 cm' lik toprak katmanından $7N_390$ (a) ve "geleneksel" (b) konularından evapotranspirasyon yoluyla oluşan toprak su kayıpları

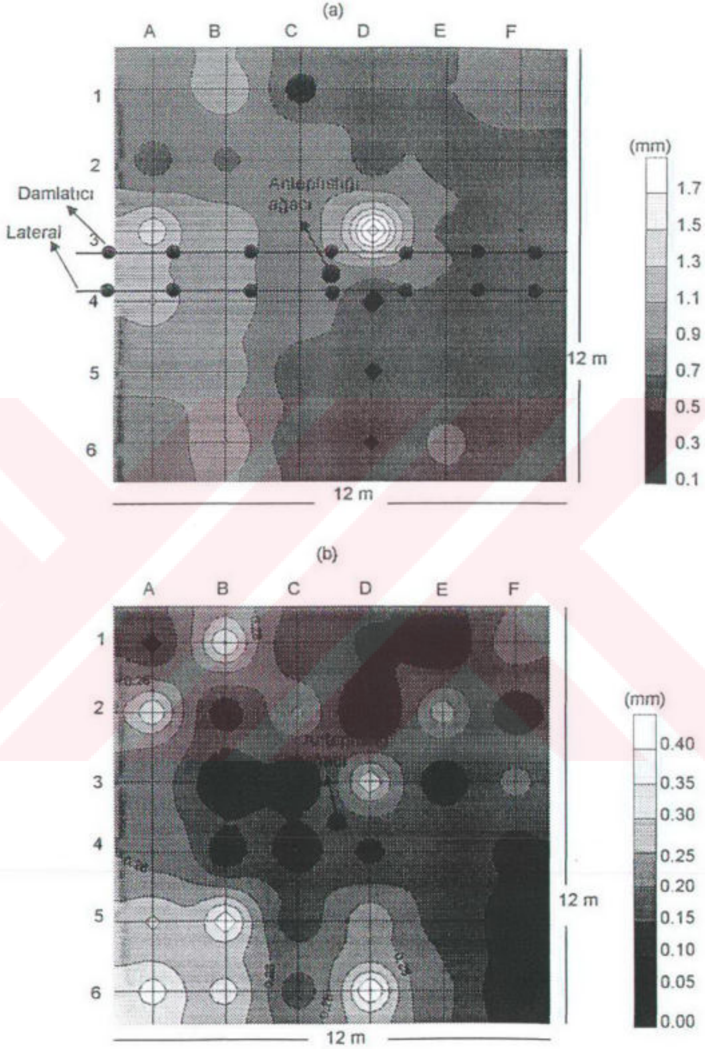

Şekil 4.5. Izgara köşelerindeki 40-60 cm' lik toprak katmanından $7N_390$ (a) ve "geleneksel" (b) konularından evapotranspirasyon yoluyla oluşan toprak su kayıpları

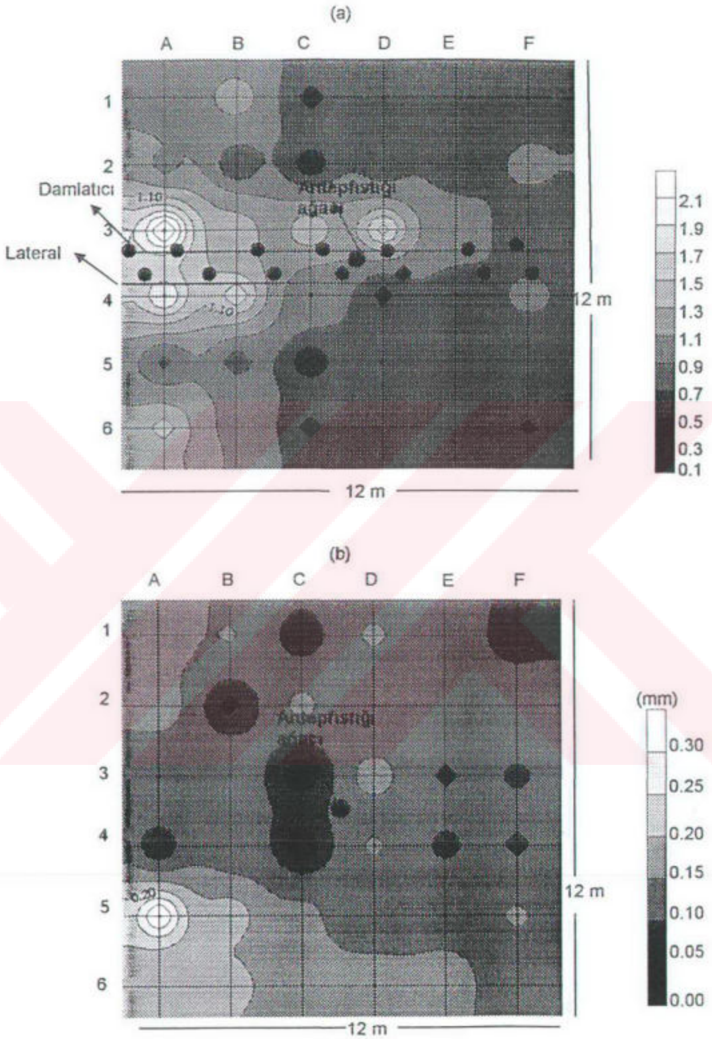

Şekil 4.6. Izgara köşelerindeki 60-80 cm' lik toprak katmanından $7N_390$ (a) ve "geleneksel" (b) konularından evapotranspirasyon yoluyla oluşan toprak su kayıpları

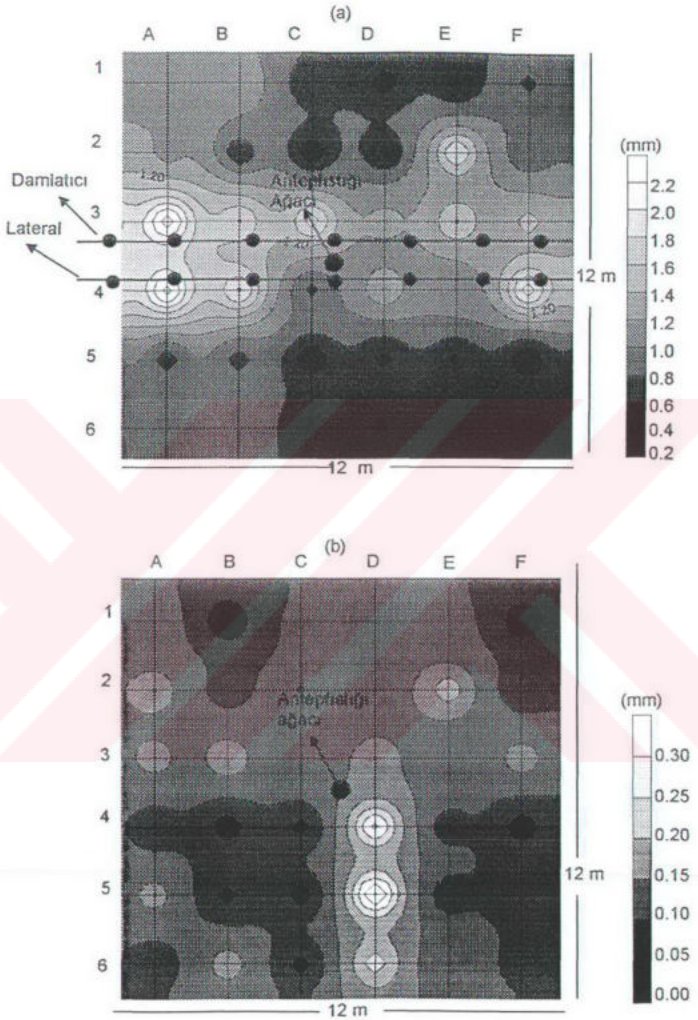

Şekil 4.7. Izgara köşelerindeki 80-100 cm' lik toprak katmanından $7N_3 90$ (a) ve "geleneksel" (b) konularından evapotranspirasyon yoluyla oluşan toprak su kayıpları

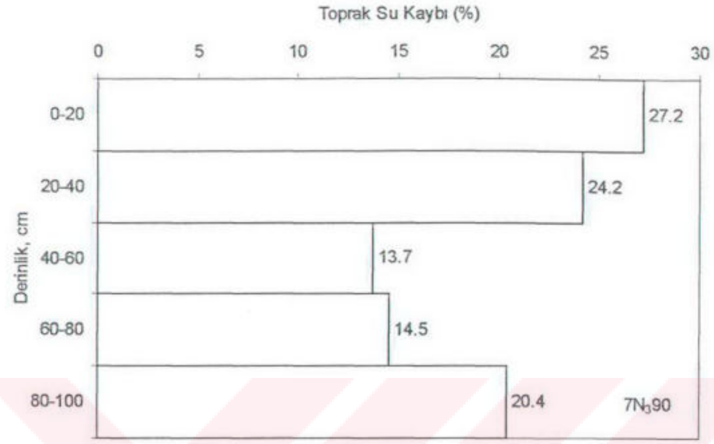

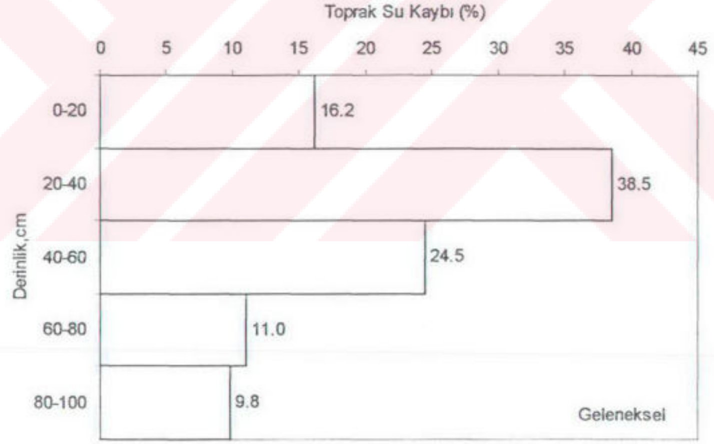

Şekil 4.8. Deneme alanı topraklarının 100 cm' lik kök derinliğinde farklı toprak katmanlarından $7N_390$ ve "geleneksel" konularında meydana gelen ET kayıpları

4.2 Bitki Katsayıları (K_c)

Bitki katsayısı (K_c) değerleri, $7N_390$ konusu için hesaplanmış ve ortalama değerler Şekil 4.9 da gösterilmiştir.

Genel olarak, K_c değerleri, Mart ayından Temmuz ayına kadar artmış; bu aydan sonra Kasım ayına doğru azalmıştır. Konu edilen en yüksek ve en düşük K_c değerleri, yıllara göre değişmekle birlikte, ortalama olarak Temmuz ayında 1.51, Kasım ayında ise 0.39 olarak saptanmıştır. K_c' deki bu tavır, Goldhamer ve ark. (1985) ile Kanber ve ark. (1992) tarafından verilen sonuçlarla uyuşmakla birlikte, sunulan çalışmadan elde edilen K_c değerleri, bir miktar yüksek bulunmuştur.

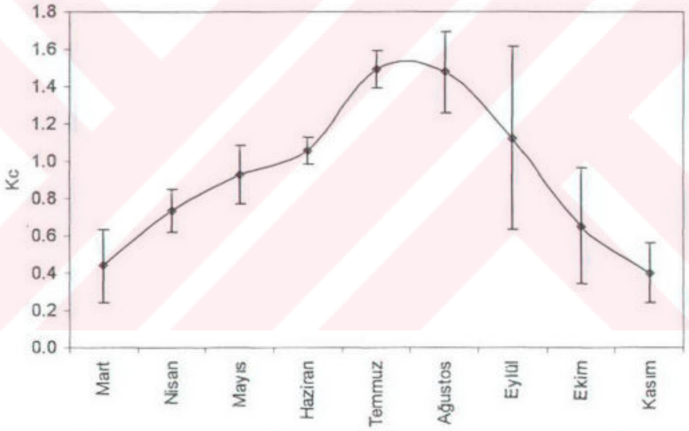

Şekil 4.9. Antepfistığında aylık ortalama K_c değerleri (Veriler, 4 yıllık ortalama değerleri (n = 4), düşey hata çubukları ise ± standart sapmayı göstermektedir.)

Doorenbos ve Pruitt (1977), büyüme dönemi ortasında K_c değerlerinin 1.1-1.2' ye kadar ulaşabileceğini, antepfistığında K_c' nin kestirilebilmesi için bahçede sınır katmanı iletkenliği ve rüzgar hızı gibi etmenlerin etkin olmaları nedeniyle ağacın taç yapısı, yaşı, yüksekliği gibi değişkenlerin çok iyi değerlendirilmesi

gerektiğini belirtmişlerdir. Kurak ve yarı kurak bölgelerde, aşırı güneş enerjisi, uzun gün, sıcak ve kuru hava koşulları nedeniyle K_c değerleri yüksek bulunabilmektedir (Doorenbos ve Pruitt, 1984; Allen ve ark., 1998). Goldhamer (1995), eğer bahçe, hakim rüzgar yönünde çıplak toprak tarafından sınırlandırılmış ise, bölgeye adveksiyon yoluyla fazladan enerji girişi olmasından dolayı ET_c değerlerinin artabileceğini, dolayısıyla K_c değerlerinin yüksek çıkabileceğini belirtmiştir.Ayrıca ET_o kestiriminde kullanılan PM eşitliğinin adveksiyon etkisini dikkate almadığı ve yörede alt-kestirim verdiği belirlenmiştir (Steduto ve ark., 1996). Bitki su tüketimi ve enerji girişi ilişkisini esas alan benzer düşünceler, Allen ve ark. (1998) tarafından da açıklanmıştır. Diğer taraftan Kanber ve ark. (1986), SCS-Blaney-Criddle yöntemiyle hesaplanmış K_c değerlerinin, bu denemede Penman-Monteith yöntemiyle elde edilen K_c değerlerinden daha düşük olduğunu bulmuşlardır. Buradan yüksek K_c değerlerinin eldesi, kullanılan ET_o yönteminden; ET_c değerlerinin yüksekliğinden kaynaklamış olabilir.

4.3 Gübreleme

Çizelge 4.6, 4.7, 4.8 ve 4.9' da saf madde olarak konulara verilen gübre miktarlarına ilişkin sonuçlar görülmektedir. Sulama aralıklarının 7 gün olduğu konulara, yalnızca azot verilmiş, 14 günlük sulamalarda ise, tüm konulara azota ek olarak fosfor ve potasyum gübreleri de uygulanmıştır.

Çizelgelerin incelenmesinden görüleceği gibi, konulara uygulanan gübre (NPK) miktarları, sulama aralıkları ve bitki-pan katsayılarına bağlı olarak değişmiş ve "Geleneksel" konusundan farklılıklar göstermiştir. Deneme yıllarında, en yüksek azot miktarı, lateral hatları tarafından ıslatılan 244 m^2'lik alana göre, 23.3 ve 16.7 g/m^2 ile, en yüksek sulama suyu uygulanan $7N_390$ konusuna verilmiştir. Fosforlu ve potasyumlu gübreler, sulama konularının tümüne eşit miktarlarda uygulanmıştır. En fazla fosforlu ve potaslı gübre, 2000 yılında 6.4 g/m^2 ve 4.3 g/m^2 ile uygulanmıştır.

Çizelge 4.6. Deneme Konularına Verilen Azot, Fosfor ve Potasyum Miktarları (1999)

Konular	IR mm	N g/m^2	P g/m^2	K g/m^2
7$N_0$60	665.7	0.0	5.5	3.6
7$N_0$90	998.6	0.0	5.5	3.6
7N_1 60	665.7	6.7	5.5	3.6
7N_1 90	998.6	10.0	5.5	3.6
7N_2 60	665.7	10.0	5.5	3.6
7N_2 90	998.6	15.0	5.5	3.6
7N_3 60	665.7	13.3	5.5	3.6
7N_3 90	998.6	20.0	5.5	3.6
14$N_0$60	364.5	0.0	5.5	3.6
14$N_0$90	546.7	0.0	5.5	3.6
14N_1 60	364.5	3.6	5.5	3.6
14N_1 90	546.7	5.5	5.5	3.6
14N_2 60	364.5	5.5	5.5	3.6
14N_2 90	546.7	8.2	5.5	3.6
14N_3 60	364.5	7.3	5.5	3.6
14N_3 90	546.7	10.9	5.5	3.6
Geleneksel[a]	-	6.8	8.1	5.4

[a] Azot hesaplamaları, ortalama ağaç örtü alanına göre yapılmıştır.

Çizelge 4.7. Deneme Konularına Verilen Azot, Fosfor ve Potasyum Miktarları (2000)

Konular	IR mm	N g/m^2	P g/m^2	K g/m^2
7$N_0$60	776	0	6.4	4.3
7$N_0$90	1164	0	6.4	4.3
7N_1 60	776	7.8	6.4	4.3
7N_1 90	1164	11.6	6.4	4.3
7N_2 60	776	11.6	6.4	4.3
7N_2 90	1164	17.5	6.4	4.3
7N_3 60	776	15.5	6.4	4.3
7N_3 90	1164	23.3	6.4	4.3
14$N_0$60	425	0.0	6.4	4.3
14$N_0$90	638	0.0	6.4	4.3
14N_1 60	425	4.3	6.4	4.3
14N_1 90	638	6.4	6.4	4.3
14N_2 60	425	6.4	6.4	4.3
14N_2 90	638	9.6	6.4	4.3
14N_3 60	425	8.5	6.4	4.3
14N_3 90	638	12.8	6.4	4.3
Geleneksel	-	6.8	8.1	5.4

Çizelge 4.8. Deneme Konularına Verilen Azot, Fosfor ve Potasyum Miktarları (2001)

Konular	IR mm	N g/m^2	P g/m^2	K g/m^2
$7N_0 60$	617	0.0	4.9	3.2
$7N_0 90$	906	0.0	4.9	3.2
$7N_1$ 60	617	6.2	4.9	3.2
$7N_1$ 90	906	9.1	4.9	3.2
$7N_2$ 60	617	9.3	4.9	3.2
$7N_2$ 90	906	13.6	4.9	3.2
$7N_3$ 60	617	12.3	4.9	3.2
$7N_3$ 90	906	18.1	4.9	3.2
$14N_0 60$	324	0.0	4.9	3.2
$14N_0 90$	467	0.0	4.9	3.2
$14N_1$ 60	324	3.2	4.9	3.2
$14N_1$ 90	467	4.7	4.9	3.2
$14N_2$ 60	324	4.9	4.9	3.2
$14N_2$ 90	467	7.0	4.9	3.2
$14N_3$ 60	324	6.5	4.9	3.2
$14N_3$ 90	467	9.3	4.9	3.2
Geleneksel	-	6.8	8.1	5.4

Çizelge 4.9. Deneme Konularına Verilen Azot, Fosfor ve Potasyum Miktarları (2002)

Konular	IR mm	N g/m^2	P g/m^2	K g/m^2
$7N_0 60$	577	0.0	4.2	2.8
$7N_0 90$	837	0.0	4.2	2.8
$7N_1$ 60	577	5.8	4.2	2.8
$7N_1$ 90	837	8.4	4.2	2.8
$7N_2$ 60	577	8.7	4.2	2.8
$7N_2$ 90	837	12.6	4.2	2.8
$7N_3$ 60	577	11.5	4.2	2.8
$7N_3$ 90	837	16.7	4.2	2.8
$14N_0 60$	311	0.0	4.2	2.8
$14N_0 90$	437	0.0	4.2	2.8
$14N_1$ 60	311	3.1	4.2	2.8
$14N_1$ 90	437	4.4	4.2	2.8
$14N_2$ 60	311	4.7	4.2	2.8
$14N_2$ 90	437	6.6	4.2	2.8
$14N_3$ 60	311	6.2	4.2	2.8
$14N_3$ 90	437	8.7	4.2	2.8
Geleneksel	-	6.8	8.1	5.4

4.4 Bitki Büyüme Özellikleri

Denemenin son iki yılında, deneme ağaçlarından iki yinelemeli olarak ve her bir yinelemedeki konulardan rastgele seçilen üç ağaçtan 20 yaprak örneği alınmıştır. Buradan elde edilen yaprak ağırlığı, yaprak eni, yaprak boyu, ağaç başına yaprak sayısı, gerçek yaprak alanı, toplam yaprak alanı ile ağaç yüksekliği, taç hacmi, ana ve yan dalların sayısı, örtü alanı gibi ağacın bazı büyüme özellikleri belirlenerek Çizelge 4.10- 11 de gösterilmiştir.

Çizelge 4.10 ve 11' in incelenmesinden görüleceği gibi; 2001 yılında yaprak ağırlığı, 0.550 g (geleneksel), 0.375 g ($7N_360$), sulanan konular için ortalama 0.436 g ve azotun uygulanmadığı, N_o konusunda ise 0.430 g arasında değişmiştir. Geleneksel konusuna göre, yaprak ağırlığı sulanan konularda, 2001 yılında % 21, azot uygulanmayan konularda ise % 22 düşmüştür. 2002 yılında ise, sulanan konularda % 0.3, azot uygulanmayan konularda % 5 oranında azalma saptanmıştır.

Taç gelişimi yönünden sulama konuları incelendiğinde, sulanan konulara ilişkin ortalama taç hacmi, 2001 yılında, 36.51 m^3 iken, 2002 yılında % 10 artarak 40.17 m^{3}' e ulaşmıştır. Sulanan ve azot uygulanan konularda "Geleneksel" konuya göre taç gelişimi, 2001 yılında % 3.7 (su) ve % 2 (azot) artarken, 2002 yılında bu artışlar % 11.6 ve % 9.7' ye ulaşmıştır. Bitki taç gelişimi yönünden 2002 yılında sulanan ve azot uygulanan konularda "Geleneksel" konusuna göre 3.13 kat ve 4.85 kat (azot) artış göstermiştir. Yıllara bağlı olarak farklılıkların yüksek bulunması, uygulamaların yığışımlı etkisine bağlanabilir.

Örtü alanı gelişimi yönünden yapılan analizde, 2001 yılında en yüksek 35.5 m^2, ortalama örtü alanı 29.4 m^2 iken, 2002 yılında ise sırasıyla 38.3 m^2 ile 32.1 m^2 olarak bulunmuştur. Sulanan konular ortalaması 2001 yılında 31.5 m^2, 2002 yılında ise 30.05 m^2; bu değerler "Geleneksel" konusunda sırasıyla 27.7 m^2 ile 26.31 m^2 olarak bulunmuştur. Sulanan konularda ortalama örtü alanı, "Geleneksel" konusuna göre, her iki yılda da 1.14 kat artmıştır. Analizlerin yapıldığı 2001 ve 2002 yıllarında ortalama örtü alanı 30.7 m^2 olarak bulunmuştur.

Ağaç tacının örtü %' sini bulmak için, ağaç başına gölgeleme değerleri, bir ağaca ayrılan alana (100 m^2) oranlanmıştır. Ortalama örtü %'si olarak bulunan 30.75

değeri, denemede sulama suyu miktarlarını hesaplamada kullanılan % 30 ıslatma yüzdesinin doğru olduğunu göstermektedir.

Yaprak alan indeksi hesaplamaları için, antepfıstığında ağaç başına ortalama toplam yaprak alanı, 2001 yılında 74 m^2 iken, 2002 yılında 205 m^2 olarak kestirilmiştir. Ağaç başına ayrılan toplam alan 100 m^2 olduğundan, yaprak alan indeksi 0.74 ve 2.05 olarak hesaplanmıştır. 2002 yılında LAI, 2001 yılına göre 2.77 kat daha yüksek bulunmuştur. LAI ile ET ve LAI ile bitki katsayısı (K_c) arasında çok sıkı bir ilişki vardır (Kırnak 1999). Yaprak gelişimi, su eksikliğine karşı oldukça duyarlıdır. Goldhamer ve ark. (1985), antepfıstığında yaprak alanının, sulama düzeyleri ile doğrudan ilişkili olduğunu, sulama dönemi içinde uygulanan su düzeyleri arttıkça, yaprak alanının da arttığını belirtmişlerdir. Muntubani ve ark. (1997), verim yılındaki antepfıstığı ağacının yıllık sürgünlerinin toplam yaprak alanlarının meyvesiz ağaçtan daha fazla olduğunu, çünkü meyveli ağaç sürgünleri ortalama 8 yapraktan oluşurken, meyvesiz ağacın sürgünlerinin ortalama 6 yapraktan oluştuğunu belirtmektedirler. Denemeye ilişkin bulgularla, araştırmacıların sonuçları birbirleriyle uyumlu olduğu görülmektedir.

Çizelge 4. 10. Antepfıstığında Ölçülen/Kestirilen Bazı Büyüme Özellikleri (2001)

Konular	OYA g	OYG cm	OYU cm	OGYA[a] cm^2	ADS adet	YDS adet	TYS adet/ağaç	TYA m^2/ağaç	AY m	GY m	TY m	TG m	OÖG m	TH m^3	ÖA m^2
$7N_060$	0.455	4.75	7.02	16.85	3	58	29893	50.37	4.33	1.45	2.88	6.30	6.30	50.3	31.2
$7N_090$	0.375	4.56	6.70	20.05	3	43	34268	68.71	4.09	1.45	2.64	5.88	5.88	40.4	27.8
$7N_160$	0.455	4.90	6.94	23.32	3	31	35183	82.05	3.88	1.53	2.35	6.18	6.18	27.2	30.1
$7N_190$	0.480	5.09	7.75	24.55	3	39	30360	74.53	4.08	1.50	2.58	6.00	6.00	37.5	28.4
$7N_260$	0.440	4.97	6.81	22.23	4	38	37748	83.89	3.98	1.40	2.58	6.50	6.50	36.6	33.6
$7N_290$	0.440	4.96	6.94	23.83	3	51	39386	93.84	3.93	1.48	2.45	6.05	6.05	30.9	28.7
$7N_360$	0.375	4.82	6.46	24.40	4	53	38484	93.90	4.38	1.70	2.68	6.88	6.88	40.1	35.5
$7N_390$	0.460	4.89	7.00	25.90	3	59	49591	128.44	4.23	1.55	2.68	6.70	6.70	40.9	35.3
$14N_060$	0.500	5.16	7.47	26.75	3	40	27472	73.49	3.90	1.65	2.25	6.23	6.23	23.9	30.6
$14N_090$	0.390	4.74	6.57	21.50	3	46	22644	48.68	4.05	1.43	2.63	6.65	6.65	38.1	34.8
$14N_160$	0.390	4.96	6.99	22.12	3	41	22185	49.07	3.88	1.48	2.40	5.45	5.45	29.1	23.3
$14N_190$	0.405	4.64	6.91	21.58	3	42	19488	42.05	4.15	1.53	2.63	6.40	6.40	37.9	32.2
$14N_260$	0.475	5.13	7.10	21.45	3	43	32235	69.14	4.30	1.63	2.68	6.70	6.70	40.6	35.4
$14N_290$	0.395	4.88	6.53	23.58	3	32	30944	72.95	3.85	1.53	2.33	6.55	6.55	26.4	33.9
$14N_360$	0.460	4.86	7.04	25.30	3	33	25332	64.09	4.28	1.65	2.63	6.48	6.48	38.7	32.9
$14N_390$	0.480	5.18	7.15	25.53	3	44	34975	89.29	4.30	1.53	2.78	6.25	6.25	45.6	30.7
Geleneksel	0.550	4.88	7.10	23.35	3	38	32272	75.36	4.08	1.53	2.55	5.93	5.93	35.2	27.7

OYA: ort.yap.ağır.; OYG: ort. yap.geniş.; OYU: ort. yap.uzun.; OGYA: ort. ger. yap. alanı; ADS: ana dal say.; YDS: yan dal sayısı; TYS: ağaç başına top. yap.say.; TYA: top. yap.alanı; AY: ağaç yük.;GY: gövde yük.; TY: taç yük.; TG: taç geniş.; OÖG: ort. örtü geniş.; TH: taç hacmi; ÖA: örtü alanı.

[a]Ortalama Gerçek Yaprak Alanı değerleri; gerçek yaprak alanı değerleri ile yaprak uzunluğu ve yaprak genişliğinin çarpımından elde edilen yaprak indeksi arasındaki ilişkiyi veren YA= 1.2408 İND+4.3802 eşitlik kullanılarak hesaplanmıştır.

Çizelge 4.11. Antepfistığında Ölçülen/Kestirilen Bazı Büyüme Özellikleri (2002)

Konular	OYA g	OYG cm	OYU cm	OGYA[a] cm^2	ADS adet	YDS adet	TYS adet/ağaç	TYA m^2/ağaç	AY m	GY m	TY m	TG m	OÖG m	TH m^3	ÖA m^2
$7N_0 60$	0.540	5.43	7.65	55.9	3	53	39549	221.03	4.36	1.4	3.0	6.5	6.50	57.1	32.7
$7N_0 90$	0.550	5.52	7.45	55.4	3	42	27616	152.91	4.09	1.5	2.6	5.8	5.83	40.3	27.5
$7N_1 60$	0.520	5.22	7.44	52.5	3	29	39967	209.97	4.15	1.5	2.7	5.8	5.75	40.2	26.0
$7N_1 90$	0.590	5.32	7.82	56.0	3	37	23202	129.89	4.03	1.5	2.6	5.9	5.85	37.5	27.1
$7N_2 60$	0.610	5.73	7.94	61.1	4	36	30455	186.14	4.08	1.4	2.7	6.5	6.50	41.3	34.0
$7N_2 90$	0.570	5.51	7.26	54.2	3	47	38180	207.12	3.95	1.4	2.6	6.0	6.00	34.8	28.3
$7N_3 60$	0.630	5.79	8.31	64.1	4	52	45868	294.14	4.20	1.7	2.5	7.0	6.98	33.1	38.3
$7N_3 90$	0.800	6.3	8.65	72.1	3	54	48583	350.32	4.15	1.6	2.6	6.3	6.33	37.2	31.4
$14N_0 60$	0.630	5.87	7.78	61.2	3	38	25576	156.43	3.90	1.7	2.3	5.9	5.85	23.8	27.0
$14N_0 90$	0.630	6.09	7.73	62.7	3	45	51802	324.62	4.23	1.4	2.8	6.2	6.20	47.5	31.3
$14N_1 60$	0.550	5.55	7.96	59.2	3	38	29802	176.50	3.90	1.4	2.5	5.8	5.75	34.1	26.1
$14N_1 90$	0.640	5.96	7.76	61.7	3	41	31964	197.34	4.20	1.4	2.8	5.9	5.90	45	27.6
$14N_2 60$	0.720	6.15	8.66	70.4	3	41	24604	173.25	4.35	1.6	2.7	6.2	6.23	42.6	30.5
$14N_2 90$	0.750	5.83	8.74	68.1	3	30	27884	189.79	3.90	1.3	2.6	6.1	6.05	36.6	29.4
$14N_3 60$	0.610	5.76	7.93	61.1	3	32	23735	144.95	4.28	1.6	2.7	6.4	6.43	41.6	32.4
$14N_3 90$	0.590	5.89	7.9	62.1	3	42	30940	192.02	4.40	1.5	2.9	6.3	6.30	50	31.2
Geleneksel	0.620	5.81	8.03	57.1	3	35	29994	171.55	4.07	1.4	2.6	5.8	5.79	36	26.3

OYA: ort.yap.ağır.; OYG: ort. yap.geniş.; OYU: ort. yap.uzun.; OGYA: ort. ger. yap. alanı; ADS: ana dal say.; YDS: yan dal sayısı; TYS: ağaç başına top. yap.say.; TYA: top. yap.alanı; AY: ağaç yük.;GY: gövde yük.; TY: taç yük.; TG: taç geniş.; OÖG: ort. örtü geniş.; TH: taç hacmi; ÖA: örtü alanı.

[a]Ortalama Gerçek Yaprak Alanı değerleri; gerçek yaprak alanı değerleri ile yaprak uzunluğu ve yaprak genişliğinin çarpımından elde edilen yaprak indeksi arasındaki ilişkiyi veren YA= 1.2408 İND+4.3802 eşitlik kullanılarak hesaplanmıştır.

4.4.1 Karagöz Dökülme Oranı (KDO) ve Verim - Karagöz İlişkisi

Deneme yıllarında karagöz (meyve gözü) dökülme oranlarını (KDO) ve verim ile ilişkisini belirlemek amacıyla, iki dönemde karagöz sayımları yapılmıştır. Antepfistığında karagöz miktarları, bir sonraki yılın meyve verimini doğrudan etkilediğinden dolayı, karagöz döküm oranı-verim ilişkisi, bu yaklaşımla değerlendirilmiştir. Sayımın yapıldığı yılın KDO ile bir sonraki yılın verim değerleri karşılaştırılmıştır. Deneme konularından elde edilen karagöz dökülme oranlarına ilişkin değerler, KDO-verim ilişkileri Şekil 4.10' da, yıllara göre KDO oranları Ek Şekil 1-2' de, toplu olarak, Şekil 4.11' de, gösterilmiştir.

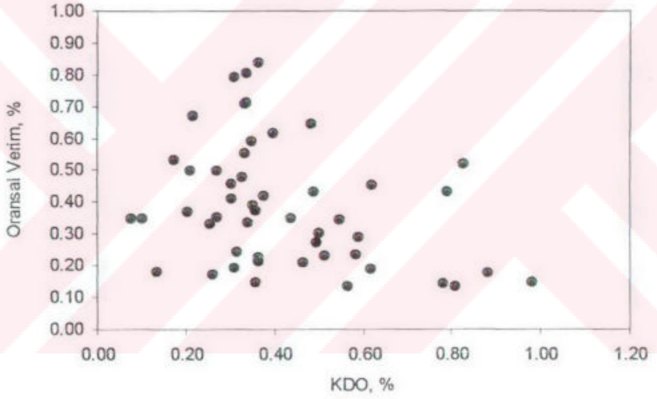

Şekil. 4.10. Deneme konularına ilişkin verim-KDO ilişkisi

Şekil 4.11' de görüldüğü gibi, deneme yıllarında konulara ilişkin ortalama karagöz dökülme oranları farklı düzeylerde bulunmuştur. Denemenin ilk yılında (1999), konuların karagöz dökülme oranları yaklaşık olarak aynı düzeylerde gerçekleşmiştir. Ortalama karagöz dökülme oranları, deneme yılları boyunca artan bir eğilim göstermiştir. Buna karşı, verim değerlerindeki azalma beklenen düzeyde olmamıştır. Deneme yılları boyunca ortalama karagöz dökülme oranları, 0.35- 0.43- 0.48 ve 0.59 olarak hesaplanmıştır (Ek Çizelge 29-32).

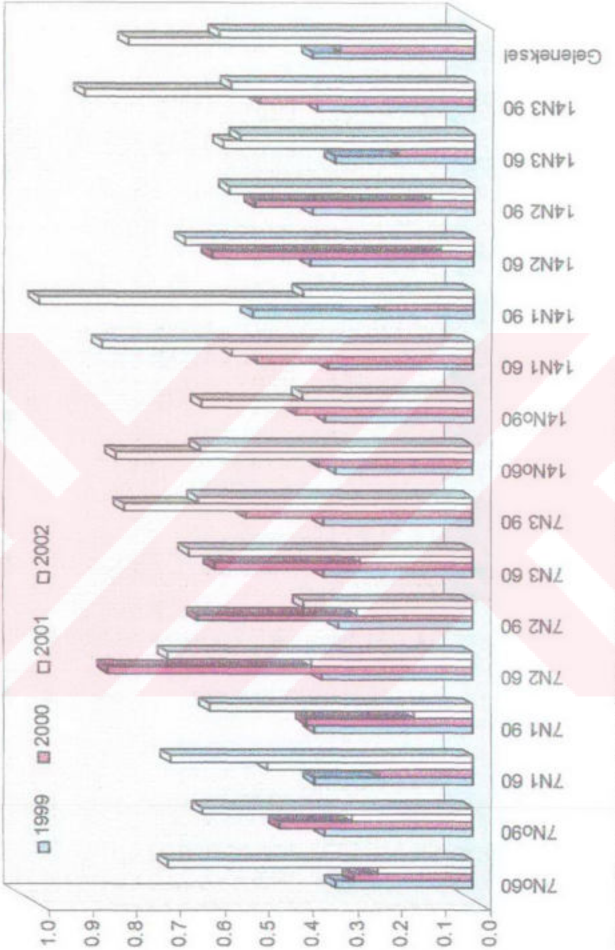

Şekil 4.11. Deneme konularına ilişkin KDO' nun yıllara göre değişimi

KDO' da 1999-2000 yıllarında meydana gelen % 18.6' lık bir artışa karşın, verimde % 15 oranında bir azalma meydana gelirken, 2000-2001 yıllarında KDO' da % 11'lik artış oranı, verimde beklenenden daha büyük (% 36) bir azalmaya neden olmuştur. Buradan periyodisitenin, yalnızca karagöz dökülmesinin bir sonucu olmayıp, bitki genetiği, bitki fizyolojisi ve çevresel faktörlerin de bir sonucu olduğu söylenebilir.

Karagöz dökülme oranları ile oransal verim değerleri arasında, ikinci dereceden bir ilişkinin olabileceği söylenebilir. Şekil 4.10' nun incelenmesinden anlaşılacağı gibi, yaklaşık % 27'ye kadar karagöz dökülme oranları, verimde herhangi bir azalmaya neden olmamakta, bu noktadan sonra KDO artışıyla birlikte oransal verimin düştüğüne ilişkin genel bir eğilimin gözlendiği söylenebilir. Tekin (1992), antepfıstığı ağaçlarının fazla gübrelemeye karşın karagöz dökülmelerinin yüksek oranda gerçekleştiğini belirtmiştir. Bu durumu, meyve yükünün fazlalığı yanında, ağaç bünyesindeki hormonal dengenin aşırı meyve yüküne bağlı olarak bozulmuş olmasına ve genetik etmenlere bağlamıştır. Araştırmacı, periyodisitenin nedeni olarak, verimin yüksek olduğu yıllarda, meyve yükünün yanı sıra, kültürel bakım koşullarının yetersizliğinin ve kısıtlı toprak su kapsamı düzeylerinin meyve gözü dökümüne etkili olduğunu açıklamıştır. Tekin ve Güzel (1993), ağacın verim düzeyi arttıkça periyodisitenin de arttığını ancak yeterli ve dengeli bir gübreleme ile meyve gözü dökümünün yaklaşık % 38 oranında azaltılabileceğini belirtmişlerdir. Lovatt ve Ferguson (1997), antepfıstığında periyodisitenin oluş mekanizmasının tanımlandığını ancak, fizyolojisinin anlaşılamadığını belirterek; karagöz dökülmesinin üç nedenden kaynaklanabileceğini yazmışlardır. Bunları; meyve iç doldurma zamanında (Haziran-Temmuz) azot rekabeti, meyve gözü dökümünü artıran absisik asit konsantrasyonunun meyve gözlerinde artması ve gözlerde dökülmeyi engelleyen hormonlardan iki sitokinin (isopentyladenine ve zeatin riboside) derişimlerinin azalması olarak sıralamışlardır. Biüret ürenin düşük derişiminde 6-Benziladenine ile birlikte ağaç tacına uygulanmasıyla, meyve dolum dönemi olan kritik dönemde, fazladan azot sağlanmış ve bu yolla gözlerde isopentyladenine derişimi artırılarak göz dökülmesi, 1.6 kat azaltılmıştır. Takeda ve Crane (1980), periyodisitenin meyve gözü dökülmesi yoluyla, meyve içinin hızlı

geliştiği dönemde oluştuğunu ve geriye kalan meyve gözlerinin bir sonraki yılda meyveye dönüştüğünü belirtmişlerdir. Kritik dönem olan meyvenin iç doldurma döneminde, meyveli sürgünlerin kabuk ve odun dokularında ne karbonhidrat ve ne de azot düzeylerinin, periyodisite yılındaki sürgünlerdeki düzeylerinden farklı olmadığını rapor etmişlerdir. Diğer bir deyimle, verim yılındaki meyve gözlerinde bulunan azot düzeyleri, periyodisite yılındakinden farklı değildir. Periyodisitenin, meyvenin iç doldurma döneminde (Temmuz-Ağustos) meyve gözlerinin dökülmesi sonucu ortaya çıktığını belirten Crane ve ark. (1976), bu olayın karbonhidrat eksikliğinin bir sonucu olarak ortaya çıkmadığını savunmuşlardır. Buna karşı, antepfıstığı yetiştiriciliğinde karşılaşılan en önemli sorunlardan olan periyodisitenin, ağaçlarda meyve yükünün fazla olduğu yıllarda meyve gözlerinin dökülmesi sonucu meydana geldiğini belirten Porlingis (1974), Weinbaum ve Muraoka (1989), dökülmenin en fazla Temmuz ve Ağustos aylarında yoğunlaştığını, bu dönemde meyve içinin hızlı gelişerek büyüdüğü dönemde karbonhidrat kullanma yarışında meyvenin öne çıktığını ve dolayısıyla yeterince karbonhidrat kullanamayan meyve gözlerinin döküldüğünü belirtmektedirler. Marra ve ark. (1997), antepfıstığında periyodisite olayının, iki yılda bir ürün veren diğer bitkilerden çok farklı bir işleyişte olduğunu belirtirken; Crane ve Nelson (1971), antepfıstığı ağacının her yıl fazla sayıda meyve gözü oluşturduğunu, ancak antepfıstığı ağacı tarafından özümlenen asimilasyon ürünlerinin, verim yılında embriyo gelişimi döneminde tüketilmesi sonucu bunların büyük bir kısmının döküldüğünü, bu yolla gelişen bilinmeyen bir mekanizmanın etkisiyle periyodisitenin meydana geldiğini açıklamışlardır.

Meyve gözleri dökümünün embriyo gelişimi ve meyve miktarı kadar, antepfıstığı ağacının yaprak alanı ve kullanılabilir karbonhidrat düzeyi ile de ilişkili olduğu; yetersiz beslenmenin meyve gözü dökümü için tek başına etkili bir neden olmayacağı da Takeda ve Crane (1980) tarafından belirtilmiştir. Durzan (1996), meyve içinin gelişmesinde, dökülen meyve gözleri yoluyla kaybolan azot bileşenlerinin yeniden alımının, öncelikle kökler yoluyla alınan azotun kullanılabilirliğine bağlı olduğunu, ağacın kökleri yoluyla alınan azotun, meyve içi gelişiminde oldukça önemli olduğunu açıklamıştır.

Bu durum, antepfıstığında, fertigasyon çalışmalarında kullanılan azotlu gübrelerin, meyve içi geliştirme döneminde daha etkin yararlanabilmesi için, toprakta daha geç eriyebilen türden seçilmesini veya bu dönemde bitki kök bölgesinin azotlu gübre ile yeterince doygun olmasını zorunlu hale getirmektedir.

4.4.2 Antepfıstığında Periyodisite İndeksinin Belirlenmesi

Deneme konularından elde edilen yıllık ortalama verim değerleri kullanılarak hesaplanan periyodisite indeksine ilişkin sonuçlar Şekil 4.12' de gösterilmiştir.

Şekil 4.12' nin incelenmesinden görüleceği gibi, deneme yılları boyunca su ve azotun farklı düzeylerde uygulandığı konularda hesaplanan periyodisite indeksi değerleri, genel olarak birbirine yakın bulunmuştur. Su ve azot düzeylerindeki farklılıklar, periyodisite indeksi değerlerinde belirgin olarak görülmemiştir. En yüksek periyodisite indeksi değeri, 14 günlük sulama aralığının kullanıldığı ve hiç azot uygulanmayan $14N_060$ konusundan 0.766; en düşük değer ise, 7 günlük sulama aralığında hiç azot uygulanmayan $7N_090$ konusunda 0.501, bütün deneme konularından ortalama olarak 0.604 olarak hesaplanmıştır. Sulama suyu uygulanmayan sadece azot verilen geleneksel konuda periyodisite indeksi ortalama değerden daha yüksek (0.627) bulunmuştur.

P. atlantica üzerine aşılı *Kerman* çeşidi üzerinde farklı budama yöntemlerinin verim, kalite ve periyodisite indeksi üzerine etkisini araştıran Ferguson ve ark. (1995), periyodisite indeksinin budama yöntemlerinde farklı değerler aldığını, diğer bir deyimle, budama yöntemlerinin antepfıstığında periyodisite üzerinde farklı etkiler meydana getirdiğini belirtmiştir. Araştırmacı, antepfıstığında yapılacak yıllık ağır budama uygulamalarının, periyodisiteyi önleyebileceği veya periyodisite şiddetini azaltabileceğini rapor etmiştir.

Wood (1989) pecan cevizi üzerinde yaptığı çalışmada, periyodisite indeksinin düşük olduğu anaçların köklerinde, daha fazla nişasta bulundurduğu, buna karşın, verim değerlerinin daha yüksek olduğunu belirtmektedir. Ak (1998), aynı çeşit içerisinde bile bazı ağaçların mutlak periyodisite, bazılarının ise kısmi periyodisite gösterdiğini açıklamıştır. Sunulan çalışmaya ilişkin bulgular, denemede kullanılan

Uzun çeşidinin kısmi periyodisite gösterdiği ve araştırmacının sonuçlarıyla uyumlu olduğu söylenebilir.

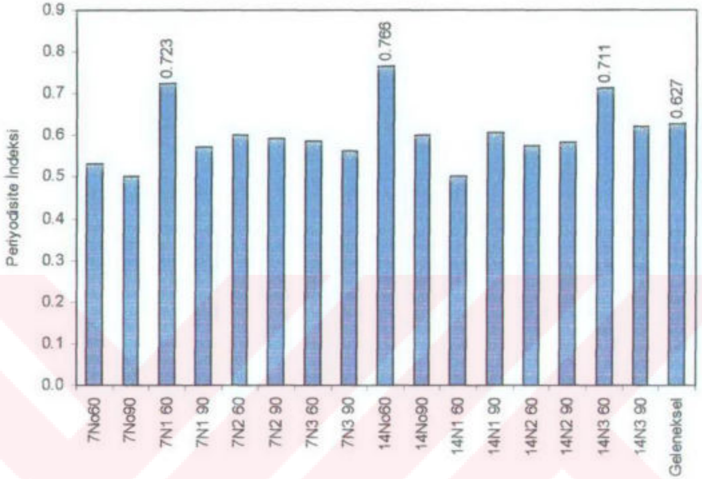

Şekil 4.12. Deneme konularına ilişkin periyodisite indeksi değerleri

Periyodisitenin, genetik, yetiştirme koşulları ve fizyolojik nedenlerden kaynaklandığını belirten Kaşka (2001), özellikle yeni bahçe tesisinde mutlak periyodisite göstermeyen veya aynı çeşit içerisinde düzenli verim veren ağaçların seçilmesini önermektedir.

4.4.3 Antepfıstığı Kök Yoğunluğunun Belirlenmesi

Antepfıstığında kök miktarı, birim hacim toprakta ağırlık olarak belirlenmiş ve elde edilen sonuçlar Şekil 4.13' de gösterilmiştir.

Şekil 4.13'ün incelenmesinden görüleceği gibi, deneme alanı toprak profilinde kök yoğunluğu, çok sulanan konudan az sulanan ve hiç sulanmayan geleneksel konusuna doğru örnekleme yapılarak, kök yoğunluğu incelenmiştir. Sulanan antepfıstığı konusunda kök yoğunlukları, sulanmayan konuya kıyasla,

toprak derinliğine bağlı olarak farklı bulunmuştur. Sulanan konularda, toprak profilinin 60-90 cm'lik katmanlarında, sık sulama koşuluyla kök yoğunluğu artmıştır. Buna karşı, sulanmayan konuda en fazla kök yoğunluğunun, 30-60 cm' de arttığı görülmektedir (Şekil 4.13).

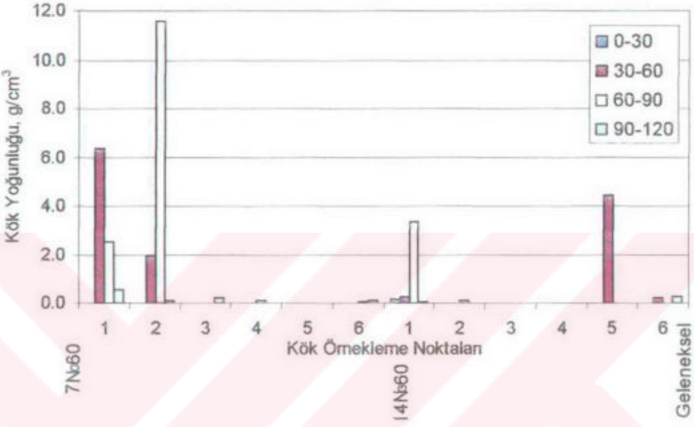

Şekil 4.13. Antepfıstığında farklı derinliklerdeki kök yoğunluğu

Genel olarak, antepfıstığında kök yoğunluğu, özellikle toprağın 30-90 cm'lik katmanında artmaktadır. Bundan sonra kök yoğunluğu azalmaktadır. Toprağın 120 cm derinliğindeki toplam kök yoğunluğunun % 0.5 lik kısmı, ilk 30 cm 'lik katmanda bulunurken, diğer katmanlarda sırasıyla % 40.8, % 54.7 ve % 4.1 olarak hesaplanmıştır. Toplam kök yoğunluğunun % 95.5' lik kısmı, toprağın ilk 30-90 cm'lik katmanında bulunmaktadır.

Goldberg ve ark. (1971), antepfıstığında köklerin, suyun ve minerallerin dağılımını inceledikleri çalışmalarında, damla sulama ile kök gelişiminin azaldığını ve köklerin damlatıcıların çevresinde yoğunlaştığını; kök gelişiminin damlatıcıdan uzaklaştıkça azaldığını bildirmişlerdir. Benzer olarak Kanber ve ark. (1993), kök etkinliğinin sık sulanan konuda, sulanmayan veya az sulanan konuya göre daha yüzlek derinliklerde sınırlandığını açıklamışlardır. Sunulan çalışmada elde edilen bulgular, araştırmacıların bulgularıyla karşıt görünmektedir. Bu durum, kullanılan

sulama yönteminden olduğu kadar, deneme alanı toprak profilinde bulunan kaliş katmanlarından kaynaklanmış olabilir. Sulanmayan konularda, toprak profilinin daha derin katmanlarında kök yoğunluğunun artması da, benzer olarak, üst toprak katmanlarında toprak işleme ve sıcaklık nedeniyle oluşan yüksek evaporasyon sonucu bitki kök bölgesinde yeterince bitki besin maddesi ve su bulunmayışından kaynaklanmış olabilir. Rosecrance ve ark. (1995), antepfistığında meyve iç doldurma dönemindeki kök gelişiminin verim yıllarında periyodisite yıllarına oranla daha düşük olduğunu açıklamışlardır.

4.4.4 Yaprak Su Potansiyelinin Ölçülmesi

Deneme konuları arasından seçilen tam sulanan ve en fazla azot verilen $7N_390$ konusu ile sulanmayan "Geleneksel" konularında yaprak su potansiyeli (Ψ_L) ölçümleri yapılmıştır. Elde edilen sonuçlar Şekil 4.14 - 16' da gösterilmiştir.

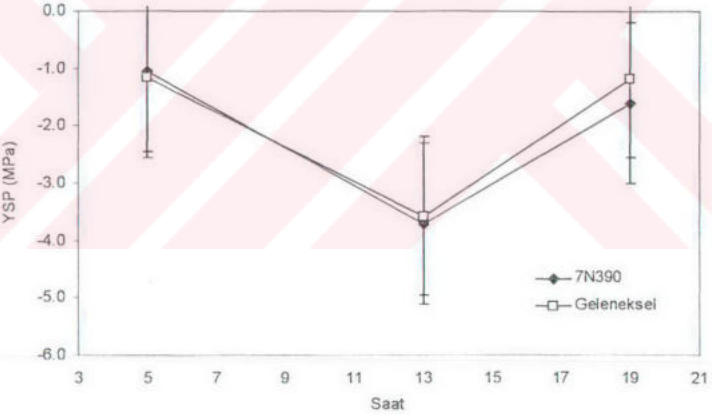

Şekil 4.14. Sulama dönemi başında test konularına ilişkin YSP değerlerinin günlük değişimi (Veriler, saatlık ortalama değerleri (n = 4), düşey hata çubukları ± standart sapmayı göstermektedir.)

Şekillerden anlaşılacağı gibi, sulama dönemi başında (Şekil 4.14), $7N_390$ ve "Geleneksel" konularında YSP değerleri, benzer değişim göstermiştir. Her iki

konuda da Ψ_L değeri, özellikle hava sıcaklığının arttığı öğle saatlerinde -3.7 MPa değerine kadar düşmüştür. Gündoğuşu ve gün batımı saatlerinde ise sıcaklığın düşmesi ile birlikte yeniden yükselerek -1.6 MPa' a ulaşmıştır.

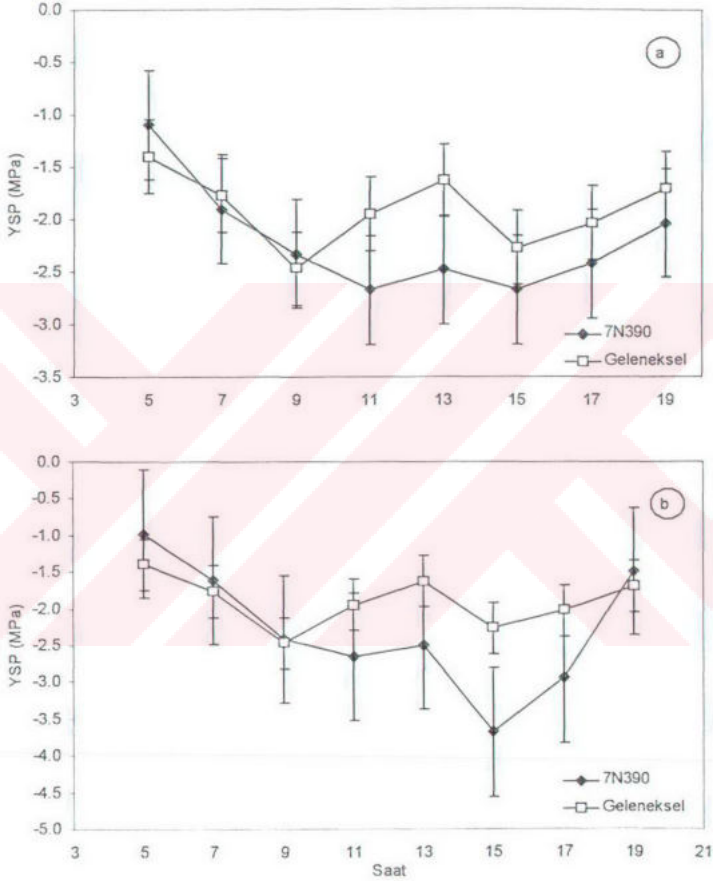

Şekil 4.15. Sulama dönemi içerisinde sulamadan önce (a) ve sulamadan sonra (b) YSP' de meydana gelen değişme(Veriler, saatlık ortalama değerleri (n=4), düşey hata çubukları ± standart sapmayı göstermektedir.)

Sulama dönemi ortasında (12 Ağustos 2002) her iki konuda, sulamadan önce ve sonra, ardışık olarak, 2 saatlik aralıklarla ölçümler yapılmıştır. Sulama öncesi Ψ_L değerleri sabah saatlerinde -1.1 MPa ile -1.4 MPa dolaylarında iken, hava sıcaklığının artmasıyla birlikte (yaklaşık 09:00) "Geleneksel" konuda negatif olarak -2.47 MPa değerine düşmüştür (Şekil 4.15 a). Ancak, aynı saatlerde $7N_390$ konusunda YSP azalmaya devam ederek, saat 11:00 dolaylarında -2.67 MPa değerine ulaşmıştır. Bu noktadan sonra saat 11:00 ile 15:00 arasında hafif dalgalanma ile beraber aynı düzeyde kalmış, bundan sonra artmaya başlamıştır. Sabah saatlerinde, YSP' nin azalmaya başlamasıyla yer değiştiren Ψ_L eğrileri, saat 15:00 ten sonra, gün batımına değin birbirine paralel olarak devam etmiş ve sulanan konu daha düşük bir YSP değerine (-2.03 MPa) ulaşmıştır. Geleneksel konuda sabah saatlerinde (09:00) yaprak su potansiyelinin artmaya başlaması, bitki dokularında bulunan mevcut suyun transpirasyonla oluşan kaybını engelleme isteminden kaynaklanmaktadır.

Benzer olarak, aynı konularda sulamadan sonra da ölçümler yapılmıştır (Şekil 4.15 b). Sulanan ($7N_390$) ve "geleneksel" konularının her ikisinde de Ψ_L değerleri, sabah saatlerinde birbirine paralel olarak azalmış, geleneksel konu saat 09:00 da, -2.47 MPa değerine ulaşmıştır. Bu noktadan sonra artmaya başlamıştır. Ancak, hava sıcaklığının ve buna bağlı olarak güneş enerjisinin artmasıyla birlikte sulama öncesinde olduğu gibi sulanan konuda, azalma devam etmiş, en düşük Ψ_L değeri, -3.68 MPa olarak ölçülmüştür. Bu noktadan sonra Ψ_L değeri artmaya başlamıştır. Hava sıcaklığının düşmeye başladığı akşam saatlerinde Ψ_L değeri, sulanan $7N_390$ konusunda -1.5 MPa' kadar ulaşmaktadır.

Sulama uygulamaları bittikten sonra (03 Ekim 2002) konularda Ψ_L değerlerinin günlük değişimleri, sulama dönemi içerisindekine benzer şekilde gelişmiştir (Şekil 4.16). Sulanan $7N_390$ konusunda, bu kez daha erken saatlerde Ψ_L değeri artmaya başlarken, "Geleneksel" konusunda ise daha geç saatlerde başlamıştır. Her iki konuda sulama sonrası dönemde oluşan en düşük Ψ_L değeri, sırasıyla -3.88 MPa ve -2.67 MPa, en yüksek Ψ_L değeri -2.17 MPa ve -1.40 MPa olarak gerçekleşmiştir. Genel olarak sulanan konularda, sulanmayan geleneksel konusuna göre sürekli bir azalma görülmüştür. Bu da, sulanan konulardaki ağaçların

köklerinin ve dolayısıyla yaprakların sulanmayan ağaçlara göre, sulanır ortama daha fazla koşullandığını ve adapte olduğunu göstermektedir.

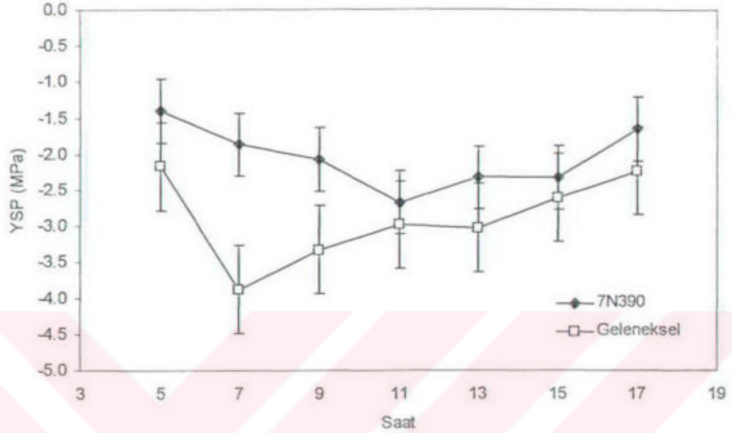

Şekil 4.16. Konuların sulama dönemi sonundaki YSP değerlerinin günlük değişimi (Veriler, saatlık ortalama değerleri (n=4), düşey hata çubukları ± standart sapmayı göstermektedir.)

Goldhamer ve ark. (1986), farklı sulama düzeylerinde yaptıkları YSP ölçümlerinde, yukarıdaki bulguların karşıtı olarak, sulanmayan konularda Ψ_L değerini, -3.59 MPa, tam sulanan konularda ise -1.54 MPa olarak ölçmüşlerdir. Çalışma, 11 yaşındaki genç ve henüz gençlik kısırlığı döneminden yeni çıkmakta olan P. Atlantica anacı üzerine aşılı Kerman çeşidi antepfıstığı ağaçlarında yürütülmüştür. Sunulan çalışmadaki bulgular ise, deneme yıllarından önce sulama yapılmayan, 30 yaşında ve optimum verim çağındaki *P. vera* L. anacı üzerine aşılı Uzun çeşidi antepfıstığı ağaçlarında ölçülmüştür. Aradaki uyumsuzluğun, iklim ve çeşit farklılığı, ağaç taç gelişimi, yaşı, meyve yükü, sulama öncesi dönemde uzun süre su stresi koşullarında kalması gibi etmenlerden kaynaklandığı düşünülebilir. Özmen (2002), verim çağındaki antepfıstığı ağaçlarında yürüttüğü çalışmasında, tam sulanan ve eksik sulanan konularda en düşük Ψ_L değerini sırasıyla -3.2 MPa ve -2.9 MPa olarak ölçmüştür. Goldhamer ve ark. (1985), bitkilerden transpirasyon yoluyla

oluşan su kayıplarının, yapraklarda bulunan stomalar tarafından kontrol edildiğini açıklamışlardır. De Palma ve ark. (1997), sulanan antepfistığında gövde su potansiyeli (GSP- stem water potantial, Ψ_{stem}) değerlerinin, sulanan ve sulanmayan konularda benzer değişim gösterdiklerini belirtmişlerdir. Araştırmacılar bu durumun, bitki büyüme dönemleriyle olduğu kadar, ölçmelerin yapıldığı mevsim içerisindeki iklim değişmelerinden kaynaklandığını açıklamışlardır. Monastra ve ark. (1997), antepfistığı yapraklarının iki yüzünde bulunan stomaları nedeniyle kuraklığa oldukça dayanıklı olduğunu ve stomaların açılıp kapanmasının dış ortamdaki oransal nem tarafından kontrol edildiğini açıklamışlardır. Çalışmaya ilişkin bulgularda, günün sıcak saatlerinde havanın oransal neminin artmasıyla stomalar kapanmakta ve Ψ_L değeri tekrar negatif yönde azalmaya başlamaktadır. Oke (1987), toprakta yeterli su olduğunda stomaların tam olarak açık olduğu durumlarda, ağacın transpirasyon hızı, hava sıcaklığı, oransal nem, ağaç tacı tarafından absorbe edilen güneş ışınları (solar radyasyon) ve rüzgar hızı gibi atmosferik koşullar tarafından denetlendiğini açıklamıştır.

Antepfistığında yaprak su potansiyeli, bitkinin sulanabilme koşullarına bağlı olduğu kadar, iklimsel değişmelerden de etkilenmektedir. Ölçüm dönemlerinde hava sıcaklığında meydana gelen artış, yaprak su potansiyelini düşürmektedir.

4.5 Verim Sonuçları

Deneme konularından elde olunan kuru kırmızı kabuklu antepfistığı meyveleri, ağaç başına kg olarak hesaplanmış, Çizelge 4.12 ve Ek Çizelge 17' de, varyans analiz sonuçları ise, Ek Çizelge 19-22' de verilmiştir.

Varyans analizi sonuçlarına göre, farklı su ve azot düzeylerinin uygulandığı sulama konuları arasında, 1999 yılında, istatiktiksel olarak önemli farklılıklar bulunamamıştır. Anılan yılda konuların ortalama verimleri, 0.52 kg/ağaç olurken, geleneksel konusunda 0.45 kg/ağaç olarak bulunmuştur. Denemenin ilk yılı olan 1999' da, verim değerlerinin oldukça düşük olması, bir önceki yılın meyve yükünün fazla olmasından kaynaklanmaktadır. Konuların 1998 yılına ilişkin olarak alınan ön verim ortalamaları 9.82 kg/ağaç olarak bulunmuştur. Crane ve Iwakırı (1986)

antepfıstığı meyve yükünün fazla olduğu yıllarda, bir sonraki yılın meyvelerini oluşturacak çiçek tomurcuklarının yaklaşık % 99' unun döküldüğünü, bu nedenle ertesi yılda, ya hiç yada çok az meyve oluştuğunu belirtmişlerdir. Denemenin ilk yıl bulguları, araştırmacıların bulgularıyla uyumlu görülmektedir.

Deneme konularına ilişkin ortalama verimler, ilk yıla göre diğer yıllarda oldukça yüksektir. Deneme bitirildikten sonra uygulamaların etkisini izlemek amacıyla alınan 2003 yılı ortalama verimi, 19.90 kg/ağaç olarak elde edilmiştir. Benzer durum, Tekin (1992) tarafından da rapor edilmiştir.

Denemenin ikinci yılında, sulama suyu ve azot düzeyleri arasında, 0.05 düzeyinde önemli ayrımlar bulunmuştur. Su ve azot düzeylerinin konu ortalamaları üzerindeki etkilerini belirlemek için LSD testi yapılmış ve deneme konuları istatistiksel olarak üç grup oluşturmuşlardır (Çizelge 4.12).

Su ve azotun farklı uygulandığı konulara bakıldığında, 2000 yılında en yüksek verim, 14 gün sulama aralığı ve 15 mg/l azot uygulanan $14N_2$ konusundan 18.32 kg/ağaç, en düşük verim ise 5.2 kg/ağaç olarak 7 günlük sulama aralığının kullanıldığı ve 10 mg/l azot uygulanan $7N_1$ konusundan elde edilmiştir. En yüksek ortalama verim değeri ile en düşük ortalama verim değeri arasında % 71.6, geleneksel konu ile % 75.4' lük bir azalma olduğu görülmektedir $14N_2$ ve sulanmayan "geleneksel" konu, *t* testi ile karşılaştırılmışlardır. Bu amaçla 10 ağaç kullanılmıştır. İki konu arasında istatistik olarak 0.05 önem düzeyinde farklılık bulunması ($t=6.4$, $n=10$, $p \leq 0.05$), sulamanın antepfıstığı verimini "geleneksel" konuya kıyasla yaklaşık 4 kat; sulanan konu ortalamaları ise yaklaşık % 77.7 oranında artırdığını göstermektedir. Var yılı olan 2000' yılında konuların ortalama verimleri, bir önceki yıla oranla, yaklaşık 20 kat bir artış göstererek, 10.31 kg/ağaç olarak bulunmuştur. Geleneksel konu ile karşılaştırıldığında ortalama verimdeki artış oranı 2.29 kat olarak saptanmıştır (Çizelge 4.12).

Yok yılı olan 2001' de, su ve azot düzeyleri arasında 0.05 düzeyinde önemli ayrımlar bulunmuştur. Deneme konuları, su ve azot değerlerine göre LSD testi ile değerlendirilmiş ve istatistiksel olarak 3 grup oluşturmuşlardır.

Deneme konuları arasında en yüksek verim, ortalama 12.02 kg/ağaç değeri ile, 14 günlük sulama aralığında, azotlu gübrenin uygulanmadığı $14N_0$ konusundan,

en düşük verim değeri ise ortalama 4.64 kg/ağaç ile, 7 günlük sulama aralığında ve 20 mg/l azot uygulanan konudan elde edilmiştir. En yüksek ve en düşük ortalama verim değerleri arasında % 61.4 oranında ayrım bulunmuştur. Geleneksel konu ortalaması ile en yüksek verim düzeyine sahip $14N_0$ konuları, ortalamalar arasındaki ayrım düzeyini belirlemek amacıyla *t testi* ile karşılaştırılmışlardır. İki konu ortalamaları arasında istatistiksel anlamda önemli bir ilişki bulunamamıştır ($t=1.68$, $n=10$, $p \leq 0.05$).

Çizelge 4.12. Uygulama Yıllarında Alınan Verim Değerleri (kg/ağaç)[a]

Konu	Yıllar				Ort.
	1999	2000	2001	2002	
$7N_0$60	0.81	15.79 ab	7.00 ab	13.38	9.24
$7N_0$90	1.32	14.12 ab	6.91 ab	9.90	8.06
$7N_1$ 60	0.11	2.96 b	9.96 ab	4.21	4.31
$7N_1$ 90	0.12	7.44 b	8.34 ab	3.57	4.86
$7N_2$ 60	0.36	6.68 ab	10.37 ab	4.26	5.41
$7N_2$ 90	0.00	8.14 ab	9.00 ab	3.41	5.14
$7N_3$ 60	0.72	14.15 ab	4.69 b	6.58	6.53
$7N_3$ 90	1.11	16.02 ab	4.58 b	8.56	7.56
$14N_0$60	0.04	3.86 ab	11.74 a	2.70	4.58
$14N_0$90	0.08	11.00 ab	12.29 a	3.78	6.79
$14N_1$ 60	0.40	9.55 ab	8.57 ab	6.82	6.33
$14N_1$ 90	0.00	5.99 ab	7.35 ab	2.92	4.07
$14N_2$ 60	1.66	19.95 a	5.73 ab	6.93	8.57
$14N_2$ 90	1.03	16.69 a	5.45 ab	6.93	7.52
$14N_3$ 60	0.08	4.82 ab	10.56 ab	2.66	4.53
$14N_3$ 90	0.54	7.80 ab	12.85 ab	3.50	6.17
Ortalama	0.52	0.31	8.46	5.63	6.23
**LSD_1[b]**	**ns**	**12.16**	**7.06**	**ns**	
**LSD_2[c]**	**ns**	**11.07**	**6.42**	**ns**	
Geleneksel[d]	0.45	4.51 b	9.10	2.80	4.21
$14N_2$		18.32 a			
$t_{0.05} \geq 2.26$		t_h=6.4			
$14N_0$			12.02		
$t_{0.05} \geq 2.26$			t_h=1.68		

[a] İki yinelemenin ortalamasıdır.
[b] Aynı azot düzeyinde, aynı veya farklı sulama aralıkları için.
[c] Aynı veya farklı azot düzeyinde, farklı sulama aralıkları için.
[d] Eş yapma tekniği ile karşılaştırma yapılmıştır.

Ortalama verimin en yüksek olduğu 14No konusu ile geleneksel arasında verimde meydana gelen ayrım oranı % 24.3 olarak hesaplanmıştır (Ek Çizelge 18). Bu bulgular ışığında, konularda meydana gelen verim artışının azottan daha fazla, uygulanan sulama suyundan etkilendiği söylenebilir.

Sulama suyu ile azot arasında, 0.05 düzeyinde önemli ayrımların olduğu 2000 ve 2001 yıllarına ilişkin konuların çok yıllık ortalamasına bakıldığında, en yüksek ortalama verim %' si, 12.84 kg/ağaç değeri ile $14N_260$ konusundan elde edilmiştir. Aynı konu, 1999 ve 2000 yıllarında da en yüksek verim değerine sahiptir. En yüksek verim değerinin elde edildiği $14N_260$ konusu ile geleneksel konu arasında % 47 oranında verim azalması saptanmıştır (Ek Çizelge 18).

Verim yılı olan 2002' de sulama, azot ve sulama aralığı gibi diğer uygulanan faktörlerden daha etkili bulunmuştur. En yüksek verim, 13.38 kg/ağaç değeri ile $7N_060$, en düşük verim değeri ise 2.66 kg/ağaç ile $14N_360$ konularından elde edilmiştir. Yapılan istatistik analizde, konular arasında istatistiksel anlamda önemli bir ayrım bulunamamıştır. Konular arasında görülen ortalama verim farklılıkları tamamen rastlantısaldır. En yüksek verim, geleneksel konu veriminden yaklaşık 5 kat daha fazladır (Çizelge 4.12).

Denemenin yürütüldüğü dört yıl boyunca elde edilen yığışımlı verim değerleri, sulama ve azot düzeylerine göre değerlendirilmiştir (Şekil 4.17a ve b). İncelenen bütün konularda 1999 yılı ölçümlerine göre, yığışımlı verim artmıştır. Ancak, elde edilen eğrilerin eğimleri birbirinden farklı olduğundan değinilen artış, bütün konularda aynı oranda olmamıştır. Eğrilerin eğimlerindeki bu farklılıklara karşın sulama ve azot etmenlerinin yığışımlı verim üzerinde, yaklaşık aynı etkiyi gösterdiği saptanmıştır. Sulanan ve gübrelenen konulardaki verimde meydana gelen artış oranı, sulanmayan ve sadece azotlu gübreleme yapılan "Geleneksel" konusundan oldukça yüksektir.

Periyodisite yılı olan 1999' da konuların ortalama verimleri yaklaşık aynı düzeydedir. "Geleneksel" konusunda bu değer 0.45 kg/ağaç iken, sulanan konularda 0.52 kg/ağaç olarak bulunmuştur. Benzer olarak 2000-2001-2002 yıllarında sulanan konulardaki ortalama yığışımlı verim değerleri, sırasıyla 9.51 kg/ağaç 17.97 kg/ağaç ve 23.6 kg/ağaç olarak bulunmuştur.

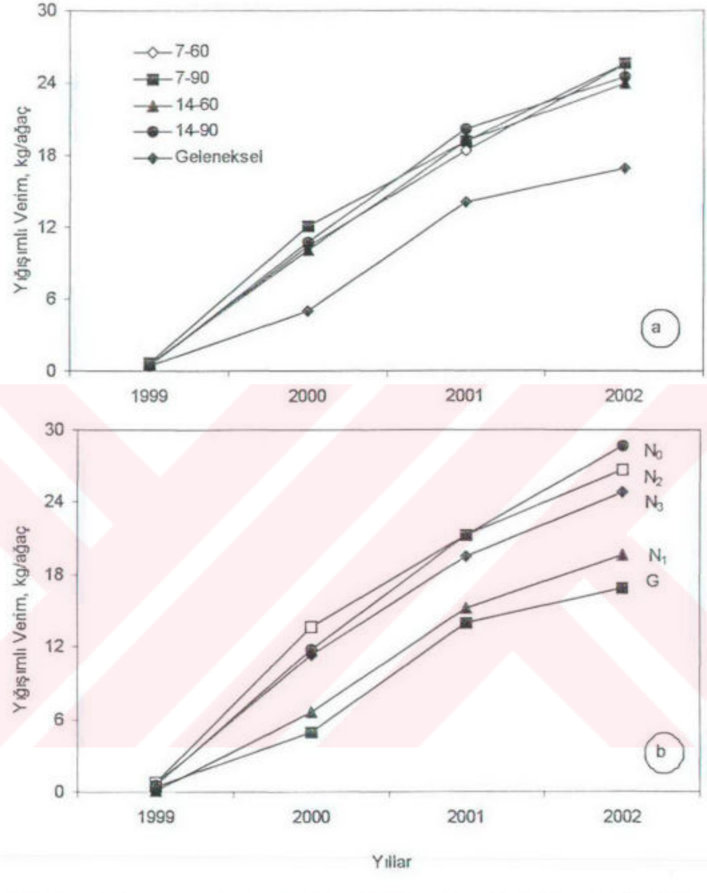

Şekil 4.17. Deneme yıllarında konulara ilişkin yığışımlı verim. Grafik, incelenen konularda, (a) farklı su (b) farklı azot düzeylerini göstermektedir.

1999 yılının periyodisite yılı olması ve denemenin başladığı ilk yıl olması nedeniyle, sulamanın etkisinin hemen görülemeyeceği bilindiğinden, verimde meydana gelen artış oranlarını belirlemek için yapılan analizde, "geleneksel" konusunun 2000 yılı değerlerine göre 2001 ve 2002 yılına ait sulanan konuların

ortalama verim değerleri karşılaştırılmıştır. Buna göre 2000, 2001 ve 2002 yıllarında, 2000 yılı geleneksel konusuna göre verimde meydana gelen artış oranı sırasıyla 1.9-3.6 ve 4.8 olarak bulunmuştur (Şekil 4.17a). Benzer olarak, azotlu konular için aynı analizler yapıldığında sulanan konulardan elde edilen artış oranları ile aynı değerler elde edilmiştir (Şekil 4.17 b).

Aydeniz (1990), antepfıstığında 20-40 g/m^2 azot ve fosfor uygulamasının, verimi en az iki kat artırdığını ve periyodisiteyi gerilettiğini belirtmiştir. Tekin (1992), antepfıstığında kuru koşullarda yaptığı gübreleme çalışmasında, 5.5 g/m^2 azot uygulamasının, verimde % 50'ye varan artışlar sağladığını, çıtlama oranı ve iç meyve randımanı üzerinde olumlu etki yaptığını belirtirken, Tekin ve Güzel (1993), gübrelemenin meyve gözü dökülme oranını azalttığını ancak tamamen durduramadığını açıklamışlardır. Sunulan çalışmada, birim alana, kuru koşullardakinden daha fazla azot uygulanmıştır. Ancak araştırmacıların belirttiği gibi, verimdeki artış hızı beklenildiği kadar olmamıştır.

4.5.1 Sulama Suyu - Verim İlişkisi

Deneme yılları, verim ve sulama suyu ilişkisinin kestiriminde, var ve yok yılları olarak iki gurupta incelenmiştir.

Verim değerleri, her iki dönemde de farklı azot derişimlerindeki konuların, yok yılları olan 1999 ve 2001' de sulama suyu ile verim arasında herhangi bir ilişki bulunamamıştır. Var yıllarında Şekil 4.18'de görüldüğü gibi, verim ile sulama suyu arasında 0.01 düzeyinde istatistiksel olarak önemli ikinci dereceden bir ilişki bulunmuştur (R^2= 0.7642, n=8). Sulama suyunun artmasıyla, verimde de önemli düzeyde artışlar elde edilmiştir. İlişki denkleminden, 13.00 kg/ağaç düzeyinde verim alınabilmesi için 933 mm sulama suyu uygulanması gerektiği söylenebilir.

Bilgel ve ark. (1999) Harran Ovası koşullarında yaptıkları çalışmada, sulama suyu ile verim değerleri arasında istatistiksel anlamda bir ilişki bulamamışlardır. Denemeye ilişkin bulgularla, değinilen araştırmacıların bulguları arasında görülen uyumsuzluğun, kullanılan sulama programları ve yöntemlerindeki farklılıktan kaynaklandığı düşünülebilir. Buna karşı, Kanber ve ark. (1993), sulamanın

sulanmayan antepfistığı ağaçlarına göre sadece verimi artırmakla kalmayıp ($P \leq 0.05$), aynı zamanda düşük verim yıllarında periyodisiteyi de önleyebildiğini belirtmişlerdir. Ancak, sulamanın verim üzerindeki etkisi olağan verim yılı ile periyodisite yıllarında farklılıklar göstermiştir.

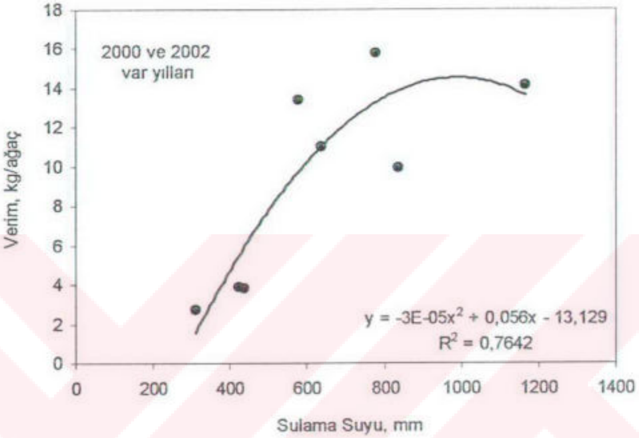

Şekil 4.18. Antepfistığında sulama suyu verim ilişkisi (2000 ve 2002)

Goldhamer ve ark. (1985), antepfistığında kullanılan su miktarı arttıkça, buna paralel olarak verimin de arttığını açıklamışlardır. Sunulan çalışmaya ilişkin bulgular, araştırmacıların belirttiği sonuçlarla uyumlu gözükmektedir.

4.5.2 Azot - Verim İlişkisi

Verim ve azot miktarı arasındaki ilişkinin elde edilmesinde, fırın kuru antepfistığı verim değerlerinin, yok ve var yıllarında yığışımlı değerleri ile dönem içerisinde konulara verilen toplam azot miktarlarının ortalama değerleri kullanılmıştır. Değerlendirmeler, verimin var ve yok yıllarına göre ayrı ayrı yapılmıştır. Şekil 4.19' da görüldüğü gibi, azot miktarları ile antepfistığı verimi arasında, yok yıllarında, istatistiksel anlamda önemli bir ilişki bulunamamıştır

(R^2=0.1653, n=17). Bu bulgulara göre, yok yıllarında antepfistığından, ağaç başına 10.58 kğ maksimum verimin elde edilmesi için, 5.74 g/m^2 azotun, sulama suyu ile birlikte uygulanması gerektiği söylenebilir. Denemeye ilişkin bu bulguların, Tekin (1992) ile uyumlu olduğu görülmektedir.

Var yılları ile ilgili verim değerleri ile uygulanan azot miktarları arasında anlamlı bir ilişki bulunamamıştır.

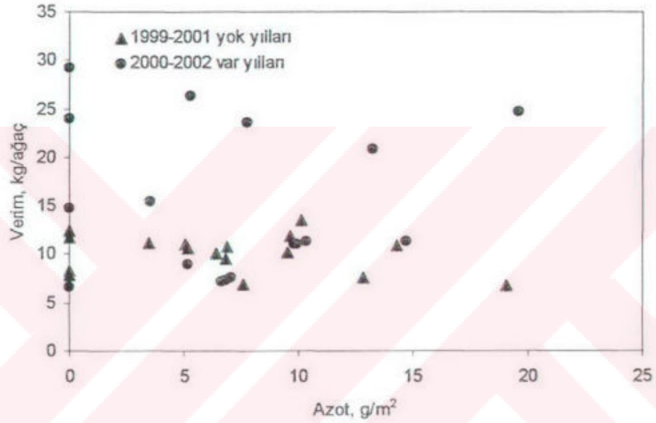

Şekil 4.19. Antepfistığında var ve yok yıllarında azot- verim ilişkisi (1999-2002)

Rosecrance ve ark. (1995), antepfistığında azotun, öncelikle verim ve periyodisite yıllarında, meyve içi doldurma döneminde alındığını belirtmişlerdir. Var yıllarında antepfistığı ağacının azot ve potasyum alımının, meyvesiz yıla oranla, % 35 ve % 112 oranında arttığını açıklamışlardır. Weinbaum ve Rosecrance (1998), antepfistığı ağacının meyveli yılda, periyodisite yılına oranla daha fazla azota gereksinim duyduğunu, periyodisite yılında ağacın vegetatif gelişimi daha fazla olsa bile, bu gereksinimin artmayacağını açıklamışlardır. Periyodisite yılında uygulanacak azot miktarının, verim yılına göre 1/3 oranında olması gerektiğini belirtmişlerdir. Araştırmacılar, ağaca uygulanacak gerçek azot miktarının, bir önceki yılın meyve yüküne, ağacın dikim aralıklarına, azot düzeylerine ve kullanılan sulama sistemine

bağlı olarak değişebileceğini rapor etmişlerdir. Sunulan araştırma bulgularına göre, verim yıllarında verim ile azot miktarları arasında herhangi bir ilişkinin bulunamaması, kullanılan azot miktarının azlığına veya kullanılan azotun formuna ve yüksek buharlaşmaya bağlanabilir.

4.6 Kalite Analizleri

Deneme konularından elde edilen antepfistığı meyvelerinin, kalite özelliklerinden, çıtlama oranı, 100 dane ağırlığı ve iç meyve randımanı değerlerine ilişkin sonuçlar Çizelge 4.13 ve Ek Çizelge 23' de verilmiştir. Bunlar, varyans analizi ile değerlendirilmiş ve elde olunan sonuçlar Ek Çizelge 24-26'da gösterilmiştir.

Antepfistığında en önemli meyve kalite özellikleri olarak kabul edilen çıtlama oranı, 100 dane ağırlığı ve iç meyve randımanı değerleri, denemenin 2. 3. ve 4. yıllarında incelenmiş, ilk yılda antepfistığı verimi oldukça düşük olduğundan bu ölçütlere bakılmamıştır.

Elde edilen sonuçlara göre, çıtlama oranı, 100 dane ağırlığı ve iç meyve randımanı değerleri arasında, istatistiksel anlamda önemli ayrımlar bulunamamıştır. Ancak, meyve kalite özellikleri, deneme yıllarına göre, t-testi yapılarak karşılaştırılmış, yıllar arasında önemli ayrımlar bulunmuştur. Buna göre, deneme konularının çıtlama oranlarına etkileri, 2000 ve 2001 yıllarında farksız bulunurken, verim yılları olan 2000 ve 2002 yılları arasında 0.01 düzeyinde önemli ayrımlar bulunmuştur ($t= 5.632$, $n=17$, $P \leq 0.01$). Benzer şekilde, 2001 ve 2002 yılları arasında da 0.01 düzeyinde önemli ayrımların olduğu belirlenmiştir ($t= 7.721$, $n=17$, $P \leq 0.01$).

100 dane ağırlıkları, aynı yöntemle karşılaştırılmış ve her üç deneme yılında da farklar önemli bulunmuştur. Elde edilen sonuçlara göre, 2000-2001 yılları kendi aralarında farklılıklar göstermiş, yıllar arasında 0.01 düzeyinde önemli ayrımların olduğu belirlenmiştir ($t=3.707$, $n=17$, $P \leq 0.01$).Verim yılları olan 2000 ve 2002, birbirinden 0.01 düzeyinde farklıdır ($t= -8.864$, $n=17$, $P \leq 0.01$). Aynı şekilde, 2001 ve 2002 yılları da 0.01 düzeyinde önemli ayrımlar göstermiştir ($t= -3.373$, $n=17$, $P \leq 0.01$).

Çizelge 4.13. Antepfistığında Meyve Kalite Özelliklerine İlişkin Sonuçlar

KONULAR	ÇITLAMA ORANI (%)			ORT.	100 DANE AĞR. (gr)			ORT.	İÇ MEY.RAND. (%)			ORT.
	2000	2001	2002		2000	2001	2002		2000	2001	2002	
$7N_0 60$	57.5	67.3	61.3	62.1	99.3	118.5	114.7	110.8	40.5	41.9	37.84	40.1
$7N_0 90$	64.3	75.0	48.7	62.7	99.7	113.1	120.2	111.0	42.6	41.5	37.34	40.5
$7N_1$ 60	70.2	43.2	52.0	55.1	106.2	127.3	115.1	116.2	40.7	42.1	37.82	40.2
$7N_1$ 90	76.7	86.3	56.5	73.2	107.5	103.6	117.9	109.7	42.0	41.8	37.75	40.5
$7N_2$ 60	60.7	70.2	51.0	60.6	104.3	111.4	121.3	112.3	42.3	41.5	37.99	40.6
$7N_2$ 90	52.0	61.7	35.3	49.7	98.5	123.0	121.9	114.5	42.7	41.3	37.17	40.4
$7N_3$ 60	79.5	71.3	40.7	63.8	108.0	112.2	118.1	112.8	43.0	42.1	36.24	40.4
$7N_3$ 90	69.0	81.0	45.2	65.1	109.5	121.9	117.8	116.4	43.3	42.3	37.70	41.1
14No60	53.2	76.0	54.0	61.1	103.7	110.4	115.7	109.9	41.0	40.6	37.32	39.6
14No90	79.7	76.2	44.8	66.9	111.7	105.9	122.9	113.5	41.5	40.7	37.74	40.0
$14N_1$ 60	57.7	78.2	52.7	62.8	113.8	109.0	119.0	114.0	39.3	41.3	38.36	39.6
$14N_1$ 90	71.8	75.8	57.3	68.3	108.7	114.9	118.4	114.0	42.4	41.1	37.73	40.4
$14N_2$ 60	81.2	81.2	46.3	69.6	111.2	109.8	128.6	116.5	41.0	41.6	36.92	39.8
$14N_2$ 90	72.2	76.0	43.2	63.8	105.0	107.0	121.4	111.1	42.5	41.3	37.44	40.4
$14N_3$ 60	50.5	75.5	52.5	59.5	108.3	116.0	122.3	115.5	41.2	42.0	36.88	40.0
$14N_3$ 90	57.7	70.7	42.2	56.8	110.0	113.7	125.3	116.3	42.3	41.2	38.16	40.5
Geleneksel	71.8	59.7	55.0	62.2	101.3	109.2	111.9	107.5	41.7	40.3	39.03	40.4
**LSD**	**ns**	**ns**	**ns**		**ns**	**ns**	**ns**		**ns**	**ns**	**ns**	
$t_{0.05} \geq 2.389$	t_h=1.686				t_h=3.707				t_h=1.120			
$t_{0.01} \geq 2.704$		t_h=7.721				t_h=3.373				t_h=18.731		
	$t_{h\text{-}2000\text{-}2002}$=5.632				$t_{h\text{-}2000\text{-}2002}$=8.864				$t_{h\text{-}2000\text{-}2002}$=13.998			

İç meyve randımanlarına ilişkin yapılan karşılaştırmada 2000 ve 2001 yılları arasında herhangi bir ilişki bulunamazken, verim yılları olan 2000 ve 2002 yılları arasında 0.01 düzeyinde önemli ayrımlar bulunmuştur ($t= 13.998$, $n=17$, $P \leq 0.01$). Benzer olarak 2001 ve 2002 yılları da 0.01 düzeyinde önemli ayrımlar göstermiştir ($t= 18.731$, $n=17$, $P \leq 0.01$).

Deneme konularına ilişkin ortalama verim değerleri ile geleneksel konu arasında meyve kalite özellikleri bakımından herhangi bir ayrım olup olmadığını belirlemek amacıyla varyans analizi yapılmış ve sonuçlar Ek Çizelge 27' de verilmiştir. Çizelgenin incelenmesinden görüleceği gibi, geleneksel konu ile diğer konular, çıtlama oranları bakımından karşılaştırmaların yapıldığı yıllarda, sadece verim yılı olan 2001' de 0.05 düzeyinde farklı bulunmuş, verim yılları olan 2000 ve

2002' de herhangi bir ayrım bulunamamıştır. 100 dane ağırlıklarına ilişkin geleneksel ve diğer konuların karşılaştırılmasında üç yıllık sonuçlar için herhangi bir farklılık bulunamazken, iç meyve randımanı bakımından, 2002 yılında geleneksel konu ile diğer konular arasında 0.05 düzeyinde önemli farklar bulunmuştur. Benzer sonuçların, Monastra ve ark. (1997) tarafından alındığı rapor edilmiştir.

Karaca ve Nizamoğlu (1988), yerli ve yabancı antepfıstığı çeşitlerinin kimi kalite özelliklerini belirlemek amacıyla yaptıkları çalışmada, iri daneli çeşitlerin genotipe bağlı olarak daha fazla doğal çıtlaklığa sahip olduklarını bildirmişlerdir. Araştırmacılar, doğal çıtlaklık üzerine iklim etmenlerinin yanı sıra, ağacın ürün yükünün etkili olduğunu, verim arttıkça doğal çıtlama oranının azaldığını belirtmişlerdir. Sunulan çalışmadan elde edilen sonuçlar, Karaca ve Nizamoğlu (1988) ile karşıttır. Buna göre, deneme yıllarında konuların ağaç başına ortalama verimleri, yıllara göre sırasıyla 9.97 kg/ağaç, 8.50 kg/ağaç ve 5.46 kg/ağaç olarak azalan bir eğilim gösterirken, aynı yıllarda çıtlama oranlarının, verime göre artmadığı, yıllara göre % 66- %72 ve % 49 olarak değişim gösterdiği bulunmuştur. Bu çalışmada, meyve yükünün azalmasıyla çıtlama oranı artmamıştır. Açıklanan sonuçlar, deneme materyali olarak kullanılan *Uzun* çeşidinin genotipine bağlanabileceği gibi aynı zamanda yetiştirme tekniklerindeki ayrımlara, sulama ve gübrelemeye bağlanabilir. Denemeye ilişkin bu bulgular, Kanber ve ark. (1990) ile uyumlu görülmektedir. Goldhamer ve ark. (1985), antepfıstığında meyvenin çıtlama oranının yalnızca sulamaya göre değil, ağacın meyve yüküne göre de değişme gösterdiğini belirtmişlerdir. Uygulanan sulama suyuna bağlı olarak, çıtlama oranının arttığını belirten araştırmacıların bulgularıyla, denemeye ilişkin bulgular uyumlu görülmemektedir. Aradaki uyumsuzluğun, kullanılan materyalin çeşit özelliğinden kaynaklandığı düşünülebilir. Ferguson ve ark. (1995), çıtlama oranının azlığının ve boş meyve oluşumunun, ağacın toplam meyve yükünden daha çok, antepfıstığı ağacının periyodisite ve verim yıllarındaki durumları ile ilişkili olduğunu yazmışlardır. Ağacın karbonhidrat depolama kapasitesinin, boş ve çıtlamamış meyve oranını, toplam meyve yükünden daha fazla etkilediğini açıklamışlardır. Ülkemizde en çok yetiştiriciliği yapılan *Uzun* ve *Kırmızı* çeşidi olduğunu belirten Arpacı ve ark. (1997), bu çeşitlerin periyodisiteye eğiliminin daha fazla, çıtlama oranı ve meyve

iriliğinin, diğer çeşitlere göre, daha düşük olduğunu bildirmişlerdir. Çıtlama oranlarının düşük çıkması, araştırmacıların bu bulguları ile uyumludur. Crane ve Iwakırı (1986), anaçların antepfıstığında verim ve meyve kalite özellikleri üzerine etkilerini belirlemek amacıyla yaptıkları çalışmada, yıllık ortalama verim ile antepfıstığı ağacının gövde kesit alanı arasında pozitif bir ilişki olduğunu belirterek, çıtlama oranı ve boş meyve oluşumunun, kullanılan anacın bir fonksiyonu olduğunu kaydetmişlerdir.

İç meyve randımanları, deneme yıllarında farklı değişim göstermiş, en düşük değer, 2002 yılında, % 37.6; denemenin 2000 ve 2001 yıllarında bu değerler sırasıyla % 41.77 ve % 41.44 olarak bulunmuştur. Karaca ve Nizamoğlu (1988), antepfıstığının % iç meyve randımanı yönünden kullanılan çeşitlere göre farklılıklar gösterebileceğini belirtmiş; ancak, bu farklılıkların nedenleri konusunda herhangi bir açıklama yapmamışlardır. Deneme sonuçlarına göre, sulama suyu ile iç meyve randımanı arasında herhangi bir bağdaşım bulunamamıştır. Benzer sonuçlara ulaşan Monastra ve ark. (1997), sulamanın yaş ve kuru iç meyve miktarını artırdığını, buna karşılık iç meyve randımanı üzerinde etkili olmadığını saptamışlardır. Araştırmacılar, sulamanın antepfıstığında meyvelerin ortalama ağırlığı üzerinde etkili olmadığını, ancak iç meyve büyümesi yoluyla değil, ağaçtaki meyve miktarı üzerinde artışa neden olduğunu açıklamışlardır. Deneme sonuçlarına göre, 100 dane ağırlıkları deneme yıllarında artan bir eğilim göstermiştir. Bu durumun, sulamanın etkisi sonucu ortaya çıktığı düşünülebilir.

4.7 Su Kullanma Randımanları

Denemede seçilen dört sulama konusuna ilişkin toplam su kullanma randımanları (TWUE) ile sulama suyu kullanma oranı (IR_c) ve sulama suyu kullanma randımanları (IWUE) hesaplanmıştır. Deneme konularına ilişkin elde edilen sonuçlar Şekil 4.20-22'de gösterilmiştir.

Şekil 4.20'in incelenmesinden görüleceği gibi, toplam sulama suyu kullanma randımanları, sulama konularına ve yıllara göre, değişmeler göstermiştir. Denemenin ilk yılında TWUE değerleri, konularına bakılmaksızın düşük bulunmuştur ($S_{\bar{x}}$ =

0.394; $\overline{X}$= 0.749 kg/m^3-ağaç). En yüksek TWUE değeri, 2001 yılında 18.46 kg/m^3-ağaç ile 14N$_3$60 konusunda hesaplanırken, en düşük TWUE değeri, yine aynı konuda 0.17 kg/m^3-ağaç 1999 yılında bulunmuştur.

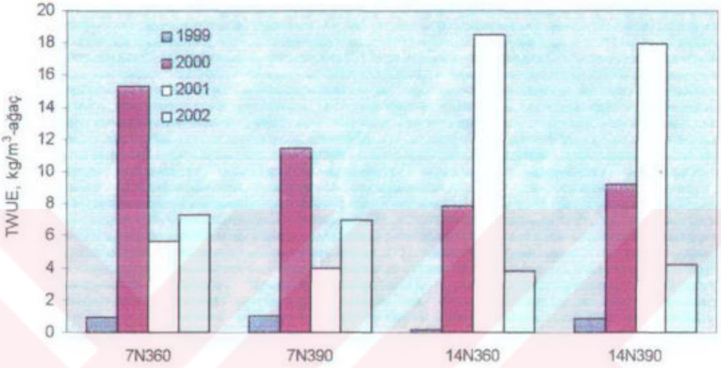

Şekil 4.20. Kimi deneme konularına ilişkin toplam su kullanma randımanları

Periyodisite yıllarında (1999 ile 2001) TWUE, ilk yıl değerleri daha düşük ($S_{\overline{x}}$ = 0.394; $\overline{X}$= 0.749 kg/m^3-ağaç), 2001 yılında ise daha yüksek bulunmuştur ($S_{\overline{x}}$ =7.768; $\overline{X}$ = 11.505 kg/m^3-ağaç). Deneme yılları arasında istatistiksel olarak 0.05 düzeyinde farkların olduğu saptanmıştır (t=2.766, n=4, $P \leq 0.05$). Konular arasında yapılan istatistiksel değerlendirmede, ortalamalar arasındaki farkların önemli olmadığı bulunmuştur ($S_{\overline{x}}$ = 7.768; $\overline{X}$ = 11.505 kg/m^3-ağaç).

Verim yıllarında (2000 ve 2002) TWUE değerleri, yıllara göre farklılıklar göstermiştir. En yüksek TWUE değeri 7N$_3$60 konusunda (15.3 kg/m^3-ağaç), en düşük TWUE ise 14N$_3$60 konusunda, 2002 yılında elde edilmiştir (3.8 kg/m^3-ağaç). Verim yılları arasında yapılan istatistiksel analizde, ortalamalar arasındaki fark 0.05 düzeyinde önemli bulunurken (t=2.87, n=4, $P \leq 0.05$), konular arasında yapılan karşılaştırmada, her iki yılda da fark bulunmamıştır ($S_{\overline{x}\,2000}$ = 3.264, $\overline{X}_{2000}$ = 10.941 kg/m^3-ağaç; $S_{\overline{x}2002}$ = 1.827, $\overline{X}_{2002}$ = 5.572 kg/m^3-ağaç).

Deneme yıllarında konuların toplam sulama suyu kullanma randımanları, yıllara göre farklı değerler almıştır. Periyodisite yılı olan 1999 da, TWUE değerlerinin çok düşük hesaplanması, bu yıla ilişkin verim değerlerinin ve dolayısıyla meyve yükünün düşüklüğüne bağlanabilir.

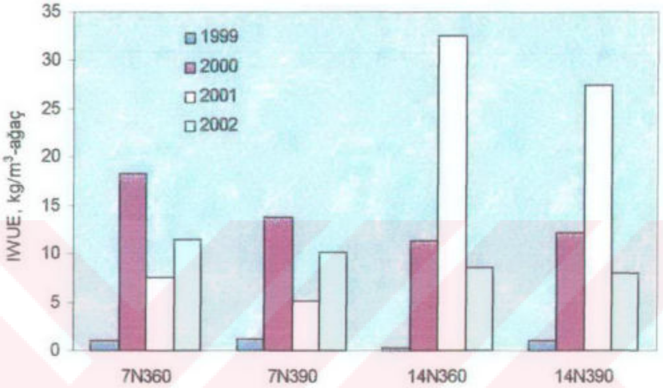

Şekil 4.21. Kimi konularda sulama suyu kullanma randımanlarının deneme yıllarında karşılaştırılması

Konuların sulama suyu kullanma randımanları (IWUE), dört yıllık olarak karşılaştırıldığında, en yüksek IWUE değeri, 2001 yılında $14N_{3}60$ konusunda (32.59 kg/m^{3}-ağaç) bulunurken, en düşük IWUE değeri yine aynı konuda 1999 yılında (0.22 kg/m^{3}-ağaç) elde edilmiştir (Şekil 4.21).

Periyodisite yılları olan 1999 ve 2001, istatistiksel olarak 0.05 düzeyinde farklı (t=2.495, n=4, $P \leq 0.05$), konu ortalamaları kendi aralarında her iki yılda da farksız bulunmuştur ($S_{\bar{x}1999}$ = 0.423, $\bar{X}_{1999}$ = 0.85 kg/m^{3}-ağaç; $S_{\bar{x}2001}$ = 13.893, $\bar{X}_{2001}$ = 18.191 kg/m^{3}-ağaç).Verim yıllarında (2000 ve 2002) en yüksek IWUE değeri, $7N_{3}60$ konusunda 18.23 kg/m^{3}-ağaç, en düşük IWUE değeri ise, $14N_{3}90$ konusunda 8.01 kg/m^{3}-ağaç olarak hesaplanmıştır. Yıllar arasında istatistiksel olarak 0.05 düzeyinde önemli farkların olduğu saptanmıştır (t= 2.528, n= 4, $P \leq 0.05$). Konular arasında her iki yılda farklılık bulunamamıştır ($S_{\bar{x}2000}$= 3.064, $\bar{X}_{2000}$ =

13.891 kg/m^3-ağaç; $S_{\bar{x}2002}$ = 1.556, $\overline{X}_{002}$= 9.548 kg/m^3-ağaç). Sulama suyu kullanma randımanları konular bakımından değerlendirildiğinde, 14 günlük sulama aralığının kullanıldığı konularda 2001 yılı değerleri daha yüksek bulunmuştur.

Sulama suyu kullanma randımanları bakımından, konular arasında farklılıklar bulunmamasına karşı, yılların birbirinden farklı olması, antepfıstığının periyodisite göstermesine ve verim değerlerinin yıldan yıla değişmesine, ağacın meyve yükünün fazlalığına bağlanabilir. Verimin fazla olduğu yıllarda, bitkilerin sulama suyu kullanma randımanları da yüksek bulunmuştur. Konuların sulama düzeyleri, sulama suyu kullanma randımanları üzerinde çok fazla etkili olmamıştır. Bilgel ve ark. (1999) tarafından Harran ovası koşullarında yürütülen çalışmalarda, en yüksek sulama suyu kullanım randımanı değerlerini, yine en uzun sulama aralığında sulanan (45 gün) konudan (11.9 kg/ha/mm) elde etmişlerdir.

Sulama suyunun evapotranspirasyon içindeki kullanım oranı olan IR_c değerleri (Şekil 4.22), deneme süresi boyunca oldukça yüksektir. Deneme yıllarında en yüksek IR_c değeri, 1999 yılında $7N_390$ konusunda 0.93; en küçük değer ise, 2002 yılında $14N_360$ konusunda 0.44 olarak bulunmuştur. IR_c değerleri arasında konulara bakılmaksızın, en yüksek değer ile en düşük değer arasında yaklaşık % 53' lük bir azalma söz konusudur.

IR_c değerlerinin periyodisite yıllarına ilişkin olarak karşılaştırılması için yapılan t testinde, yıllar arasında 0.05 düzeyinde önemli farklılıklar bulunmuştur ($t=2.943$, $n=4$ $P \leq 0.05$). Periyodisite yıllarında konular arasında istatistiksel olarak fark bulunamamıştır ($S_{\bar{x}1999}$ = 0.063, $\overline{X}_{1999}$ = 0.86 kg/m^3-ağaç; $S_{\bar{x}2001}$ = 0.098, $\overline{X}_{2001}$ = 0.687 kg/m^3-ağaç). Verim yıllarına ilişkin yapılan değerlendirmede ise yıllar arasında 0.05 düzeyinde önemli farklar olduğu belirlenmiştir ($t=3.166$, $n=4$ $P \leq 0.05$). Verim yıllarında konular arasında istatistiksel olarak farklılık saptanamamıştır ($S_{\bar{x}2000}$ = 0.07, $\overline{X}_{2000}$ = 0.778 kg/m^3-ağaç; $S_{\bar{x}2002}$ = 0.109, $\overline{X}_{2002}$= 0.573 kg/m^3-ağaç). Sulama suyu kullanma oranının, su kullanma randımanlarına benzer biçimde, yıldan yıla farklılıklar göstermesine karşın, konular arasında farklılıkların bulunamaması, Kanber ve ark. (1996b)'nın belirttiği gibi, bitki su

tüketiminin konulara göre uygulanan sulama suyu miktarına bağlı olarak değişmesinden kaynaklanmış olabilir.

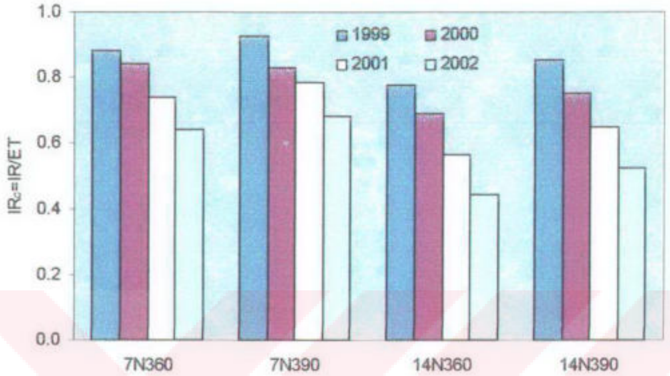

Şekil 4.22. Konulara ilişkin sulama suyu kullanma oranlarının deneme yıllarında değişmesi

4.8 Verim Fonksiyonları (K_y)

Oransal evapotranspirasyon açığı ile oransal verim azalışları arasındaki ilişki incelenmiştir. Bu amaçla, önce tüm yıllar için verim ile su tüketimi arasındaki ilişki, elde edilmiş ve buradan Köksal ve ark. (2001) tarafından verilen yaklaşımlar kullanılarak, antepfistığına ilişkin verim tepki etmeni (ky) değeri kestirilmiştir.

Yapılan bu işlemler sonucunda,

$$(1\text{-}Ya / Ym) = 0.6267\ (1\text{-}ETa / ETm) + 0.2105; \quad R = 0.66$$

şeklinde belirlenen denklem elde edilmiştir. Buna göre durumda, antepfistığının tüm yetişme dönemi için verim tepki etmeninin ky = 0.95 olduğu söylenebilir (Şekil 4.23).

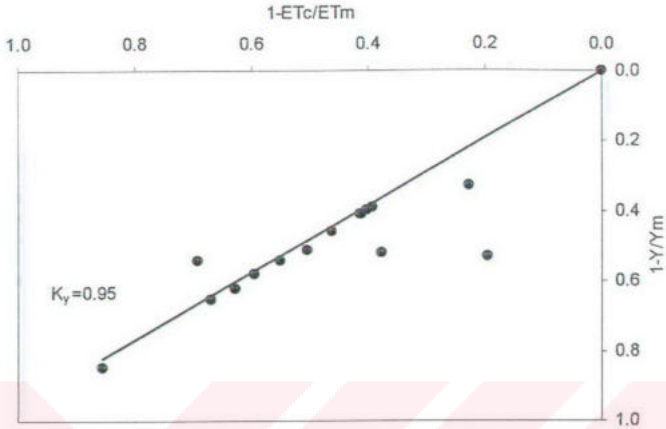

Şekil 4.23. Antepfıstığı için oransal su tüketimi azalışı oransal verim eksilişi arasındaki ilişkiler

Yok yıllarında su ve azot etkisiyle ürün alınabildiğinden dolayı, anılan dönemlere ilişkin verim tepki etmeni hesaplanmış ve Şekil 4.24' de gösterilmiştir. Ayrıca, değinilen işlem var yılları için yinelenmiştir. Konu edinilen şekilde görüldüğü gibi, yok yılları için K_y=1.09 ve var yılları için K_y=0.80 bulunmuştur. Buradan, antepfıstığının verim ve periyodisite yıllarında su eksikliğine karşı farklı duyarlılık gösterdiği söylenebilir. Ulaşılan sonuçlara göre, sulama suyunun eksik olduğu koşullarda, bir birim su eksikliğine karşı, en fazla verim azalışı, periyodisite yıllarında (1.09) beklenilebilir. Sonuç olarak, antepfıstığı periyodisite yıllarında verim yıllarına göre, suya karşı daha duyarlı iken; verim yıllarında su eksikliğinden ileri gelen gerilime karşı daha dayanıklı olduğu söylenebilir.

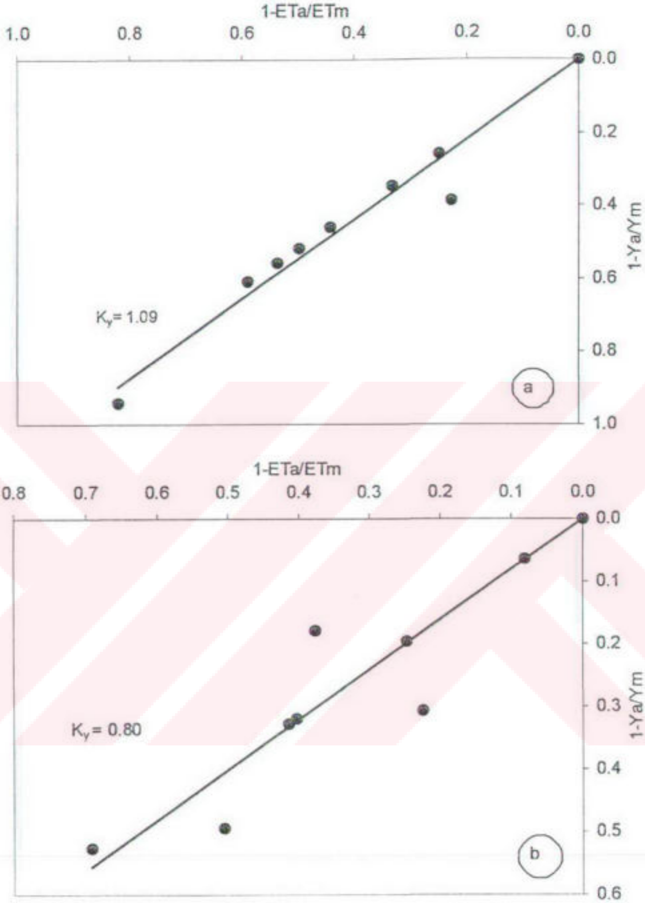

Şekil 4.24. Antepfıstığında periyodisite yıllarına (a) ve verim yıllarına ilişkin (b) verim tepki etmeni (K_y)

5. SONUÇLAR VE ÖNERİLER

5.1. Sonuçlar

Çalışma, Antepfıstığı Araştırma Enstitüsüne ait Havaalanı İşletmesi'nde 1999-2002 yılları arasında yürütülmüştür. Bu çalışmada, farklı su ve azot düzeyleri kullanılarak verim ve periyodisite ilişkisi irdelenmeye çalışılmış; bunun yanında fertigasyon tekniği kullanılarak uygun gübreleme programının elde edilmesi ve kullanıma sunulması amaçlanmıştır.

Deneme materyali olarak, *P. Vera L.* anacı üzerine aşılı *uzun* çeşidi kullanılmıştır. İki farklı sulama aralığı (7 ve 14 gün), dört farklı azot düzeyi (N_o, N_1, N_2, N_3) ve iki bitki-pan katsayısı (K_{cp1}= 0.60 ve K_{cp2}= 0.90) ele alınmıştır. Ng olarak simgelenen geleneksel gübreleme konusunda gübreler, Şubat ayı başında saf madde olarak ağaç başına 500 g N, 600 g P_2O_5, 400 g K_2O ağaçların taç izdüşümleri altına açılan bantlara verilmiştir. Sulama konularında azot miktarları N_o= 0 mg/l, N_1=10 mg/l, N_2= 15 mg/l ve N_3= 20 mg/l olarak sulama suyu ile birlikte uygulanmıştır. Fosfor ve potasyum gübreleri 10 ve 15 mg/l olarak 14 gün aralıklarla konulara eşit miktarlarda uygulanmıştır. Sulama düzeyleri deneme alanı içerisine yerleştirilen Class A Pan' dan oluşan yığışımlı evaporasyon değerlerinden yararlanılarak hesaplanmıştır.

Çalışmanın yürütüldüğü yıllarda sulama uygulamaları, iklimdeki değişmeler nedeniyle farklı tarihlerde başlatılmıştır. Bu nedenle, yıllara göre elde edilen yığışımlı evaporasyon miktarları 924 - 1294 mm olarak farklı düzeylerde bulunmuştur. Deneme yıllarında 7 ve 14 günlük sulama aralıklarında konular 18 ve 8 kez sulanmıştır. Konulara, kullanılan bitki-pan katsayılarına bağlı olarak farklı miktarlarda sulama suyu uygulanmıştır. Deneme yıllarında en yüksek ve en düşük su uygulama miktarı, 7 günlük sulama aralığının kullanıldığı konularda 837 - 1164 mm olarak hesaplanırken, 14 günlük sulama aralığında 0.60 katsayısının kullanıldığı konularda, 311 – 425 mm olarak bulunmuştur. Benzer olarak, damla sulama sisteminin kullanılmasıyla, 7-90 konusuna göre, 14-60 konusunda, deneme yılları boyunca ortalama en yüksek su artırımı, % 63.5, en düşük ortalama su artırımı ise, 7-90 konusuna göre, 7-60 konusunda % 32.4 olarak hesaplanmıştır.

Araştırmada bitki su tüketimleri, deneme konularında yıllara göre farklılıklar göstermiştir. En yüksek sulama suyunun uygulandığı $7N_390$ konusu, en düşük sulama suyunun uygulandığı $14N_360$ konusunda farklılık daha belirgindir. Buna göre, sulanan konularda en yüksek ve en düşük bitki su tüketimi deneme yılları boyunca 1228 mm ile 467.6 mm arasında değişmiştir. Genel olarak sulanan konu ortalaması 854.5 mm olarak hesaplanmıştır. Sulama uygulaması yapılmayan geleneksel konuda bitki su tüketimi, yıllık yağış miktarları ve toprak suyundaki değişmeye bağlı olarak 91.5 mm ile 376 mm arasında değişmiştir. $7N_390$ konusuna göre, $7N_360$ konusu ile $14N_360$ konularında, deneme yılları boyunca meydana gelen ortalama bitki su tüketimi azalışı, % 26.6 ile % 56.6 arasında değişmiştir.

Deneme yıllarına ilişkin ortalama en yüksek aylık su tüketimi değerleri, $7N_390$ konusunda 290 mm ile Temmuz ayında elde edilmiştir. Bu ayda gerçekleşen çim kıyas bitki su tüketimi (ET_o) ise 166 mm olarak bulunmuştur.

Denemede tam sulanan $7N_390$ konusunda, aylık bitki-pan katsayılarının dört yıllık ortalama değerleri hesaplanmıştır. Maksimum K_c değeri, temmuz ayında 1.51 olarak bulunurken, kasım ayında bu değer 0.39' a düşmüştür.

Denemede tam sulanan $7N_390$ konusu ile geleneksel konularında bir ağaç için ayrılan toplam alandan ve kök bölgesindeki her bir katmandan tüketim yoluyla oluşan su kayıpları ölçülmüştür. Bitki su tüketimi, genel olarak ağacın tacına doğru azalmış, bir ağaca ayrılan alanın köşe noktalarında daha yüksek bulunmuştur. Sulanan ve geleneksel konunun her ikisinden elde edilen sonuçlar, antepfıstığı köklerinin ağacın tacı altında etkin olmadığını göstermiştir. Evapotranspirasyon yoluyla topraktan oluşan su kayıpları, ET' nin yüksek olduğu farklı toprak katmanlarında, kök etkinliğinin de yüksek olduğunu kanıtlamıştır.

Antepfıstığı ağacı köklerinin, toprağın 30-90 cm' lik katmanlarında yoğunlaştığı anlaşılmıştır. Bitkinin 120 cm' lik kök bölgesinde, toplam kök yoğunluğunun % 0.5' lik kısmı ilk 30 cm' lik katmanda, 30-60, 60-90 ve 90-120 cm' lik katmanlarda ise sırasıyla % 40.8, 54.7 ve 4.1 bulunduğu saptanmıştır. Benzer olarak, bitkinin 120 cm' lik kök bölgesinden tüketim yoluyla oluşan su kayıplarının, $7N_390$ konusunda % 37.1' lik kısmının ilk 30 cm' den, % 45.4' ü ise kök yoğunluğunun fazla olduğu 30-90 cm' lik katmandan oluştuğu belirlenmiştir.

Denemeyi oluşturan konulardan seçilen tam sulanan ve en fazla azot verilen $7N_390$ konusu ile sulanmayan "Geleneksel" konularında yaprak su potansiyeli (Ψ_L) ölçümleri yapılmıştır. YSP ölçümleri, sulama dönemi başında, dönem ortasında ve sulama uygulamaları bitirildikten sonra olmak üzere üç dönemde yapılmıştır. Sulama dönemi başında yapılan ölçümlerde tam sulanan ve sulanmayan konularda Ψ_L değeri her iki konuda da, özellikle hava sıcaklığının arttığı öğle saatlerinde -3.7 MPa değerine kadar düşmüştür. Gündoğuşu ve gün batımı saatlerinde ise sıcaklığın düşmesi ile birlikte yeniden yükselerek (negatif yönde azalmaya başlamış) -1.6 MPa' a ulaşmıştır. Genel olarak, sulanan konularda Ψ_L değerleri sulama dönemi ortasında yaklaşık -2.5 MPa' kadar ulaşmakta, bu değerden sonra, stomalar kapanarak transpirasyonu önlemeye yönelmektedir. Sulanmayan geleneksel konusunda ise bu değer -3.68 MPa olarak bulunmuştur. Sulanan konularda, sulanmayan geleneksel konusuna göre sürekli bir azalma (negatif yönde artış) görülmüştür. Bu durum, sulanan konulardaki ağaçların köklerinin ve dolayısıyla yaprakların sulanmayan ağaçlara göre, sulanır ortama daha fazla koşullandığını ve uyum gösterdiğini kanıtlar niteliktedir.

Deneme yıllarında konulara ilişkin ortalama verim değerleri ile sulama suyu ve azot miktarları arasındaki ilişkiler elde edilmiştir. Ayrıca, konulardan derlenen ortalama verimler varyans analizleri ile karşılaştırılmışlardır. Denemenin ilk ve son yılında, ortalama verim değerleri arasında istatistiksel anlamda önemli bir ayrım bulunamazken, 2000 ve 2001 yıllarında sulama suyu-azot etkileşimi arasında 0.05 düzeyinde önemli farklılıkların olduğu anlaşılmıştır. En yüksek ortalama verim değerleri, 2000 yılında 14 günlük sulama aralığında ve 15 mg/l azot uygulanan $14N_2$ konusundan 18.32 kg/ağaç olarak, 2001 yılında en yüksek ortalama verim, aynı sulama aralığında ve azot uygulanmayan $14N_0$ konusundan 12.02 kg/ağaç olarak elde edilmiştir. Bu bulgular ışığında sulamanın verim üzerinde, azot uygulamasından daha etkili olduğu söylenebilir.

Antepfıstığının periyodisite eğilimi göstermesi nedeniyle deneme yılları, kendi aralarında verim ve periyodisite yılları olarak iki gurupta incelenmiştir. Var yıllarında, verim ile sulama suyu arasında 0.01 düzeyinde önemli olan, ikinci dereceden bir ilişki olduğu anlaşılmıştır. Anılan ilişkiler kullanılarak en yüksek

ürünün elde edilmesi için gerekli olan sulama suyu miktarı hesaplanmıştır. Buna göre, 933 mm sulama suyu uygulaması ile optimum verim düzeyinin yakalanabileceği anlaşılmıştır.

Aynı şekilde, verim ile azot miktarları arasında da periyodisite yıllarında ikinci dereceden ilişkiler elde edilmiştir. Ağaç başına maksimum verim alınabilmesi için, saf madde olarak 5.74 g/m^2 azotun sulama suyu ile birlikte uygulanması gerekmektedir.

Çalışmada, periyodisitenin temel nedeni olarak bilinen meyve gözü (karagöz) dökülme oranları ile verim değerleri arasındaki ilişki incelenmiş ve aralarında ikinci dereceden bağıntı olduğu belirlenmiştir. Meyve gözlerinin % 27 oranında dökülmelerinin verim üzerinde herhangi bir düşmeye neden olmadığı, bundan daha yüksek oranlarda gerçekleşen dökülme oranlarında ise, verim miktarında önemli azalmaların meydana geldiği belirlenmiştir.

Deneme yılları boyunca su ve azotun farklı düzeylerde uygulandığı konularda periyodisite indeksi (I) değerleri hesaplanmış ve genel olarak birbirine yakın değerler bulunmuştur. Su ve azot düzeylerindeki farklılıklar, periyodisite indeksi değerlerinde belirgin olarak görülmemiştir. En yüksek periyodisite indeksi değeri, 14$N_0$60 konusundan 0.766; en düşük değer ise, 7$N_0$90 konusunda 0.501, bütün deneme konularından ortalama olarak 0.604 olarak hesaplanmıştır. Geleneksel konudan hesaplanan periyodisite indeksi (0.627), ortalamadan daha yüksek bulunmuştur. Bu durum, deneme materyali olarak kullanılan Uzun çeşidinin periyodisiteye olan eğiliminin fazla olmasından kaynaklanmaktadır.

Deneme konularından elde edilen antepfıstığı meyveleri, kalite analizleri bakımından her yıl kendi içerisinde varyans analizi ile, uygulama yıllarına ilişkin ortalama verim değerleri yıllar arasında, t-testi ile karşılaştırılmışlardır. Varyans analizi sonuçlarına göre, konular arasında istatistiksel anlamda farklılık saptanamamış, ancak, sulanan konular ile geleneksel konu, varyans analizi ile karşılaştırılmış, 2001 yılında çıtlama oranları bakımından 0.05 düzeyinde önemli farklılıklar bulunmuştur. İç meyve randımanı bakımından, 2002 yılında geleneksel konu ile sulanan konu ortalamaları arasında önemli farklılık bulunmuştur.

Sulama suyu kullanma randımanları yıllara ve deneme konularına bağlı olarak farklılıklar göstermiştir. Buna göre, konularda sulama suyu ve toplam su kullanma randımanları ile su düzeyleri arasında karşıt bir ilişki bulunmaktadır. Her iki randıman hesabında da, su miktarı azaldıkça değinilen katsayılar artmıştır.

Çalışmada, sulama suyu kullanma oranı sulama suyuna bağlı olarak, TWUE ve IWUE değerlerinin aksine artan bir eğilim göstermektedir. Sulama suyu miktarının artmasıyla, sulama suyunun ET içerisindeki kullanım etkinliği de artmaktadır.

Antepfistığı meyve verimleri ile evapotranspirasyon değerleri arasında doğrusal ilişkiler elde edilmiş, bunlardan yararlanılarak oransal su tüketim azalışlarına karşı oransal verim azalışlarını gösteren verim tepki etmeni (K_y) hesaplanmıştır. Anılan katsayılar, antepfistığında verim tepki etmeni, bütün deneme süresi dikkate alınarak yapılan hesaplamada, 0.95 olarak elde edilmiştir. Ancak, antepfistığı periyodisite gösteren sert çekirdekli meyve çeşidi olduğundan, verim tepki etmeni hem verim hem de periyodisite yıllarında ayrı ayrı hesaplanmış ve K_y değerlerinin sırasıyla 0.80 ve 1.09 olduğu saptanmıştır. Buradan hareketle, antepfistığı periyodisite yıllarında verim yıllarına göre, suya karşı daha duyarlı iken; verim yıllarında su eksikliğinden ileri gelen gerilime karşı daha dayanıklı olduğu söylenebilir.

5.2. Öneriler

Yukarıda özetlenen sonuçlar ışığında, aşağıda sıralanan öneriler yapılabilir.

- Antepfistığı, Güneydoğu Anadolu bölge ekonomisinde çok önemli bir konuma sahiptir. Dünya üretimi sıralamasında ve ihracattaki konumu dikkate alınarak antepfistığına ilişkin sorunlar daha ayrıntılı ve özel çalışmalarla araştırılmalı, çözüme katkı sağlayacak çalışmalar devam ettirilmelidir.
- Antepfistığında sulama konusu, daha ayrıntılı çalışmalarla desteklenmeli, sulamanın verim üzerindeki olumlu etkisi nedeniyle yağışın çok az veya hiç olmadığı yaz aylarında mutlaka sulama uygulamaları yapılmalıdır. Bu

şekilde, yetiştiriciler tarafından daha önceleri kazanılmış olan yanlış ve eksik bilgiler ortadan kaldırılmaya çalışılmalıdır.

- Antepfıstığında yapılacak olan sulama uygulamaları için, damla sulama tekniği kullanılabilir. Sulama yapılacak olan materyalin fidan veya yetişkin ağaç olma durumuna göre, tek veya çift lateral kullanılarak sulama suyundan optimum tasarruf sağlanabilir.

- Sulama uygulamaları, deneme sonuçlarına göre en az 15 gün aralıklarla tekrarlanmalıdır. Sulama suyu miktarının hesaplanmasında açık su yüzeyi buharlaşma değerleri kullanılabilir. Bu amaçla, bitki-pan katsayısı olarak, Kcp= 0.60 kullanılabilir.

- Antepfıstığından istenen düzeyde verim alınabilmesi için, gübreleme kesinlikle yapılmalıdır. Bu amaçla, saf madde olarak, 5.74 g/m^2 azot sulama suyu ile birlikte uygulanabilir.

- Su eksikliğine karşı bütün sulanan bitkiler belirli düzeylerde tepki göstermektedirler. Antepfıstığı için bu çalışmada belirlenen dört yıllık ortalama verim tepki etmeni (K_y) olarak 0.95 iken, verim ve periyodisite yıllarında 0.80 ve 1.09 bulunmuştur. Buna göre, antepfıstığı sulama programlarının oluşturulmasında verim ve periyodisite yıllarında belirlenen katsayıların kullanılması önerilebilir.

- Antepfıstığında periyodisiteyi ortadan kaldıracak veya şiddetini en az düzeye indirebilmek için, yeni kurulacak antepfıstığı bahçelerinin, periyodisite eğilimi daha düşük çeşitlerden veya bu çeşitler içinden seçilecek düzenli verim eğilimine sahip ağaçlardan tesis edilmelidir. Bu şekilde, antepfıstığının en büyük sorunlarından olan düzensiz verim ortadan kaldırılabilecek veya şiddeti azaltılabilecektir. Dolayısıyla milli ekonomiye daha fazla katkı sağlamış olacaktır.

KAYNAKLAR

AK, B.E., KAŞKA, N. 1992. Antepfistıklarında Görülen Periyodisite Sorunu, Nedenleri ve Değişik Çeşitlerdeki Durumu. Türkiye I. Ulusal Bahçe Bitkileri Kongresi, Cilt I (Meyve), İzmir, s: 67-72

--------, 1998. The Yield and Fruit Quality of Pistacia vera cv. Siirt Grown at the Ceylanpınar State Farm. Acta Hortic. 470: 510-515

---------, KAŞKA, N., AÇAR, İ., 1999. Dünyada ve GAP Bölgesinde Antepfistığı (Pistacia vera L.) Üretimi, Yetiştirme ve İşleme Yöntemlerinin Karşılaştırılması. GAP I. Tarım Kongresi. Şanlıurfa

AKÇA, E., SAYDAM, C., STEDUTO, P., ZDRULI, P., SARIYEV, A., ÇELİK,İ., KAPUR, S., 2003. Inherited Agroscapes of the Mediterranean: Carbon Pools for Sustainable Natural Resource Management. Proceedings of the MEDCOASTLAND Workshop held in Adana (2-7June 2003), University of Cukurova (in Press).

ALLEN, R.G., PEREIRA, L.S., RAES, D., SMITH, M., 1998. Crop Evapotranspiration. Guidelines for Computing Crop Water Requirements. FAO United Nations Irrigation and Drainage Paper 56 Rome. 301 s

ANONİM., 2002. Gaziantep Tarım Master Planı. Tarım ve Köy İşleri Bakanlığı. Gaziantep Tarım İl Müdürlüğü. Gaziantep, 165 s

ARPACI, S., ATLI, H.S., TEKİN, H., UYGUR, N., 1997. Kuru koşullarda Antepfistıklarında Değişik Sıra Üzeri Mesafeli Dikimlerde Gelişme, Verim ve Bazı Kalite Özelliklerinin İncelenmesi. Antepfistığı Araştırma Enstitüsü Müdürlüğü. Yayın No: 8, Gaziantep, 28 s

ASHWORTH, L.J., GAONA, S.A., SURBER, E., 1985. Nutritional Diseases of Pistachio Trees. Potasyum and Phosphorus Deficiencies and Chloride Boron Toxicities. Phytopathology 75: 1084-1091

ATLI, H.S., ARPACI, S., AYANOĞLU, H., 1998. Antepfistığı Yabani Türlerinin Türkiye' de Yayılış Alanları. IPGRI Meeting. Jordan, s: 35-40

--------, AÇAR, İ., 2001. Antepfistığı Yetiştiriciliği. Antepfistığı Araştırma Enstitüsü Müdürlüğü. Yayın No: 13, Gaziantep, 132 s.

AYFER, M., 1990. Antepfistığının Dünyada ve Türkiye'deki Dünü, Bugünü ve Geleceği, Gaziantep, s: 14-25

AYDENİZ, A., 1990. Fıstıkta Verimliliğe Gübrelemenin Katkısı. Türkiye I. Antepfistığı Sempozyumu Bildirileri, Gaziantep, s: 108-119

BAŞTUĞ, R., KANBER, R., 1989. Sulama Programlarının Geliştirilmesinde Bitkilerin İçsel Su Durumlarını Belirleyen Yöntemlerden Yararlanma Olanakları. Ak. Üni. Zir. Fak. Dergisi. 1989 2(1): 17-30

BOHM, W., 1979. Methods of Studying Root Systems. Ecological Studies 33, Göttingen. 115 s

BROWN, P.H., 1998. Diagnosıng and Correctıng Nutrıent Deficiencies. Economic Consideratıon ın Pistachio Productıon. 18-29 Mayıs 1998. Adana, s: 95-100

BİLGEL, L., DAĞDEVİREN, İ., NACAR, A.S., 1999. Gap Bölgesi Harran Ovası Koşullarında Antepfistığının (Siirt Çeşidi) Su Tüketiminin ve Sulama Programının Belirlenmesi. Türkiye III. Bahçe Bitkileri Kongresi. 14-17 Eylül 1999. Ankara, s: 252-257

BİLGEN, A.M. 1973. Antepfistığı. Gıda, Tarım ve Hayvancılık Bak. Basın Yayın ve Halkla İlişkiler Dairesi Yay., Ankara, 123 s

---------,KAŞKA, N., GEZEREL, Ö., 1981. Sistemik Sıvı Gübrelerin Antepfistıklarında Verim ve Kalite Üzerine Etkileri. Antepfistığı Araştırma Enstitüsü Müdürlüğü. Sonuç Raporu. Gaziantep, 8 s

---------,1982. Verim Çağındaki Antepfistıklarında, Normal Bakım Tedbirleri Altında Sulamanın Miktar ve Zamanının Verim ve Kaliteye Etkisinin Araştırılması, (Yayınlanmamış) Antepfistığı Araştırma Enst. Müd, Gaziantep, 35 s

CARUSO, T., 2001. The Description of the Pistachio Trees.Dipartimento di Arboricoltura, Botanica e Patologia Vegetale Universita *Fedrico II*, Napoli Italy, Abstract Artical.

COOPER, W.S., GREGORY, P.J., TULLY, D., HARRİS, H.C., 1987. İmproving Water Use Efficiency of Annual Crops in the Rainfed Farming Systems of West and North Africa. Exp. Agric. 23, 113-158

CRANE, J.C., NELSON, M.M., 1971. The Unusual Mechanism of Alternate Bearing in the Pistachio. HortScience, Vol.6 (5): 489-490

---------, 1972. Effects of Crop Load, Girdling and Auxin Application on Alternate Bearing in the Pistachio. Hort. Sci. 97: 337-339

---------,CATLIN, P.B., AL-SHALAN, I., 1976. Carbohydrate Levels in Pistachio as Releated to Alternate Bearing. J. Amer. Soc. Hort. Sci., 101(4): 371-374

--------,1977. Pistachio Nuts. Department of Pomology, University of California, Davis, s: 572-603

--------,IWAKIRİ, B.T., 1986. Pistachio Yield and Quality as Affected by Rootstock. J. Amer.Soc. Hort. Sci. 21(5):1139-1140.

CRIDDLE, W.D., DAVIS, S., PAİR, C.H., SHOCKLEY, D.G., 1956. Methods for Evaluating Irrigation Systems. USDA-SCS, No:82; 24 s

DE PALMA, L., NOVELLO, V., 1997. Effect of Drip Irrigation on Leaf-Gas Exchanges and Stem Water Potential in Pistachio cv.Larnaka. II. İnternational Symposiumon Pistachio and Almonds. ISHS Acta Horticulture 470: 317-323

DOORENBOS, J., PRUITT, W.O., 1977. Guidelines for Predicting Crop Water Reqirements. FAO United Nations Irrigation and Drainage Paper 24, Rome

---------,1984. Guidelines for Predicting Crop Water Reqirements. FAO United Nations Irrigation and Drainage Paper 24, Rome 156 s

---------,KASSAM, A.H., 1979. Yield Responds to Water. FAO United Nations Irrigation and Drainage Paper. 33, Rome, 193 s

DURZAN, D.J., 1996. Nitrojen Reallocation During Female Bud Abscission in Alternate Bearing of Pistachio. J. Amer.Soc. Hort. Sci. 10(1996):219-225.

ERGENE, A.S., 1982. Toprak Biliminin Esasları. Atatürk Üni. Ziraat Fak. Yayın No: 267. Erzurum, 366 s.

FEREEMAN, M., FERGUSON, L., 1995. Factors Effecting Splitting and Blanking. California Pistachio Industry. Annual Report 1994-95. s: 106-110

FERGUSON, L., J. MARANTO., R. BEED., 1995. Mechanical Topping Mitigates Alternate Bearing of Kerman Pistachios. Hort Science, 30: 1369-1372

--------,GRIEVE, C., POSS, J.,WILSON, C., CROSS, E., DONOVAN, T., GRATTAN, S., SANDEN, B., REYES, H.C., 2001. Pistachio Rootstocks Salinity Tolerans Tank Trial. Annual Report of California Pistachio Industry, Crop Year 2000-2001, s: 108-109.

FAO, 2003. Food and Agriculturel Organization, Web Page: http://www.fao.org

FİRUZEH, P., LUDDERS, P., 1978. Pistachio Growing in Iran. Hort. Abstract, 49: 282

GAİB, 2003. Gaziantep İhracatçı Birlikleri. İhracat Bülteni Gaziantep, 7 s

GEURTS, F., 1982. The pistachio Nut (Pistachio vera L.), Aspects Related to Germplasm Conservation. Royal Tropical Inst, Amsterdam, 32 s

GOLDBERG, D., GORNAT, B., BAR-YOSEF, B., 1971. The Distrubution Of Roots, Water and Minerals as a Result of Trickle İrrigation. J. Amer. Soc. Hort. Sci., 96(5): 645-648

GOLDHAMER, D.A.,KJELGREN, R.K., BEEDE, R., WILLIAMS, L., MOORE, J.M., LANE, J., WEİNBERGER, G., MENEZES, J., 1985. Water Use Requirements of Pistachio Trees and Response to Water Stres. California Pistachio Industry. Annual Report 1984-85, s. 85-92.

---------,B.C. PHENE., R. BEEDE., L. SCHERLIN., J. BRAZIL., R.K. KJELGREN., D. ROSE. 1986. Water Management Studies of Pistachio: 1. Tree Performance After Two Years of Sustained Deficit Irrigation. Annual Report of California Pistachio Industry, 1985-86 Crop Year, s: 104-109.

---------,PHENE., R., MACDONALD, J., FIERO, S., BEEDE, R. 1989 Influence of Irrigation Method on the Distibution of Roots and Water in a High Verticillium Pistachio Orchard. Annual Report of California Pistachio Industry, Crop Year, s: 97-98.

--------,BEEDE, R.,. 1993. Results of Four Years of Regulated Deficit Irrigation On Deep Rooted Pistachio Trees. California Pistachio Industry. Annual Report 1992-93, s: 107-110.

---------,1995. Irrigation Management. Annual Report of California Pistachio Industry, Crop Year 1994-1995, s: 71-82.

---------,MICHAILIDIES, T.J., MORGAN, D.P., 1997. Buried Drıp İrrigation Reduces Alternaria Late Blıght in Pistachio. II. İnternational Symposiumon İrrigation of Horticultural Crops. ISHS Acta Horticulture 449: Abstract

HAMDY, A.,1995. Fertilizers and their Efficent Use. Advenced Short Course on Fertigation, Beırut, Lebanon, s: 83-139.

HENDRICKS, L., FERGUSON, L., 1995. The Pistachio Tree. California Pistachio Industry, Annual Report 1994-95, s: 7-10.

IAEA., 2002. Neutron and Gamma Probes: Their Use in Agronomy. International Atomic Energy Agency Training Course Series 16 Vienna, 74 s

JAMES, L.G., 1988. Principles of Farm Irrıgatıon System Design. John Wiley and Sons Inc., New York, 543 s

JENSEN, M.E., BURMAN, R.D., ALLEN, R.G (edt.)., 1990. Evapotranspiration and Irrıgatıon Water Requirements. ASAE Rep. No.70, New York, 331s

KAÇAR, B., 1972. Bitki ve Toprağın Kimyasal Analizleri. II. Bitki Analizleri. A.Ü.Z.F. Yayın No: 453, Ankara, 646 s

KANBER, R. 1984. Çukurova Koşullarında Açık Su Yüzeyi Buharlaşmasında (Class A Pan) Yararlanarak Birinci ve İkinci Ürün Yerfıstığının Sulanması. Tarsus Bölge Topraksu Araştırma Enstitüsü Müdürlüğü Yayınları, Genel Yayın No: 114, Rapor Seri No: 64, 93 s.

---------,EYLEN, M., TOK, A., 1986. Çukurova Koşullarında Karık ve Damla Sulama Yöntemleri ile Sulanan Çileğin Verim ve Su Tüketimi. Tarsus Köyhizmetleri Araştırma Enstitüsü Müdürlüğü Yayınları, Genel Yayın No: 135, Rapor Seri No: 77, 39 s.

---------,EYLEN, M., YÜKSEK, G., 1986. Pistachio Yield and Evapotranspiration Under Urfa-Birecik Conditions (Turkish). Proc. In The Second National Kulturteknik Congress, Univ. Of Cukurova, Agric. Faculty, s: 312-355.

---------,EYLEN, M., KÖKSAL, H., ve YÜKSEL, G. 1990. Güneydoğu Anadolu Koşullarında Antepfıstığı Verim ve Su tüketiminin İrdelemesi. Türkiye 1. Antepfıstığı Simpozyumu, Gaziantep, s: 145-160

---------,YAZAR, A., ÖNDER , S., KÖKSAL, H., 1992. Irrigation Response of Pistachio (Pistacia vera L.). Irrig. Sci. 14:7-14

---------,KIRDA, C., YAZAR, A., ÖNDER, S., KÖKSAL, H.,1993. Irrigation Response of old Pistachio (Pistachio vera L.) Doğa-Tr.J. of Agriculturel and Forestry, 17 (1993), 659-671

---------,EYLEN, M., 1995. Turunçgillerde Su-Verim İlişkileri. Türkiye II. Ulusal Bahçe Bitkileri Kongresi. Adana, s: 550-554

--------,KÖKSAL, H., ÖNDER, S., EYLEN, M., 1996a. Farklı Sulama Yöntemlerinin Genç Portakal Ağaçlarında Verim, Kök Gelişimi ve Su Tüketimine Etkileri. Tr.J.Agriculture and Forestry 20 (1996) 163-172

--------,KÖKSAL, H., ÖNDER, S., ÜNLÜ, M., SEZEN, S.M., ÖZEKİNCİ, B., YAZAR, A., PAKYÜREK, Y., 1996b. Bazı Kışlık Sebze Türlerinde Sulama Olanaklarının Araştırılması. Ç.Ü.Z.F. Genel Yayın No:154, GAP Yayınları No: 195, Sonuç Raporu, Adana, 83 s.

--------,1997. Sulama. Cukurova Üni. Ziraat Fak. Yayın No. 174, Genel Yayın No: 52, Adana, 530 s

--------,ÜNLÜ, M., ÖDEMİŞ, B., DİKER, K., 1999. Pan Evaporation Methods Used for Estimation of Crop Evapotranspiration. "in Workshop on Experimental Methodologies for Determining Evapotranspiration: Review and Revision" 6-12 Aralık 1998 Adana, Turkiye, 211 s

---------,YÜKÇEKEN, Y., KÖKSAL, H., ÖNDER, S., ÜNLÜ, M., SEZEN, S.M., ÖZEKİNCİ, B., 2000. Damla sulama Yöntemiyle Fertigasyon Uygulamalarının Antepfıstığında Gelişme, Verim ve Periyodisiteye Etkisinin İncelenmesi. Ç.Ü. Ziraat Fakültesi, Tar. Yap. ve Sul. Böl. Ara Sonuç Raporu, Adana, 20 s

--------, KÖKSAL, H., ÜNLÜ, M., 2004. Nevşehir Yöresinde Farklı Sulama yöntemleriyle sıvı gübre uygulamalarının (Fertigasyon) Patates Verimi ve Azot kullanımına Etkileri. TÜBİTAK, Tarım-Orman ve Gıda Teknolojileri Araştırma Gurubu, (Sonuç Raporu), 108 s

KARACA, R., NİZAMOĞLU, A., 1988. Aynı Bakım ve Toprak Şartlarında Yerli ve Yabancı Bazı Antepfıstığı Çeşitlerinin Bazı Kalite Özelliklerinin Araştırılması. Antepfıstığı Araştırma Enstitüsü Müdürlüğü. Gaziantep (Sonuç Raporu- Basılmamış), 37 s

KAŞKA, N., 1998a. The Pistachio Its Traditional Growing Areas. Advanced Course Production and Economics of Nut Crops, Adana, Turkey,

---------,1998b. Post Harvest Management of Pistachio Nuts: The Case of the Mediterranean Countries. Advanced Course Production and Economics of Nut Crops, Adana, Turkey.

--------,2001. The Pistachio Growing in The Mediterranean Basin. Proceedings of the Third International Symposium on Pistachio and Almonds. 20-24 Mayıs 2001, Zaragoza, Spain, s: 443-451

KIRDA, C., BAYTORUN, N., DERICI, R., DAŞGAN, Y., 1997. Kimi Örtü altı Bitkilerinde Fertigasyon Uygulamalarında Su-Azot-Verim İlişkisinin Belirlenmesi. Ç.Ü. Ziraat Fak. Tar. Yap. ve Sulama Bölümü. TUBİTAK TOGTAG-1272 Araştırma Projesi. Adana, 69 s

KIRNAK, H., AK, B.E., AÇAR, İ., 1999. İrrigation and İrrigation Management Strategies of Pistachio Orchards. XI. Grempa Seminar on Pistacios and Almonds. Chairs Options Mediterraneennes. Vol. 56. Şanlıurfa, s: 271-278

KIZILGÖZ, İ., KIZILKAYA, R., AÇAR, İ., KAPTAN, H., 1999. Nutrient content of pistachio trees (Pistacia vera L.) gowing in district of Şanlıurfa and the releationship between their microelement deficiency and some soil proporties. XI. Grempa Seminar on Pistacios and Almonds. Chairs Options Mediterraneennes. Vol. 56. Şanlıurfa, s: 47-52

KOUMANOV, K.S., HOPMANS, J.W., SCHWANKL L.J., ANDREU, L., TULİ, A., 1997. Application Efficiency of micro-Sprinkler Irrigation of Almonds Trees. Agricultural Water Management 34:247-263

KÖKSAL, H., TARI, A.F., ÇAKIR, R., KANBER, R.,ÜNLÜ, M., 2001. Su-Verim İlişkileri (Değiştirilmiş 2. Baskı). Köy Hizmetleri Ana Projesi (435-1). Konya. 87 s.

KURU, C., 1992. Antepfıstığı: Dikimden Hasada. Ar Ajans, Gaziantep. 102 s.

KÜDEN, A.B., KAŞKA, N., AK, B.E., KURU, C., TEKİN, H., 1992. The Chilling Requirements and the Growing Degree Hours of Some Pistacio Nut Varieties. First National Horticultural Congres of Turkey. October 13-16, 1992, İzmir, s: 73-77

---------,KAŞKA, N., TANRIVER, E., AK, B.E., TEKİN, H., 1995. Determining the Chilling Requirements and Growing Degree Hours of Some Pistacio Nut Cultivars and Regions. Acta Horticulturae 419, s: 85-90

LEWIN, I., ASSAF R., BRAVDO, B., 1972. Effect of Irrigation for Apples Trees on Water Uptake From Different Soil Layers. J. Am. Hortic. Sci. 87:521-526

LOVATT, J.C., FERGUSON, L. 1997. Using Foliar applications of Urea Combined with 6-Benzyladenine (Ba) to Decrease Pistachio Floral Bud Abscission in an "on" Year to İncrease Yield the Next Year. California Pistachio Industry, Crop Year 1996-1997. s: 148-151.

---------,2001. Efficacy Data for Use Combined with 6-Benzyladenin (Ba) in 100 Gallons of Water per Acre for Registration of Ba to Reduce Alternate Bearing in Pistachio and Increase Cumulative Yield. Annual Report Crop Year 2001-2002, California Pistachio Commission, s: 91-92.

Mac DONALD, J., GOLDHAMER, D., BOLKAN, L., BEEDE, R., 1992. İnfluence of İrrigation Method on Distrubition of water, roots and Verticillium dahliae microscleretoria in a young Pistachio Orchard. California Pistachio İndustry Annual Report.

MARANTO, J., CRANE, C. J., 1982. Pistachio Production. Cooperative Extention, Unv. of Calf. Oakland. California,

MARRA, F.P., BARONE, E., MOTİSİ, A., SİDARİ, M., CARUSO, T., 1997. Dry Matter Accumalation and Carbohydrate Content within Branches of Fruiting and Deblossomed Pistachio (Pistacia Vera L.) Trees. Proceedings of the Second International Symposium on Pistachio and Almonds. Acta Horticulture. Davis-California. 24-29 Ağust 1997, 470: 331-340

MATTOS, Jr. D., ALVA, A.K., PARAMASİVAM, S., GRAETZ, D.A., 2003. Nitrogen Volatilization and Mineralization in a Sandy Entisol of Florida under Citrus. Cominications in Soil Science and Plant Analysis, 34 (13-14): 1803-1824

METHENEY, P.D., REYES, H.C., FERGUSON, L., 1998. Belended Drainage Water Irrigation of Pistachios, cv Kerman on Four Rootstocks in the Southern San Joaquin Valley of California. Proceedings of the Second

International Symposium on Pistachio and Almonds. (http://www.actahort.org/books/470/470_69.htm)

MICHAILIDES, T.J., MORGAN, D.P., 1991. An Integrated Approach for Control of Late Blight Caused by Alternaria Alternate and Management of Mycoflora of Pistachio Through Manipulation of Irrigation Practices. Annual Report of California Pistachio Industry,1990-91 Crop Year, s: 59-65

MONASTRA, F., AVANZATO, D., MARTELLİ, S., DASCANİO, R. 1997. Irrıgatıon of Pistachio in Italy: Ten Years of Observation. Proceedings of the Second International Symposium on Pistachio and Almonds. Acta Horticulture. Davis-California. 24-29 Ağust 1997, 470: 516-.

MUNTUBANİ, D.S.N., MARTIN,C.G., NISHIJIMA, C., 1997. Leaf Development, Dry Matter Accumulation, and Distribution within Branches of Alternate-Bearing 'Kerman' Pistachio Trees. J. Amer.Soc. Hort. Sci. 122(1):31-37

OLSSON, K.A., ROSE C.V., 1988. Patterns of Water Withdrawal Beneath of Irrigated Peach Orchard on a Red-Brown Earth. Irrig. Sci. 9: 89-104

OKE, T.R., 1987. Boundary Layer Climates, 2nd Edn, Methuen Co. Ltd, London.

ÖZBEK, S., 1978. Özel Meyvecilik. Ç.Ü. Z.F. Yayın No: 128, Ders Kitabı 11, Adana, 486 s

ÖZMEN, S., 2002. Pistachıo Respons under Different Irrıgatıon and Fertıgatıon Practıces in South East Anatolia Regıon (GAP) of Turkey. Instıtuto Agronomico Mediterraneo di Bari. MSc. Thesis. Bari-Italy, 65 s

PAPADOPOULOS, I., 1995. Fertigation. Micro-irrigation Systems and Fertigation. Advenced Short Course on Fertigation, Beırut, Lebanon, s: 51-83

PARFIT, E.D., 1995. Pistachio Cultivers. California Pistachio Industry. Annual Report 1994-1995, s: 43-47

PETERS, R.G., CALVİN, L.D., 1965. Sampling Methods of Soil Analysis. "Ed. C.A. Blck et al. part 1 Agron. Seri No. 9 ASA Pub. Madison Wisconsin, s: 54-72

PHENE, R. C.,J..MENESIN, JR., D. A. GOLDHAMER, G. J. AITKENS, R. BEEDS, R. KIELGREN., 1985. Irragation Scheduling of Drip Irrigated Pistachio. Drip/Trickle Irrigation In Action. Proceeding Of The Third

International Drip/Trickle Irrigation Congress. Vol. 2., ASAE, St. Joseph, Michigan, s: 805-810

PİCCHİONİ, G.A., MİYAMOTO, S., STOREY, J.B., 1991. Boron Uptake and Effects on Growth and Carbohydrate Partitioning of Pistachio Seedlings. J. Amer. Soc. Hort. Sci. 116 (4): 706-711

PORLINGIS, I.C., 1974. Flower Bud Abscision in Pistachio as Releated to Fruıt Development and Other Factors. J. Amer. Soc. Hort. Sci. 99 (2). 121-125

RICHARDS, L.A., 1954. Diagnosis and İmprovement of Saline and Alkali Soils. United States Department of Agriculture, Agriculture Handbook, No: 60

ROSECRANCE, R.C., WEINBAUM, S.A., BROWN, P.H., 1995. Assesment of Nitrogen, Phosphorus and Potassium Uptake Capacity and Root Growth in Mature Alternate- Bearing Pistachio (Pistacia Vera) Trees. Tree Physiology 16, 949-956

--------,--------,--------, 1997. Paterns of Nitrogen Uptake and Storage in Mature, Alternate Bearing Pistachio Trees. Second ISHS Pistachio Nut Symposium, California, U.S.A. s: 387-393

SCHWANKL, L.J., 1995. Pistachio Production (The Orchard). Cooperative Extention, Unv.of Calf. Oakland. California,

SEPEASKHAH, A.R., MAFTOUN, M., 1981. Growth And Chemical Composition of pistachio Cultivars As Influenced by Irrigation Regimes and Salinity Levels of Irrigation Water 1. Growth, J. Hort. Sci., 56 (4) 277-284

--------,---------, 1982. Growth and Chemical Composition Cultivers as Influenced by Irrigation Regimes and Salinity Levels of Irrigation Water 2. Grovth. J. Hort. Sci., 57 (4) 469-476

--------,MAFTOUN, M., KARIMIAN, N., 1985. Growth and Chemical Composition of Pistachio as Effected by Salınıty and Applied in İran. J. Amer. Soc. Hort. Sci. 60: 115-121

SHEIBANI, A., 1994. Pistachio Production In Iran. First International Symposium on Pistachio Nut, 20-24 Eylül 1994, Adana. Abstract Book, s. 20

SPİEGEL-ROY, P., MAZİGH, D., EVANARİ, M., 1977. Response of Pistachio to Low Soil Moisture Conditions. J. Amer. Soc. Hort. Sci. 102:470-473.

STEDUTO, P.,1984. Fertigation. Riv. di Agron., 18, 13-20

---------,CALİANDRO, A., RUBİNO, P., et all., 1996. Penman-Monteith Reference Evapotranspiration Estimates in the Mediterranean Region. " Evapotranspiration and Irrigation Scheduling ". Proc. Of Int. Conf. Nov. 3-6, 1996. San Antonio, ASAE, the Irr. Assoc. 357-364

---------,SNYDER, R.L., 1998. Options. " IAM-ETo Software Programme and User's Guide" CIHEAM-IAM, Bari, s 49

SYKES,T.J., 1975. The Influence of Climate on The Regional distribution of Nut Crops in Turkey. Economic Botany 29:108-115

ŞENER,S., 1993. Sulama Teknolojisinde Yeni Gelişmeler. Köy Hizmetleri Genel Müdürlüğü, APK Dairesi Başkanlığı, Toprak ve Su Kaynakları Araştırma şube Müdürlüğü. Yayın No: 76, Tarsus, 171 s

TAKEDA, F., CRANE, J.C., 1980. Abscisic Acid in Pistachio as Releated to İnflorence Bud Abscission. J. Amer. Soc. Hort. Sci. 105 (4):573-576

TKB,1993. Antepfistığı Çeşit Kataloğu. Tarım ve Köyişleri Bakanlığı. Tarımsal Üretim ve Geliştirme Genel Müdürlüğü, Genel Yayın No: 361, Seri No: 20. Ankara, 64 s

TEKİN, H., GENÇ, Ç., KURU, C., AKKÖK, F., 1990. Antepfistığı Besin Kapsamlarının Belirlenmesi ve En Uygun Yaprak Örneği Alım Zamanının Tespiti. Türkiye 1. Antepfistığı Sempozyumu. 11-12 Eylül 1990. Gaziantep s. 120-138

--------,1992. Gaziantep Yöresinde Topraktan ve Yapraktan Yapılan Farklı Gübre Uygulamalarının Antepfistığının Yaprak Bileşimi, Gelişme, Verim ve Ürün Kalitesine Etkilerinin Araştırılması. Ç.Ü. Fen Bilimleri Enstitüsü. Doktora Tezi. 91 s.

--------,GÜZEL, N. 1993. Gaziantep Yöresinde Topraktan ve Yapraktan Yapılan Farklı Gübre Uygulamalarının Antepfistığının Yaprak Bileşimi, Gelişime ve Ürün Kalitesine Etkileri. Antepfistığı Araştırma Enstitüsü Yay., No:3, Gaziantep, 17 s.

--------,1993. Türkiye Tarımsal Araştırma Projesi (TAP) Antepfistığı Master Planı Antepfistığı Araştırma Enstitüsü Müdürlüğü, Gaziantep, 41 s

--------,ARPACI, S., YÜKÇEKEN, Y., ÇAKIR, İ. 1997. Pistachio Nut Iron Deficiencies on Calcareous Soils. Second ISHS Pistachio Nut Symposium, 24-29 Ağustos 1997, California, s: 421-425.

--------, ARPACI, S., ATLI, H.S., 2001. Antepfistığı Yetiştiriciliği. Antepfistığı Araştırma Enstitüsü Müdürlüğü. Yayın No: 13, Gaziantep, 132 s.

THOMPSON, B., 2003. Volatilization Losses from Nitrogen Fertilizers. Missisipi Chemical Corporation, Rota, Missisipi, 2 s (www. missichem. Com/scrReports/ cotton.pdf)

TÜZÜNER, A., 1990. Toprak-Su Analiz Rehberi. Tarım ve Orman Bak. Ankara, 375 s

ULUSARAÇ, A.,1993. Antepfistığı Çeşit Kataloğu. Tarım ve Köyişleri Bakanlığı. Mesleki Yayınlar, Genel Yayın No: 361, Seri No: 20, Ankara, 64 s.

URIU, K., CRANE, J.C., 1977. Mineral Element Changes in Pistachio Leaves. . Amer. Soc. Hort. Sci. 102(2): 155-158

---------,PEARSON, J., 1987. Zinc Deficiency in Pistachio Diagnosis and Correctıon. California Pistachio Industry. Annual Report. Crop Year 1986-1987, s: 71-72

ÜNLÜ, M., 2000. Çukurova Koşullarında Mikrometeorolojik Yöntemlerle Pamuk Su Tüketiminin ve Bitki Katsayılarının Belirlenmesi. Çukurova Uni. Fen Bilimleri Ens. Doktora Tezi. 106 s

USDA-SCS., 1967. Irrigation Water Requirements. Tech. Release, No.21, Washington D.C., 83 s

WEINBAUM, S.A.,MURAOKA, T.T., 1989. Nıtrojen Usage and Fertilizer Nitrojen Recovery by Mature Pistachio Trees. Annual Report Crop Year 1988-89. Uni. of California, Davis. 95616. s: 84-86

WEINBAUM, S.A., ROSECRANCE, R.C., 1998. Economic Consideration in Pistachio Production. 18-29. Mayıs 1998. Adana, 103 s

WOOD, B.W., 1989. Pecan Production Responds to Root Carbohydrates and Rootstock. J. Amer. Soc. Hort. Sci. 114(2): 223-228

YURTSEVER, N., 1984. Deneysel İstatistik Metotlar. Tarım ve Köy İşleri Bakanlığı. Köy Hizmetleri Genel Müdürlüğü Yayınları. Genel Yayın No: 121, Teknik Yayın No: 56, Ankara, 574 s

ZENG, D.Q., BROWN, P.H., ROSECRANCE, R.C., 1997. Effects of Crop Load on Potassium Uptake and Partitioning in Pistachio. California Pistachio Commission Web Page. http:// fruitsandnuts. ucdavis.edu/ ishs/ abstract46. html

ZENG, D.Q., BROWN, P.H., HOLTZ, B.A.,1999. Potassium Fertilization and Diagnostic Criteria for Pistachio Trees. California Pistachio Commission Better Crops/ California,Vol. 83, s: 10-12

ZHANG, Q., BROWN, P.H.1999a. Distribution and Transport of Foliar Applied Zinc in Pistachio. J. Amer. Soc. Hort. Sci. 124(4): 433-436

--------,1999b.The Mechanisim of Foliar Zinc Absorption in Pistachio and Walnut. J. Amer. Soc. Hort. Sci. 124(3): 312-317

ÖZGEÇMİŞ

1964 yılında Gaziantep İli Nizip İlçesinde doğdu. İlk ve Orta öğrenimini Nizip'te, Lise öğrenimini ise K.Maraş'ta tamamladı. 1987 yılında Erzurum Atatürk Üniversitesi Ziraat Fakültesi Kültürteknik Bölümünden mezun oldu. 1988 yılında askerlik görevini tamamladı. 1990-1991 yılında 1 yıl süre ile Şanlıurfa Harran Ovası Arazi Toplulaştırma projesinde çalıştı. 1993 yılında Tarım Bakanlığı Karkamış İlçe Müdürlüğüne atandı. Ç.Ü. Ziraat Fakültesi Tarımsal Yapılar ve Sulama Bölümünde " Antepfıstığı Çöğürlerinde Farklı Sulama Programlarının Gövde Gelişmesi ve Su Tüketimine Etkilerinin İrdelenmesi" konulu Yüksek Lisans çalışmasını 1998 yılında tamamladı. 6-18 Eylül 1999' da Settat-Morocco' da " Water Use Efficiency" konulu, 8 Ocak- 10 Şubat 2001 tarihleri arasında İtalya Bari' de bulunan "Mediterranean Agronomic Institutes" de "Bitki-Atmosfer İlişkileri" konulu kurslara katıldı. Eylül/ 2001 döneminde Çukurova Üniversitesi Fen Bilimleri Enstitüsü Tarımsal Yapılar ve Sulama Anabilim Dalı'nda doktora programına başladı. 1994 yılından beri Antepfıstığı Araştırma Enstitüsünde, Yetiştirme Tekniği Şubesinde çalışmakta olan araştırmacı, evli ve 2 çocuk babasıdır.

EKLER

Ek Çizelge 1. $7N_360$ Konusunda Su Bütçesi Yöntemine Göre Belirlenen Yığışımlı Su Tüketimi Değerleri (1999)

Tarih	S1	P	IR	Top	Dp	Rf	S2	Etper	Etcum
29.04.1999	432.9	0	0.0	432.9	0	0	405.8	27.1	0
13.05.1999	405.8	5	11.8	422.6	0	0	390	32.6	27.1
20.05.1999	390.0	0.5	27.6	418.1	0	0	392.4	25.7	59.7
27.05.1999	392.4	0	42.0	434.4	0	0	401.3	33.1	85.3
03.06.1999	401.3	0	21.9	423.2	0	0	390.6	32.6	118.4
10.06.1999	390.6	0	44.7	435.3	0	0	395.6	39.7	151.0
17.06.1999	395.6	5	32.7	433.3	0	0	390.5	42.8	190.8
24.06.1999	390.5	0	50.1	440.6	0	0	399.4	41.2	233.6
01.07.1999	399.4	0	44.8	444.2	0	0	385.2	59.0	274.8
08.07.1999	385.2	0	50.0	435.2	0	0	390.2	45.0	333.8
15.07.1999	390.2	0	49.1	439.3	0	0	389.6	49.7	378.8
22.07.1999	389.6	0.2	48.5	438.3	0	0	396.8	41.5	428.5
29.07.1999	396.8	0	49.5	446.3	0	0	398.6	47.7	470.0
05.08.1999	398.6	0	44.9	443.5	0	0	384.2	59.3	517.7
12.08.1999	384.2	0	38.1	422.3	0	0	375.6	46.7	577.0
19.08.1999	375.6	0	37.5	413.1	0	0	362.3	50.8	623.7
26.08.1999	362.3	0	37.5	399.8	0	0	351.3	48.5	674.5
02.09.1999	351.3	0	35.0	386.3	0	0	355	31.3	723.1
07.11.1999	355.0	0							754.3
Toplam	77.9	10.7	665.7						
			754.3					754.3	

Ek Çizelge 2. $7N_390$ Konusunda Su Bütçesi Yöntemine Göre Belirlenen Yığışımlı Su Tüketimi Değerleri (1999)

Tarih	S1	P	IR	Top	Dp	Rf	S2	Etper	Etcum
29.04.1999	412.9	0	0.0	412.9	0	0	400.8	12.1	0.0
13.05.1999	400.8	5	17.6	423.4	0	0	390.0	33.4	12.1
20.05.1999	390.0	0.5	41.4	431.9	0	0	400.0	31.9	45.5
27.05.1999	400.0	0	63.0	463.0	0	0	430.0	33.0	77.4
03.06.1999	430.0	0	32.8	462.8	0	0	415.6	47.2	110.4
10.06.1999	415.6	0	67.1	482.7	0	0	425.2	57.5	157.6
17.06.1999	425.2	5	49.1	479.3	0	0	432.5	46.8	215.1
24.06.1999	432.5	0	75.1	507.6	0	0	421.3	86.3	262.0
01.07.1999	421.3	0	67.2	488.5	0	0	409.8	78.7	348.3
08.07.1999	409.8	0	75.0	484.8	0	0	404.3	80.5	427.0
15.07.1999	404.3	0	73.6	477.9	0	0	395.4	82.5	507.5
22.07.1999	395.4	0.2	72.8	468.4	0	0	390.2	78.2	590.0
29.07.1999	390.2	0	74.3	464.5	0	0	406.7	57.8	668.2
05.08.1999	406.7	0	67.3	474.0	0	0	400.2	73.8	726.0
12.08.1999	400.2	0	57.1	457.3	0	0	395.3	62.0	799.8
19.08.1999	395.3	0	56.3	451.6	0	0	390.8	60.8	861.8
26.08.1999	390.8	0	56.3	447.1	0	0	385.2	61.9	922.6
02.09.1999	385.2	0	52.4	437.6	0	0	346.0	91.6	984.5
07.11.1999	346.0	0							1076.2
Toplam	66.9	10.7	998.6						
			1076.2					1076.2	

Ek Çizelge 3. 14$N_3$60 Konusunda Su Bütçesi Yöntemine Göre Belirlenen Yığışımlı Su Tüketimi Değerleri (1999)

Tarih	S1	P	IR	Top	Dp	Rf	S2	Etper	Etcum
29.04.1999	412.9	0	0.0	412.9	0	0	400.8	12.1	0.0
13.05.1999	400.8	5.5	11.8	418.1	0	0	376.7	41.4	12.1
26.05.1999	376.7	0	42.0	418.7	0	0	385.2	33.5	53.5
09.06.1999	385.2	0	44.7	429.9	0	0	388.7	41.2	86.9
23.06.1999	388.7	5	50.1	443.8	0	0	410.5	33.3	128.2
07.07.1999	410.5	0	50.0	460.5	0	0	415.6	44.9	161.5
21.07.1999	415.6	0.2	48.5	464.3	0	0	395.8	68.5	206.4
04.08.1999	395.8	0	44.9	440.7	0	0	410.3	30.4	274.9
18.08.1999	410.3	0	37.5	447.8	0	0	382.2	65.6	305.3
01.09.1999	382.2	0	35.0	417.2	0	0	320.0	97.2	370.9
07.11.1999	320.0								468.1
									468.1
	92.9	10.7	364.5						
			468.1					468.1	

Ek Çizelge 4. 14$N_3$90 Konusunda Su Bütçesi Yöntemine Göre Belirlenen Yığışımlı Su Tüketimi Değerleri (1999)

Tarih	S1	P	IR	Top	Dp	Rf	S2	Etper	Etcum
29.04.1999	432.9	0	0.0	432.9	0	0	395.3	37.6	0.0
13.05.1999	395.3	5.5	17.6	418.4	0	0	368.5	49.9	37.6
26.05.1999	368.5	0	63.0	431.5	0	0	390.5	41.0	87.5
09.06.1999	390.5	0	67.1	457.6	0	0	395.0	62.6	128.5
23.06.1999	395.0	5	75.1	475.1	0	0	400.2	74.9	191.1
07.07.1999	400.2	0	75.0	475.2	0	0	410.6	64.6	266.0
21.07.1999	410.6	0.2	72.8	483.6	0	0	402.5	81.1	330.7
04.08.1999	402.5	0	67.3	469.8	0	0	393.5	76.3	411.7
18.08.1999	393.5	0	56.3	449.8	0	0	375.6	74.2	488.0
01.09.1999	375.6	0	52.4	428.0	0	0	348.0	80.0	562.3
07.11.1999	348.0								642.3
Toplam	84.9	10.7	546.7						
			642.3					642.3	

Ek Çizelge 5. $7N_360$ Konusunda Su Bütçesi Yöntemine Göre Belirlenen Yığışımlı Su Tüketimi Değerleri (2000)

Tarih	S1	P	IR	Top	Dp	Rf	S2	Etper	Etcum
05.04.2000	386.0	43.1	0	429.1	0	0	410.0	19.1	0
28.04.2000	410.0	10.6	0	420.6	0	0	395.0	25.6	19.1
08.05.2000	395.0	0.0	22.6	417.6	0.0	0.0	380.0	37.6	44.7
15.05.2000	380.0	0.0	26.6	406.6	0.0	0.0	375.0	31.6	82.3
22.05.2000	375.0	0.0	27.6	402.6	0.0	0.0	372.3	30.3	113.8
12.06.2000	372.3	0.0	52.2	424.5	0.0	0.0	398.0	26.5	144.2
19.06.2000	398.0	0.0	53.4	451.4	0.0	0.0	403.2	48.2	170.6
26.06.2000	403.2	0.0	45.9	449.1	0.0	0.0	397.0	52.1	218.8
03.07.2000	397.0	0.0	43.3	440.3	0.0	0.0	404.4	35.9	270.9
10.07.2000	404.4	0.0	57.4	461.8	0.0	0.0	401.2	60.6	306.8
17.07.2000	401.2	0.0	60.0	461.2	0.0	0.0	398.9	62.3	367.4
24.07.2000	398.9	0.0	49.5	448.4	0.0	0.0	398.8	49.6	429.7
31.07.2000	398.8	0.0	46.4	445.2	0.0	0.0	393.0	52.2	479.3
07.08.2000	393.0	0.0	44.9	437.9	0.0	0.0	378.3	59.6	531.5
14.08.2000	378.3	0.0	49.5	427.8	0.0	0.0	380.9	46.9	591.1
21.08.2000	380.9	0.4	37.5	418.8	0.0	0.0	363.3	55.5	638.0
28.08.2000	363.3	0.0	44.9	408.2	0.0	0.0	348.3	59.9	693.6
04.09.2000	348.3	0.0	37.0	385.3	0.0	0.0	336.6	48.7	753.4
11.09.2000	336.6	5.4	38.6	380.6	0.0	0.0	340.5	40.1	802.2
18.09.2000	340.5	1.4	39.0	380.9	0.0	0.0	350.0	30.9	842.2
25.09.2000	350.0	60.9	0.0	410.9	0.0	0.0	357.5	53.4	873.1
21.11.2000	357.5								926.5
Toplam	28.5	121.8	776.2						
			926.5					926.5	

Ek Çizelge 6. 7$N_3$90 Konusunda Su Bütçesi Yöntemine Göre Belirlenen Yığışımlı Su Tüketimi Değerleri (2000)

Tarih	S1	P	IR	Top	Dp	Rf	S2	Etper	Etcum
05.04.2000	481.0	43.1	0	524.1	0	0	470.0	54.1	0
28.04.2000	470.0	10.6	0	480.6	0	0	410.0	70.6	54.1
08.05.2000	410.0	0.0	33.8	443.8	0	0	380.0	63.8	124.7
15.05.2000	380.0	0.0	39.9	419.9	0	0	376.5	43.4	188.5
22.05.2000	376.5	0.0	41.5	418.0	0	0	385.0	33.0	231.9
12.06.2000	385.0	0.0	78.3	463.3	0	0	430.0	33.3	264.9
19.06.2000	430.0	0.0	80.1	510.1	0	0	470.0	40.1	298.1
26.06.2000	470.0	0.0	68.8	538.8	0	0	465.1	73.7	338.2
03.07.2000	465.1	0.0	65.0	530.1	0	0	456.1	74.0	411.9
10.07.2000	456.1	0.0	86.1	542.2	0	0	476.0	66.2	485.9
17.07.2000	476.0	0.0	90.0	566.0	0	0	480.0	86.0	552.1
24.07.2000	480.0	0.0	74.3	554.3	0	0	475.0	79.3	638.0
31.07.2000	475.0	0.0	69.6	544.6	0	0	455.2	89.4	717.3
07.08.2000	455.2	0.0	67.3	522.5	0	0	436.5	86.0	806.8
14.08.2000	436.5	0.0	74.3	510.8	0	0	425.3	85.5	892.8
21.08.2000	425.3	0.4	56.3	482.0	0	0	390.0	92.0	978.3
28.08.2000	390.0	0.0	67.3	457.3	0	0	396.7	60.6	1070.3
04.09.2000	396.7	0.0	55.5	452.2	0	0	362.2	90.0	1130.9
11.09.2000	362.2	5.4	57.8	425.4	0	0	367.2	58.2	1220.9
18.09.2000	367.2	1.4	58.3	426.9	0	0	400.0	26.9	1279.1
25.09.2000	400.0	60.9	0.0	460.9	0	0	362.2	98.7	1306.0
21.11.2000	362.2								1404.7
Toplam	118.8	121.8	1164.1						
			1404.7					1404.7	

Ek Çizelge 7. 14N$_3$60 Konusunda Su Bütçesi Yöntemine Göre Belirlenen Yığışımlı Su Tüketimi Değerleri (2000)

Tarih	S1	P	IR	Top	Dp	Rf	S2	Etper	Etcum
05.04.2000	423.6	43.1	0	466.7	0	0	430.0	36.7	0.0
28.04.2000	430.0	10.6	0	440.6	0	0	415.0	25.6	36.7
08.05.2000	415.0	0	22.6	437.6	0	0	409.2	28.4	62.3
22.05.2000	409.2	0	27.6	436.8	0	0	385.4	51.4	90.7
12.06.2000	385.4	0	53.4	438.8	0	0	398.5	40.3	142.1
26.06.2000	398.5	0	43.3	441.8	0	0	380.4	61.4	182.4
10.07.2000	380.4	0	60.0	440.4	0	0	380.4	60.0	243.8
24.07.2000	380.4	0	46.4	426.8	0	0	341.9	84.9	303.8
07.08.2000	341.9	0	49.5	391.4	0	0	342.0	49.4	388.7
21.08.2000	342.0	0.4	44.9	387.3	0	0	323.3	64.0	438.1
04.09.2000	323.3	5.4	38.6	367.3	0	0	312.0	55.3	502.1
18.09.2000	312.0	1.4	38.9	352.3	0	0	325.7	26.6	557.3
25.09.2000	325.7	60.9	0.0	386.6	0	0	355.3	31.3	583.9
21.11.2000	355.3								615.2
Toplam	68.3	121.8	425.1						
			615.2					615.2	

Ek Çizelge 8. 14N$_3$90 Konusunda Su Bütçesi Yöntemine Göre Belirlenen Yığışımlı Su Tüketimi Değerleri (2000)

Tarih	S1	P	IR	Top	Dp	Rf	S2	Etper	Etcum
05.04.2000	455.3	43.1	0	498.4	0	0	419.0	79.4	0
28.04.2000	419.0	10.6	0	429.6	0	0	400.0	29.6	79.4
08.05.2000	400.0	0	33.8	433.8	0	0	390.0	43.8	109.0
22.05.2000	390.0	0	41.5	431.5	0	0	405.8	25.7	152.8
12.06.2000	405.8	0	80.1	485.9	0	0	420.9	65.0	178.5
26.06.2000	420.9	0	65.0	485.9	0	0	390.0	95.9	243.5
10.07.2000	390.0	0	90.0	480.0	0	0	401.2	78.8	339.3
24.07.2000	401.2	0	69.6	470.8	0	0	388.5	82.3	418.1
07.08.2000	388.5	0	74.3	462.8	0	0	380.2	82.6	500.4
21.08.2000	380.2	0.4	67.3	447.9	0	0	370.0	77.9	583.0
04.09.2000	370.0	5.4	57.8	433.2	0	0	350.0	83.2	660.9
18.09.2000	350.0	1.4	58.3	409.7	0	0	352.9	56.8	744.2
25.09.2000	352.9	60.9	0.0	413.8	0	0	368.2	45.6	801.0
21.11.2000	368.2								846.6
Toplam	87.1	121.8	637.7						
			846.6					846.6	

Ek Çizelge 9. $7N_360$ Konusunda Su Bütçesi Yöntemine Göre Belirlenen Yığışımlı Su Tüketimi Değerleri (2001)

Tarih	S1	P	IR	Top	Dp	Rf	S2	Etper	Etcum
20.04.2001	461.5	16.6	0.0	478.1	0	0	455.0	23.1	0.0
07.05.2001	455.0	34.8	0.0	489.8	0	0	453.9	35.9	23.1
14.05.2001	453.9	5.6	0.0	459.5	0	0	442.0	17.5	59.1
21.05.2001	442.0	0	0.0	442.0	0	0	420.0	22.0	76.5
29.05.2001	420.0	0	39.0	459.0	0	0	415.0	44.0	98.5
05.06.2001	415.0	0	39.1	454.1	0	0	410.0	44.1	142.5
12.06.2001	410.0	0	37.0	447.0	0	0	409.5	37.5	186.6
19.06.2001	409.5	0	39.1	448.6	0	0	389.9	58.7	224.1
26.06.2001	389.9	0	44.9	434.7	0	0	367.6	67.2	282.9
03.07.2001	367.6	0	43.8	411.3	0	0	365.5	45.9	350.0
10.07.2001	365.5	0	44.9	410.3	0	0	336.5	73.8	395.9
17.07.2001	336.5	0	48.0	384.5	0	0	334.0	50.5	469.7
24.07.2001	334.0	0	50.1	384.1	0	0	329.0	55.1	520.2
31.07.2001	329.0	0	45.9	374.9	0	0	321.3	53.6	575.3
07.08.2001	321.3	0	38.1	359.4	0	0	311.2	48.2	628.9
14.08.2001	311.2	0	39.6	350.8	0	0	300.3	50.6	677.1
21.08.2001	300.3	2.2	34.4	336.9	0	0	320.0	16.9	727.7
28.08.2001	320.0	0	37.5	357.5	0	0	330.0	27.5	744.5
04.09.2001	330.0	0	36.0	366.0	0	0	350.7	15.3	772.1
07.09.2001	350.7	25.9	0.0	376.6	0	0	331.3	45.3	787.3
07.11.2001	331.3	0		331.3	0	0	0.0		832.7
Toplam	130.2	85.1	617.3						
			832.7					832.7	

Ek Çizelge 10. $7N_390$ Konusunda Su Bütçesi Yöntemine Göre Belirlenen Yığışımlı Su Tüketimi Değerleri (2001)

Tarih	S1	P	IR	Top	Dp	Rf	S2	Etper	Etcum
20.04.2001	500.7	16.6	0.0	517.3	0	0	431.2	86.1	0.0
07.05.2001	431.2	34.8	0.0	466.0	0	0	452.1	13.9	86.1
14.05.2001	452.1	5.6	0.0	457.7	0	0	434.3	23.4	100.0
21.05.2001	434.3	0	0.0	434.3	0	0	424.3	10.0	123.4
29.05.2001	424.3	0	39.0	463.3	0	0	427.2	36.1	133.4
05.06.2001	427.2	0	58.7	485.8	0	0	435.2	50.6	169.5
12.06.2001	435.2	0	55.5	490.7	0	0	430.3	60.4	220.1
19.06.2001	430.3	0	58.7	489.0	0	0	410.5	78.5	280.6
26.06.2001	410.5	0	67.3	477.8	0	0	399.8	78.0	359.0
03.07.2001	399.8	0	65.7	465.5	0	0	396.4	69.1	437.0
10.07.2001	396.4	0	67.3	463.7	0	0	385.4	78.3	506.1
17.07.2001	385.4	0	72.0	457.4	0	0	375.6	81.8	584.4
24.07.2001	375.6	0	75.1	450.7	0	0	367.6	83.1	666.1
31.07.2001	367.6	0	68.8	436.4	0	0	370.0	66.4	749.3
07.08.2001	370.0	0	57.1	427.1	0	0	387.5	39.6	815.7
14.08.2001	387.5	0	59.5	447.0	0	0	382.7	64.2	855.3
21.08.2001	382.7	2.2	51.6	436.6	0	0	366.7	69.9	919.5
28.08.2001	366.7	0	56.3	423.0	0	0	355.3	67.7	989.4
04.09.2001	355.3	0	54.0	409.3	0	0	385.7	23.6	1057.1
07.09.2001	385.7	25.9		411.6	0	0	340.6	71.0	1080.7
07.11.2001	340.6	0							1151.7
Top	160.1	85.1	906.5						
			1151.7					1151.7	

Ek Çizelge 11. 14$N_3$60 Konusunda Su Bütçesi Yöntemine Göre Belirlenen Yığışımlı Su Tüketimi Değerleri (2001)

Tarih	S1	P	IR	Top	Dp	Rf	S2	Etper	Etcum
20.04.2001	468.1	16.6	0.0	484.7	0	0	445.0	39.6	0.0
07.05.2001	445.0	34.8	0.0	479.8	0	0	439.8	40.0	39.6
14.05.2001	439.8	5.6	0.0	445.4	0	0	430.9	14.5	79.7
21.05.2001	430.9	0	0.0	430.9	0	0	402.4	28.5	94.1
29.05.2001	402.4	0	39.0	441.4	0	0	378.9	62.5	122.6
12.06.2001	378.9	0	37.0	415.9	0	0	355.8	60.1	185.1
26.06.2001	355.8	0	44.9	400.7	0	0	363.5	37.2	245.2
10.07.2001	363.5	0	44.9	408.4	0	0	349.2	59.2	282.4
24.07.2001	349.2	0	50.1	399.3	0	0	336.8	62.5	341.6
07.08.2001	336.8	0	38.1	374.8	0	0	331.4	43.4	404.1
21.08.2001	331.4	2.2	34.4	368.0	0	0	342.2	25.8	447.5
04.09.2001	342.2	0	36.0	378.2	0	0	344.6	33.6	473.3
07.09.2001	344.6	25.9	0.0	370.5	0	0	305.3	65.2	507.0
07.11.2001	305.3	0	0.0		0	0	0.0	0.0	572.1
Toplam	162.8	85.1	324.3						
			572.1					572.1	

Ek Çizelge 12. 14$N_3$90 Konusunda Su Bütçesi Yöntemine Göre Belirlenen Yığışımlı Su Tüketimi Değerleri (2001)

Tarih	S1	P	IR	Top	Dp	Rf	S2	Etper	Etcum
20.04.2001	496.9	16.6	0.0	513.5	0	0	467.6	45.9	0.0
07.05.2001	467.6	34.8	0.0	502.4	0	0	473.6	28.8	45.9
14.05.2001	473.6	5.6	0.0	479.2	0	0	433.5	45.7	74.7
21.05.2001	433.5	0	0.0	433.5	0	0	410.3	23.2	120.4
29.05.2001	410.3	0	39.0	449.3	0	0	405.0	44.3	143.6
12.06.2001	405.0	0	55.5	460.5	0	0	396.5	64.0	187.9
26.06.2001	396.5	0	67.3	463.8	0	0	389.5	74.3	251.9
10.07.2001	389.5	0	67.3	456.8	0	0	385.4	71.4	326.2
24.07.2001	385.4	0	75.1	460.5	0	0	370.7	89.8	397.6
07.08.2001	370.7	0	57.1	427.8	0	0	340.5	87.3	487.4
21.08.2001	340.5	2.2	51.6	394.3	0	0	341.5	52.8	574.8
04.09.2001	341.5	0	54.0	395.5	0	0	341.0	54.5	627.5
07.09.2001	341.0	25.9	0.0	366.9	0	0	332.8	34.1	682.0
07.11.2001	332.8	0	0.0	332.8	0	0	0.0		716.1
Toplam	164.1	85.1	466.9						
			716.1					716.1	

Ek Çizelge 13. $7N_360$ Konusunda Su Bütçesi Yöntemine Göre Belirlenen Yığışımlı Su Tüketimi Değerleri (2002)

Tarih	S1	P	IR	Top	Dp	Rf	S2	Etper	Etcum
18.03.2002	454.9	137.1	0.0	592.0	0.0	0.0	459.3	132.8	0.0
01.05.2002	459.3	3.4	0.0	462.7	0.0	0.0	427.2	35.5	132.8
06.05.2002	427.2	0.0	0.0	427.2	0.0	0.0	402.3	25.0	168.2
13.05.2002	402.3	4.8	0.0	407.1	0.0	0.0	380.9	26.1	193.2
19.05.2002	380.9	25.6	0.0	406.5	0.0	0.0	375.2	31.3	219.3
27.05.2002	375.2	0.0	0.0	375.2	0.0	0.0	350.5	24.7	250.6
04.06.2002	350.5	1.4	58.2	410.1	0.0	0.0	370.0	40.1	275.3
11.06.2002	370.0	0.0	32.3	402.3	0.0	0.0	365.0	37.3	315.4
18.06.2002	365.0	0.0	36.5	401.5	0.0	0.0	360.2	41.3	352.7
25.06.2002	360.2	0.0	41.7	401.9	0.0	0.0	355.2	46.7	394.1
02.07.2002	355.2	0.0	41.7	396.9	0.0	0.0	360.0	36.9	440.8
09.07.2002	360.0	0.0	43.8	403.8	0.0	0.0	363.4	40.4	477.7
16.07.2002	363.4	0.0	34.4	397.8	0.0	0.0	371.0	26.8	518.1
23.07.2002	371.0	0.1	39.1	410.2	0.0	0.0	375.6	34.6	544.9
30.07.2002	375.6	0.0	43.8	419.4	0.0	0.0	369.8	49.6	579.6
06.08.2002	369.8	25.2	41.7	436.7	0.0	0.0	365.7	71.0	629.1
13.08.2002	365.7	0.0	36.5	402.2	0.0	0.0	345.0	57.2	700.2
20.08.2002	345.0	0.0	36.5	381.5	0.0	0.0	340.5	41.0	757.4
27.08.2002	340.5	0.0	31.3	371.8	0.0	0.0	350.0	21.8	798.4
03.09.2002	350.0	0	31.3	381.3	0.0	0.0	348.9	32.4	820.2
09.09.2002	348.9	0.0	28.2	377.1	0.0	0.0	345.6	31.5	852.6
11.09.2002	345.6	0	0.0	345.6	0.0	0.0	335.3	10.3	884.1
23.09.2002	335.3	39	0.0	374.3	0.0	0.0	367.6	6.7	894.4
26.11.2002	367.6		0.0	367.6					901.2
Toplam	87.4	236.6	577.2						
			901.2					901.2	

Ek Çizelge 14. 7N$_3$90 Konusunda Su Bütçesi Yöntemine Göre Belirlenen Yığışımlı Su Tüketimi Değerleri (2002)

Tarih	S1	P	IR	Top	Dp	Rf	S2	Etper	Etcum
18.03.2002	490.9	137.1	0.0	628.0	0	0	470.0	158.0	0.0
01.05.2002	470.0	3.4	0.0	473.4	0	0	410.2	63.2	158.0
06.05.2002	410.2	0.0	0.0	410.2	0	0	380.0	30.2	221.2
13.05.2002	380.0	4.8	0.0	384.8	0	0	355.5	29.3	251.4
19.05.2002	355.5	25.6	0.0	381.1	0	0	355.1	26.0	280.7
27.05.2002	355.1	0.0	0.0	355.1	0	0	330.0	25.1	306.7
04.06.2002	330.0	1.4	58.2	389.6	0	0	340.0	49.6	331.8
11.06.2002	340.0	0.0	48.5	388.5	0	0	335.0	53.5	381.4
18.06.2002	335.0	0.0	54.8	389.8	0	0	330.0	59.8	434.8
25.06.2002	330.0	0.0	62.6	392.6	0	0	361.5	31.1	494.6
02.07.2002	361.5	0.0	62.6	424.1	0	0	355.5	68.6	525.7
09.07.2002	355.5	0.0	65.7	421.2	0	0	370.3	50.9	594.4
16.07.2002	370.3	0.0	51.6	421.9	0	0	365.4	56.5	645.2
23.07.2002	365.4	0.1	58.7	424.2	0	0	383.8	40.4	701.8
30.07.2002	383.8	0.0	65.7	449.5	0	0	395.3	54.2	742.1
06.08.2002	395.3	25.2	62.6	483.1	0	0	433.0	50.2	796.3
13.08.2002	433.0	0.0	54.8	487.7	0	0	410.0	77.7	846.5
20.08.2002	410.0	0.0	54.8	464.8	0	0	417.5	47.3	924.2
27.08.2002	417.5	0.0	47.0	464.5	0	0	410.2	54.3	971.5
03.09.2002	410.2	0.0	47.0	457.2	0	0	405.2	52.0	1025.7
09.09.2002	405.2	0.0	42.3	447.5	0	0	380.3	67.2	1077.7
11.09.2002	380.3	0.0	0.0	380.3	0	0	310.2	70.1	1144.9
23.09.2002	310.2	39.0	0.0	349.2	0	0	336.4	12.8	1215.0
26.11.2002	336.4		0.0	336.4	0	0			1227.8
Toplam	154.5	236.6	836.7						
			1227.8					1227.8	

Ek Çizelge 15. $14N_360$ Konusunda Su Bütçesi Yöntemine Göre Belirlenen Yığışımlı Su Tüketimi Değerleri (2002)

Tarih	S1	P	IR	Top	Dp	Rf	S2	Etper	Etcum
18.03.2002	454.1	137.1	0.0	591.2	0	0	453.2	138.0	0.0
01.05.2002	453.2	3.4	0.0	456.6	0	0	430.2	26.4	138.0
06.05.2002	430.2	0.0	0.0	430.2	0	0	420.2	10.0	164.4
13.05.2002	420.2	4.8	0.0	425.0	0	0	405.4	19.6	174.4
19.05.2002	405.4	25.6	0.0	431.0	0	0	410.3	20.7	194.0
27.05.2002	410.3	0.0	0.0	410.3	0	0	392.1	18.2	214.7
04.06.2002	392.1	1.4	58.2	451.7	0	0	421.2	30.5	232.9
18.06.2002	421.2	0.0	36.5	457.7	0	0	397.4	60.3	263.3
02.07.2002	397.4	0.0	41.7	439.2	0	0	376.3	62.9	323.6
16.07.2002	376.3	0.0	34.4	410.7	0	0	344.3	66.4	386.6
30.07.2002	344.3	0.1	43.8	388.2	0	0	344.4	43.8	452.9
13.08.2002	344.4	25.2	36.5	406.2	0	0	327.2	79.0	496.7
27.08.2002	327.2	0.0	31.3	358.5	0	0	305.2	53.3	575.7
09.09.2002	305.2	0.0	28.2	333.4	0	0	291.6	41.8	628.9
11.09.2002	291.6	0.0	0.0	291.6	0	0	268.2	23.4	670.7
23.09.2002	268.2	39.0	0.0	307.2	0	0	301.1	6.2	694.1
26.11.2002	301.1		0.0	301.1	0	0			700.2
Toplam	153.0	236.6	310.7						
			700.2					700.2	

Ek Çizelge 16. 14N$_3$90 Konusunda Su Bütçesi Yöntemine Göre Belirlenen Yığışımlı Su Tüketimi Değerleri (2002)

Tarih	S1	P	IR	Top	Dp	Rf	S2	Etper	Etcum
18.03.2002	501.2	137.1	0.0	638.3	0	0	453.5	184.8	0.0
01.05.2002	453.5	3.4	0.0	456.9	0	0	440.2	16.7	184.8
06.05.2002	440.2	0.0	0.0	440.2	0	0	420.2	20.0	201.5
13.05.2002	420.2	4.8	0.0	425.0	0	0	405.3	19.7	221.5
19.05.2002	405.3	25.6	0.0	430.9	0	0	410.9	20.0	241.2
27.05.2002	410.9	0.0	0.0	410.9	0	0	397.8	13.1	261.2
04.06.2002	397.8	1.4	58.2	457.4	0	0	396.5	60.9	274.3
18.06.2002	396.5	0.0	54.8	451.3	0	0	390.2	61.1	335.1
02.07.2002	390.2	0.0	62.6	452.8	0	0	389.0	63.8	396.2
16.07.2002	389.0	0.0	51.6	440.7	0	0	373.6	67.1	460.0
30.07.2002	373.6	0.1	65.7	439.4	0	0	342.5	96.9	527.1
13.08.2002	342.5	25.2	54.8	422.5	0	0	349.0	73.5	624.0
27.08.2002	349.0	0.0	47.0	396.0	0	0	342.2	53.8	697.4
09.09.2002	342.2	0.0	42.3	384.5	0	0	338.6	45.9	751.2
11.09.2002	338.6	0.0	0.0	338.6	0	0	325.0	13.6	797.1
23.09.2002	325.0	39.0	0.0	364.0	0	0	341.3	22.7	810.7
26.11.2002	341.3		0.0	341.3					833.4
Toplam	159.9	236.6	436.9						
			833.4					833.4	

Ek Çizelge 17. Konuların Yıllara Göre Verim Değerleri (kg/ağaç)

KONU	YILLAR				ORT.
	1999	2000	2001	2002	
$7N_0 60$	0.81	15.79	7.00	13.38	9.24
$7N_0 90$	1.32	14.12	6.91	9.90	8.06
$7N_1$ 60	0.11	2.96	9.96	4.21	4.31
$7N_1$ 90	0.12	7.44	8.34	3.57	4.86
$7N_2$ 60	0.36	6.68	10.37	4.26	5.41
$7N_2$ 90	0.00	8.14	9.00	3.41	5.14
$7N_3$ 60	0.72	14.15	4.69	6.58	6.53
$7N_3$ 90	1.11	16.02	4.58	8.56	7.56
$14N_0 60$	0.04	3.86	11.74	2.70	4.58
$14N_0 90$	0.08	11.00	12.29	3.78	6.79
$14N_1$ 60	0.40	9.55	8.57	6.82	6.33
$14N_1$ 90	0.00	5.99	7.35	2.92	4.07
$14N_2$ 60	1.66	19.95	5.73	6.93	8.57
$14N_2$ 90	1.03	16.69	5.45	6.93	7.52
$14N_3$ 60	0.08	4.82	10.56	2.66	4.53
$14N_3$ 90	0.54	7.80	12.85	3.50	6.17
Geleneksel	0.45	4.51	9.10	2.80	4.21
Ortalama	0.52	9.97	8.50	5.46	19.90

Ek Çizelge 18. İstatistik olarak önemli bulunan yıllara ilişkin % verim değerleri

KONU	YILLAR							
	2000	Verim %	Verim %	2001	Verim %	Verim %	Ort. Verim	Ort. Verim %
$7N_0 60$	15.79	79.1	75.0	7.00	54.4	54.1	11.39	88.7
$7N_0 90$	14.12	70.8		6.91	53.8		10.52	81.9
$7N_1$ 60	2.96	14.8	26.1	9.96	77.5	71.2	6.46	50.3
$7N_1$ 90	7.44	37.3		8.34	64.9		7.89	61.4
$7N_2$ 60	6.68	33.5	37.1	10.37	80.7	75.3	8.52	66.4
$7N_2$ 90	8.14	40.8		9.00	70.0		8.57	66.7
$7N_3$ 60	14.15	70.9	75.6	4.69	36.5	36.1	9.42	73.3
$7N_3$ 90	16.02	80.3		4.58	35.6		10.30	80.2
14No60	3.86	19.3	37.2	11.74	91.3	93.5	7.80	60.7
14No90	11.00	55.1		12.29	95.6		11.65	90.7
$14N_1$ 60	9.55	47.9	38.9	8.57	66.7	61.9	9.06	70.6
$14N_1$ 90	5.99	30.0		7.35	57.2		6.67	51.9
$14N_2$ 60	19.95	100.0	91.8	5.73	44.6	43.5	12.84	100.0
$14N_2$ 90	16.69	83.6		5.45	42.4		11.07	86.2
$14N_3$ 60	4.82	24.1	31.6	10.56	82.1	91.1	7.69	59.9
$14N_3$ 90	7.80	39.1		12.85	100.0		10.32	80.4
Geleneksel	4.51	22.6	22.6	9.10	70.8	70.8	6.80	53.0

Ek Çizelge 19. Antepfistığı Verim Değerlerine İlişkin Varyans Analizi (1999)

	KT	SD	KO	F	Ft
DT	8.7				
GKT	25.1	31			
B KT	7.8	1	7.8		
N KT	1.6	3	0.5	1.05	
Hata1	1.5	3	0.5		
I KT	0.1	1	0.1	0.04	
I*N int	5.4	3	1.8	1.19	6.59
Hata2	6.1	4	1.5		
Kcp KT	0.00	1	0.0		
Kcp*I int	0.15	1	0.1		
Kcp*N int	1.08	3	0.4		
Kcp*I*N	0.09	3	0.0		
Hata3	1.22	8	0.2		

Ek Çizelge 20. Antepfistığı Verim Değerlerine İlişkin Varyans Analizi (2000)

	KT	SD	KO	F	Ft
DT	3400.0				
GKT	1121.4	31			
B KT	183.8	1	183.8		
N KT	176.6	3	58.9	9.69	
Hata1	18.2	3	6.1		
I KT	4.0	1	4.0	0.21	
I*N int	514.6	3	171.5	8.89*	6.59
Hata2	77.2	4	19.3		
Kcp KT	11.13	1	11.1		
Kcp*I int	1.01	1	1.0		
Kcp*N int	17.63	3	5.9		
Kcp*I*N	81.88	3	27.3		
Hata3	35.39	8	4.4		

Ek Çizelge 21. Antepfistığı Verim Değerlerine İlişkin Varyans Analizi (2001)

Var. Kaynağı	KT	SD	KO	F	Ft
DT	2290.3				
GKT	352.5	31			
B KT	79.9	1	79.9		
N KT	14.6	3	4.9	2.45	
Hata1	5.9	3	2.0		
I KT	23.5	1	23.5	3.60	
I*N int	163.9	3	54.6	8.39*	6.59
Hata2	26.0	4	6.5		
Kcp KT	0.42	1	0.4		
Kcp*I int	2.58	1	2.6		
Kcp*N int	7.48	3	2.5		
Kcp*I*N	1.20	3	0.4		
Hata3	26.94	8	3.4		

Ek Çizelge 22. Antepfistığı Verim Değerlerine İlişkin Varyans Analizi (2002)

Var. Kaynağı	KT	SD	KO	F	Ft
DT	1014.3				
GKT	683.9	31			
B KT	211.8	1	211.8		
N KT	40.0	3	13.3	1.67	
Hata1	24.0	3	8.0		
I KT	38.9	1	38.9	1.10	
I*N int	163.6	3	54.5	1.55	6.59
Hata2	141.0	4	35.3		
Kcp KT	3.08	1	3.1		
Kcp*I int	0.13	1	0.1		
Kcp*N int	14.44	3	4.8		
Kcp*I*N	16.56	3	5.5		
Hata3	30.42	8	3.8		

Ek Çizelge 23. Deneme Konularına İlişkin Meyve Kalite Özellikleri

KONULAR	ÇIT. ORANI (%)			100 DANE AĞR. (gr)			İÇ MEY.RAND. (%)		
	2000	2001	2002	2000	2001	2002	2000	2001	2002
7No60	57.5	67.3	61.3	99.3	118.5	114.7	40.5	41.9	37.84
7No90	64.3	75.0	48.7	99.7	113.1	120.2	42.6	41.5	37.34
$7N_1$ 60	70.2	43.2	52.0	106.2	127.3	115.1	40.7	42.1	37.82
$7N_1$ 90	76.7	86.3	56.5	107.5	103.6	117.9	42.0	41.8	37.75
$7N_2$ 60	60.7	70.2	51.0	104.3	111.4	121.3	42.3	41.5	37.99
$7N_2$ 90	52.0	61.7	35.3	98.5	123.0	121.9	42.7	41.3	37.17
$7N_3$ 60	79.5	71.3	40.7	108.0	112.2	118.1	43.0	42.1	36.24
$7N_3$ 90	69.0	81.0	45.2	109.5	121.9	117.8	43.3	42.3	37.70
14No60	53.2	76.0	54.0	103.7	110.4	115.7	41.0	40.6	37.32
14No90	79.7	76.2	44.8	111.7	105.9	122.9	41.5	40.7	37.74
$14N_1$ 60	57.7	78.2	52.7	113.8	109.0	119.0	39.3	41.3	38.36
$14N_1$ 90	71.8	75.8	57.3	108.7	114.9	118.4	42.4	41.1	37.73
$14N_2$ 60	81.2	81.2	46.3	111.2	109.8	128.6	41.0	41.6	36.92
$14N_2$ 90	72.2	76.0	43.2	105.0	107.0	121.4	42.5	41.3	37.44
$14N_3$ 60	50.5	75.5	52.5	108.3	116.0	122.3	41.2	42.0	36.88
$14N_3$ 90	57.7	70.7	42.2	110.0	113.7	125.3	42.3	41.2	38.16
Geleneksel	71.8	59.7	55.0	101.3	109.2	111.9	41.7	40.3	39.03

Ek Çizelge 24. Çıtlama Oranlarının Varyans Analizleri (2000-2002)

	KT	SD	KO	F	Ft
DT	138767.9				
GKT	6311.4	31			
R KT	112.3	1	112.3		
N KT	147.7	3	49.2	0.41	
Hata1	363.8	3	121.3		
I KT	4.5	1	4.5	0.03	
I*N int	1847.9	3	616.0	4.28	6.59
Hata2	576.1	4	144.0		
Kcp KT	135.9	1	135.9		
Kcp*I int	249.8	1	249.8		
Kcp*N int	795.5	3	265.2		
Kcp*I*N	129.1	3	43.0		
Hata3	1949.0	8	243.6		

	KT	SD	KO	F	Ft
DT	169817.7				
GKT	3610.0	31			
R KT	32.4	1	32.41		
N KT	63.6	3	21.19	0.96	
Hata1	66.0	3	21.99		
I KT	356.9	1	356.91	5.08	
I*N int	329.9	3	109.96	1.56	6.59
Hata2	281.3	4	70.32		
Kcp KT	197.7	1	197.69		
Kcp*I int	513.6	1	513.63		
Kcp*N int	769.1	3	256.36		
Kcp*I*N	657.3	3	219.12		
Hata3	342.2	8	42.78		

	KT	SD	KO	F	Ft
DT	76766.68				
GKT	2971.54	31			
R KT	455.01	1	455.01		
N KT	658.93	3	219.64	3.51	
Hata1	187.82	3	62.61		
I KT	0.68	1	0.68	0.00	
I*N int	106.82	3	35.61	0.25	6.59
Hata2	561.72	4	140.43		
Kcp KT	174.22	1	174.22		
Kcp*I int	0.22	1	0.22		
Kcp*N int	300.50	3	100.17		
Kcp*I*N	194.06	3	64.69		
Hata3	331.56	8	41.44		

Ek Çizelge 25. 100 Dane Ağırlıklarının Varyans Analizleri (2000-2002)

	KT	SD	KO	F	Ft
DT	363534.4				
GKT	1615.3	31			
R KT	224.4	1	224.37		
N KT	192.1	3	64.04	5.28	
Hata1	36.4	3	12.14		
I KT	193.1	1	193.06	2.65	
I*N int	68.1	3	22.71	0.31	6.59
Hata2	291.0	4	72.75		
Kcp KT	2.3	1	2.31		
Kcp*I int	0.1	1	0.12		
Kcp*N int	116.4	3	38.79		
Kcp*I*N	50.5	3	16.83		
Hata3	440.9	8	55.12		

	KT	SD	KO	F	Ft
DT	412951.15				
GKT	2032.71	31			
R KT	93.84	1	93.84		
N KT	70.81	3	23.60	2.46	
Hata1	28.75	3	9.58		
I KT	243.84	1	243.84	16.59	
I*N int	60.05	3	20.02	1.36	6.59
Hata2	58.79	4	14.70		
Kcp KT	17.03	1	17.03		
Kcp*I int	2.06	1	2.06		
Kcp*N int	257.27	3	85.76		
Kcp*I*N	611.76	3	203.92		
Hata3	588.51	8	73.56		

	KT	SD	KO	F	Ft
DT	461023.23				
GKT	1230.69	31			
R KT	121.04	1	121.04		
N KT	158.79	3	52.93	0.657	
Hata1	241.57	3	80.52		
I KT	89.66	1	89.66	1.080	
I*N int	19.68	3	6.56	0.079	6.59
Hata2	331.93	4	82.98		
Kcp KT	15.33	1	15.33		
Kcp*I int	4.79	1	4.79		
Kcp*N int	94.23	3	31.41		
Kcp*I*N	38.59	3	12.86		
Hata3	115.08	8	14.39		

Ek Çizelge 26. İç Meyve Randımanlarına İlişkin Varyans Analizleri (2000-2002)

	KT	SD	KO	F	Ft
DT	55821.63				
GKT	58.60	31			
R KT	1.61	1	1.61		
N KT	9.13	3	3.04	13.06	
Hata1	0.70	3	0.23		
I KT	4.55	1	4.55	12.66	
I*N int	1.30	3	0.43	1.20	6.59
Hata2	1.44	4	0.36		
Kcp KT	13.54	1	13.54		
Kcp*I int	0.57	1	0.57		
Kcp*N int	2.66	3	0.89		
Kcp*I*N	3.19	3	1.06		
Hata3	19.93	8	2.49		

	KT	SD	KO	F	Ft
DT	55151.68				
GKT	21.81	31			
R KT	0.14	1	0.14		
N KT	2.09	3	0.70	0.31	
Hata1	6.68	3	2.23		
I KT	2.69	1	2.69	4.58	
I*N int	1.24	3	0.41	0.70	6.59
Hata2	2.35	4	0.59		
Kcp KT	0.45	1	0.45		
Kcp*I int	0.01	1	0.01		
Kcp*N int	0.01	3	0.00		
Kcp*I*N	0.74	3	0.25		
Hata3	5.39	8	0.67		

	KT	SD	KO	F	Ft
DT	45061.58				
GKT	18.28	31			
R KT	0.23	1	0.23		
N KT	2.01	3	0.67	0.64	
Hata1	3.14	3	1.05		
I KT	0.06	1	0.06	0.07	
I*N int	1.00	3	0.33	0.35	6.59
Hata2	3.85	4	0.96		
Kcp KT	0.34	1	0.34		
Kcp*I int	0.29	1	0.29		
Kcp*N int	3.67	3	1.22		
Kcp*I*N	1.20	3	0.40		
Hata3	2.49	8	0.31		

Ek Çizelge 27. Geleneksel konu ile diğer konuların Meyve Kalite Özellikleri yönünden varyans analizi ile karşılaştırılması

**Çıtlama Oranı**						
2000	SD	KT	KO	F	Fc, % 5	Fc, % 1
Geleneksel dışındaki KA	15	3310.28	220.69	1.25	2.31	3.31
Geleneksel-Diğer Konular	1	67.34	67.34	0.38	4.45	8.40
Hata	17	3007.84	176.93			
Genel	33	6385.46				
2001	SD	KT	KO	F	Fc, % 5	Fc, % 1
Geleneksel dışındaki KA	15	2888.12	192.54	4.45**	2.31	3.31
Geleneksel-Diğer Konular	1	327.04	327.04	7.55*	4.45	8.40
Hata	17	736.09	43.30			
Genel	33	3951.26				
2002	SD	KT	KO	F	Fc, % 5	Fc, % 1
Geleneksel dışındaki KA	15	1435.43	95.70	1.01	2.31	3.31
Geleneksel-Diğer Konular	1	68.24	68.24	0.72	4.45	8.40
Hata	17	1608.11	94.59			
Genel	33	3111.78				

**100 Dane Ağırlığı**						
2000	SD	KT	KO	F	Fc, % 5	Fc, % 1
Geleneksel dışındaki KA	15	622.59	41.51	0.66	2.31	3.31
Geleneksel-Diğer Konular	1	51.92	51.92	0.83	4.45	8.40
Hata	17	1064.71	62.63			
Genel	33	1739.23				
2001	SD	KT	KO	F	Fc, % 5	Fc, % 1
Geleneksel dışındaki KA	15	1262.82	84.19	1.86	2.31	3.31
Geleneksel-Diğer Konular	1	36.95	36.95	0.82	4.45	8.40
Hata	17	769.89	45.29			
Genel	33	2069.66				
2002	SD	KT	KO	F	Fc, % 5	Fc, % 1
Geleneksel dışındaki KA	15	421.06	28.07	0.48	2.31	3.31
Geleneksel-Diğer Konular	1	124.34	124.34	2.14	4.45	8.40
Hata	17	989.81	58.22			
Genel	33	1535.21				

Ek Çizelge 27' nin devamı

İç Meyve Randımanı						
2000	SD	KT	KO	F	Fc, % 5	Fc, % 1
Geleneksel dışındaki KA	15	34.93	2.33	1.64	2.31	3.31
Geleneksel-Diğer Konular	1	0.0007	0.0007	0.0005	4.45	8.40
Hata	17	24.15	1.42			
Genel	33	59.08				
2001	SD	KT	KO	F	Fc, % 5	Fc, % 1
Geleneksel dışındaki KA	15	7.24	0.48	0.56	2.31	3.31
Geleneksel-Diğer Konular	1	2.67	2.67	3.09	4.45	8.40
Hata	17	14.70	0.86			
Genel	33	24.61				
2002	SD	KT	KO	F	Fc, % 5	Fc, % 1
Geleneksel dışındaki KA	15	8.58	0.57	0.71	2.31	3.31
Geleneksel-Diğer Konular	1	4.26	4.26	5.27*	4.45	8.40
Hata	17	13.73	0.81			
Genel	33	26.57				

Ek Çizelge 28. Deneme Konularının KDO-Verim İlişkisi (1999)

Konular	Kasım	% Dökülme	1-%	Haziran	Verim(2000)
$7N_0 60$	21.42	0.31	0.69	31.00	15.79
$7N_0 90$	23.67	0.33	0.67	35.58	14.12
$7N_1$ 60	18.83	0.36	0.64	29.42	2.96
$7N_1$ 90	18.83	0.36	0.64	29.42	7.44
$7N_2$ 60	65.58	0.34	0.66	99.58	6.68
$7N_2$ 90	29.50	0.30	0.70	42.42	8.14
$7N_3$ 60	22.50	0.34	0.66	33.92	14.15
$7N_3$ 90	22.50	0.34	0.66	33.92	16.02
$14N_0 60$	21.42	0.31	0.69	31.00	3.86
$14N_0 90$	23.67	0.33	0.67	35.58	11.00
$14N_1$ 60	41.08	0.33	0.67	61.17	9.55
$14N_1$ 90	2.08	0.50	0.50	4.17	5.99
$14N_2$ 60	25.58	0.37	0.63	40.42	19.95
$14N_2$ 90	25.17	0.37	0.63	39.67	16.69
$14N_3$ 60	27.58	0.31	0.69	40.25	4.82
$14N_3$ 90	39.42	0.35	0.65	61.00	7.80
Geleneksel	26.17	0.37	0.63	41.3	4.51
Ortalama		0.35			9.97

Ek Çizelge 29. Deneme Konularının KDO-Verim İlişkisi (2000)

2000/Konular	Kasım	% Dökülme	1-%	Haziran	Verim(2001)
$7N_060$	9.67	0.27	0.73	13.25	7.00
$7N_090$	4.50	0.44	0.56	8.00	6.91
$7N_1$ 60	10.67	0.21	0.79	13.50	9.96
$7N_1$ 90	7.75	0.38	0.62	12.42	8.34
$7N_2$ 60	1.25	0.83	0.17	7.17	10.37
$7N_2$ 90	1.58	0.62	0.38	4.17	9.00
$7N_3$ 60	2.50	0.58	0.42	6.00	4.69
$7N_3$ 90	4.67	0.51	0.49	9.58	4.58
$14N_060$	7.33	0.35	0.65	11.25	11.74
$14N_090$	4.67	0.40	0.60	7.75	12.29
$14N_1$ 60	1.92	0.49	0.51	3.75	8.57
$14N_1$ 90	10.75	0.20	0.80	13.50	7.35
$14N_2$ 60	4.00	0.59	0.41	9.75	5.73
$14N_2$ 90	3.50	0.49	0.51	6.92	5.45
$14N_3$ 60	15.67	0.17	0.83	18.92	10.56
$14N_3$ 90	7.75	0.48	0.52	15.00	12.85
Geleneksel	8.59	0.30	0.70	12.33	9.10
Ortalama		0.43			8.50

Ek Çizelge 30. Deneme Konularının KDO-Verim İlişkisi (2001)

2001/Konular	Kasım	% Dökülme	1-%	Haziran	Verim(2002)
$7N_0 60$	40.25	0.21	0.79	51.25	13.38
$7N_0 90$	37.50	0.27	0.73	51.50	9.90
$7N_1$ 60	15.25	0.46	0.54	28.50	4.21
$7N_1$ 90	34.25	0.13	0.87	39.50	3.57
$7N_2$ 60	8.25	0.37	0.63	13.00	4.26
$7N_2$ 90	23.25	0.26	0.74	31.50	3.41
$7N_3$ 60	19.75	0.25	0.75	26.50	6.58
$7N_3$ 90	12.75	0.79	0.21	60.25	8.56
$14N_0 60$	7.75	0.81	0.19	40.25	2.70
$14N_0 90$	2.50	0.62	0.38	6.50	3.78
$14N_1$ 60	12.00	0.55	0.45	26.50	6.82
$14N_1$ 90	0.75	0.98	0.02	40.75	2.92
$14N_2$ 60	39.25	0.08	0.92	42.50	6.93
$14N_2$ 90	38.75	0.10	0.90	43.00	6.93
$14N_3$ 60	29.75	0.57	0.43	68.50	2.66
$14N_3$ 90	7.50	0.88	0.12	63.75	3.50
Geleneksel	5.13	0.78	0.22	23.38	2.80
Ortalama		0.48			5.46

Ek Çizelge 31. Deneme Konularının KDO-Verim İlişkisi (2002)

2002/ Konular	Kasım	% Dökülme	1-%	Haziran	Verim(2003)
$7N_0$60	18.7	0.69	0.31	60.3	15.71
$7N_0$90	20.0	0.61	0.39	51.6	17.10
$7N_1$ 60	18.4	0.68	0.32	57.9	15.78
$7N_1$ 90	31.0	0.60	0.40	76.6	17.91
$7N_2$ 60	20.0	0.69	0.31	64.1	21.76
$7N_2$ 90	48.0	0.38	0.62	77.8	20.17
$7N_3$ 60	20.0	0.64	0.36	55.7	19.73
$7N_3$ 90	23.7	0.62	0.38	62.6	18.14
$14N_0$60	24.1	0.62	0.38	63.1	22.68
$14N_0$90	44.0	0.39	0.61	71.8	32.09
$14N_1$ 60	9.8	0.84	0.16	60.8	14.79
$14N_1$ 90	30.3	0.39	0.61	49.5	16.08
$14N_2$ 60	20.4	0.65	0.35	58.6	26.90
$14N_2$ 90	18.8	0.55	0.45	41.8	24.17
$14N_3$ 60	43.3	0.53	0.47	91.8	12.85
$14N_3$ 90	35.6	0.55	0.45	78.8	24.45
Geleneksel	26.7	0.58	0.42	63.0	17.92
Ortalama		0.59			19.90

Ek Çizelge 32. Oransal ET Açığı ile Oransal Verim Azalması Değerleri

ET	Y	Yd	1-Etc/Etm	1-Ya/Ym	1-Yd/Ym
642.1	9.8	7.76	0	0	0.00
590.2	9.0	7.26	0.08	0.08	0.06
528.1	3.5	6.66	0.18	0.64	0.14
517.8	4.6	6.57	0.19	0.53	0.15
497.1	6.6	6.37	0.23	0.33	0.18
483.3	2.7	6.23	0.25	0.72	0.20
400.4	7.8	5.44	0.38	0.20	0.30
400.1	4.7	5.44	0.38	0.52	0.30
389.1	6.0	5.33	0.39	0.39	0.31
383.8	5.9	5.28	0.40	0.40	0.32
377.4	5.8	5.22	0.41	0.41	0.33
376.0	5.8	5.20	0.41	0.41	0.33
345.2	5.3	4.91	0.46	0.46	0.37
317.8	4.8	4.65	0.51	0.51	0.40
288.3	4.5	4.36	0.55	0.54	0.44
259.8	4.1	4.09	0.60	0.58	0.47
239.2	3.7	3.89	0.63	0.62	0.50
212.8	3.4	3.64	0.67	0.65	0.53
197.7	4.5	3.49	0.69	0.54	0.55
91.5	1.5	2.47	0.86	0.85	0.68

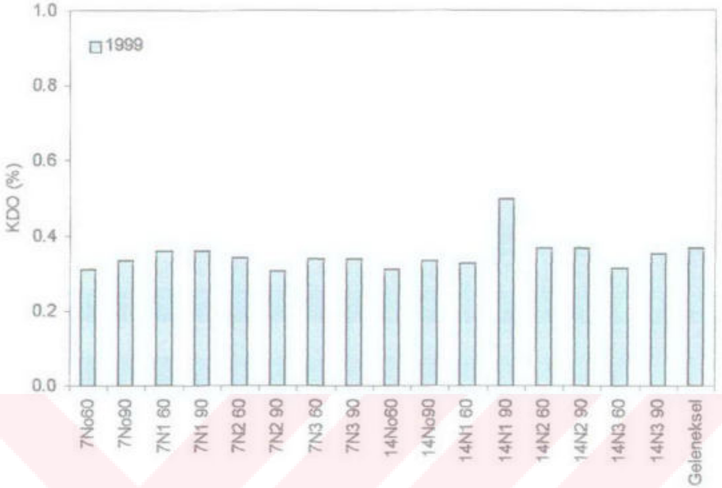

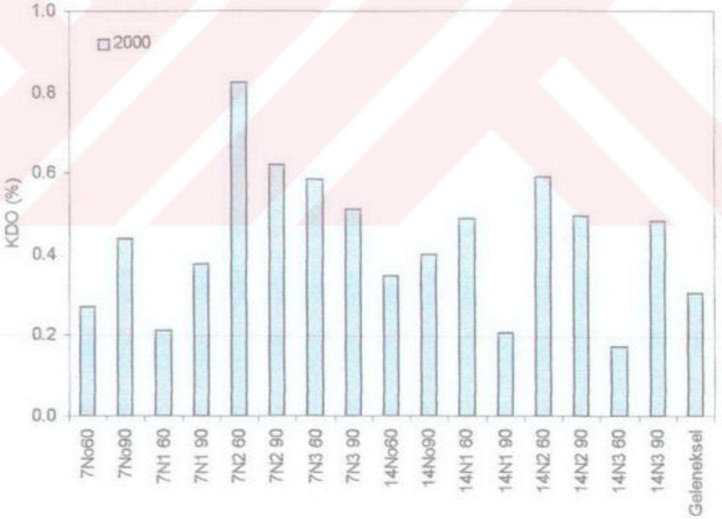

Ek Şekil 1. Deneme Konularının Karagöz Dökülme Oranları (1999 ve 2000)

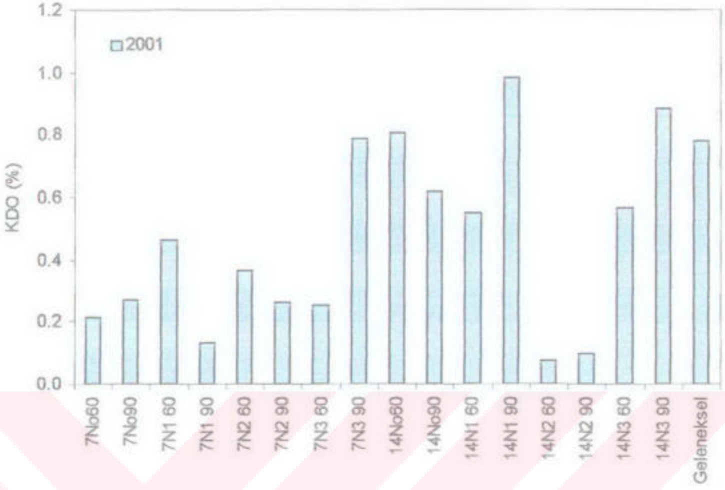

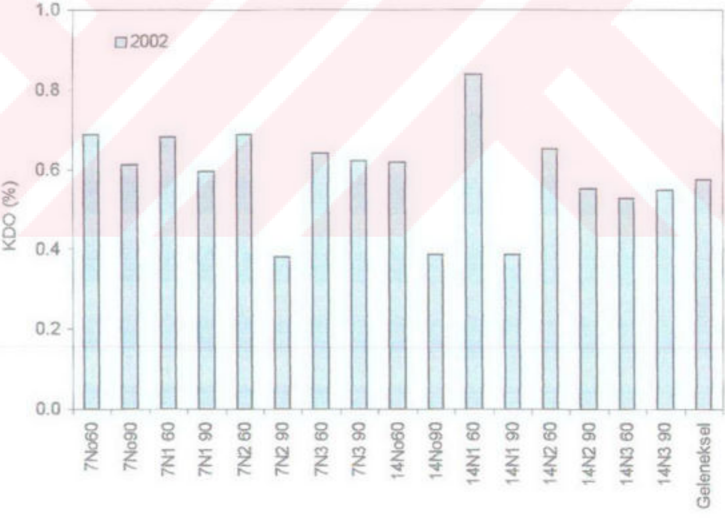

Ek Şekil 2. Deneme Konularının Karagöz Dökülme Oranları (2001 ve 2002)
